# Using patterns of shared taxa to infer bacterial dispersal in human living environment in urban and rural areas

**DOI:** 10.1128/aem.00903-24

**Published:** 2024-09-04

**Authors:** M. Grönroos, A. Jumpponen, M. I. Roslund, N. Nurminen, S. Oikarinen, A. Parajuli, O. H. Laitinen, O. Cinek, L. Kramna, J. Rajaniemi, H. Hyöty, R. Puhakka, A. Sinkkonen

**Affiliations:** 1 Faculty of Biological and Environmental Sciences, University of Helsinki, Lahti, Finland; 2 Division of Biology and Ecological Genomics Institute, Kansas State University, Manhattan, Kansas, USA; 3 Natural Resources Institute Finland, Helsinki, Finland; 4 Faculty of Medicine and Health Technology, Tampere University, Tampere, Finland; 5 Department of Medicine, Karolinska Institutet, Huddinge, Sweden; 6 Department of Medical Microbiology, 2nd Faculty of Medicine, Charles University, Prague, Czech Republic; 7 Faculty of Built Environment, Tampere University, Tampere, Finland; 8 Fimlab Laboratories, Pirkanmaa Hospital District, Tampere, Finland; University of Illinois Urbana-Champaign, Urbana, Illinois, USA

**Keywords:** bacteria, biodiversity hypothesis, dispersal, hygiene hypothesis, land cover

## Abstract

Contact with environmental microbial communities primes the human immune system. Factors determining the distribution of microorganisms, such as dispersal, are thus important for human health. Here, we used the relative number of bacteria shared between environmental and human samples as a measure of bacterial dispersal and studied these associations with living environment and lifestyles. We analyzed amplicon sequence variants (ASVs) of the V4 region of 16S rDNA gene from 347 samples of doormat dust as well as samples of saliva, skin swabs, and feces from 53 elderly people in urban and rural areas in Finland at three timepoints. We first enumerated the ASVs shared between doormat and one of the human sample types (i.e., saliva, skin swab, or feces) of each individual subject and calculated the shared ASVs as a proportion of all ASVs in the given sample type of that individual. We observed that the patterns for the proportions of shared ASVs differed among seasons and human sample type. In skin samples, there was a negative association between the proportion of shared ASVs and the coverage of built environment (a proxy for degree of urbanization), whereas in saliva data, this association was positive. We discuss these findings in the context of differing species pools in urban and rural environments.

**IMPORTANCE:**

Understanding how environmental microorganisms reach and interact with humans is a key question when aiming to increase human contacts with natural microbiota. Few methods are suitable for studying microbial dispersal at relatively large spatial scales. Thus, we tested an indirect method and studied patterns of bacterial taxa that are shared between humans and their living environment.

## INTRODUCTION

Both theoretical and empirical studies indicate that microbiota within and around us strongly affect the human immune system and health [reviewed in references (1, 2)]. Contact with diverse microbial communities, especially early in life, primes the human immune system to work correctly (3). Hygiene and biodiversity hypotheses suggest that Western lifestyle, urbanized living environment, and decreasing biodiversity have decreased contact with diverse microbiota, contributing to the increasing abundance of non-communicable diseases such as allergy and asthma (4–7). Factors determining the distribution of microorganisms are thus important for human health.

Organismal distribution in space and time is governed by four fundamental processes: speciation, drift, selection, and dispersal (8). In microbial communities, speciation (or diversification) can occur at very short time scales, for instance, when new species or forms emerge via mutations or horizontal gene transfer (9). Ecological drift refers to the stochastic changes in species abundance. In microbial communities, most taxa occur in low abundances and rare taxa are particularly vulnerable to random extinctions [i.e., drift (10)]. Deterministic selection driven by organismal and environmental differences is commonly recognized as differences in microbial communities at adjacent but environmentally distinct sites, such as the dry skin of human elbows and knees compared to moist skin of the bends of elbows and knees (11). Finally, dispersal allows the movement of organisms from one location to another (8).

In the context of biodiversity hypothesis (7), dispersal can be considered especially important: how do microorganisms in natural environments disperse and reach humans and their living environment? Dispersal is complex. First, the regional species pools determine the species assemblages that have the potential to disperse to local communities (12, 13). When considering a human as the local site for microbes, a region can be considered as the geographical area where this individual is dwelling. Composition of the regional microbial species pool depends, among other things, on land cover and degree of urbanization (5). Rural areas often have more abundant and more diverse aerial microbial communities than urban areas (14, 15). Bacterial communities also differ among indoor spaces; e.g., homes are enriched with human-associated bacteria compared to barns (16), and house plants affect microbial communities of a home and its residents (17, 18). Second, microorganisms differ in their dispersal ability; e.g., microbes with dormant stages may have higher dispersal potential than those without (19). Third, especially for microorganisms that rely on passive dispersal, environmental factors such as wind (20) or animal vectors (21) are important. For human microbiota, lifestyles interact with potential for microbial dispersal. Particularly, the number of social contacts, cohabiting humans and non-humans (22), quantity and quality of time spent outdoors (23–25), and the season likely affect the human microbiome assembly.

*In situ* measurements of dispersal of microorganisms are difficult if not impossible at large spatial scales. Thus, several indirect, pattern-based approaches have been employed to study their dispersal (26–28). Previous studies have reported that residents share bacterial communities with home surfaces to the extent that the occupants leave distinct microbial fingerprints on the surfaces (29). Data also suggest that bacterial dispersal from humans to indoor surfaces is central for bacterial community transfer (30). These studies, however, have focused on surfaces that are not especially collecting outdoor microbes, such as doorknobs and chairs. In contrast, in our three previous studies, we sampled bacteria from doormats that collected soil carried home from outdoors via home occupants’ shoes and feet (31–33). A common custom in Finland is to brush the soles of shoes on a doormat and then leave the outdoor shoes in the hallway. This makes the doormat an optimal collector of outdoor dust and soil, along with the environmental microbes that have the potential to disperse on and into the human residents. Based on Moquet and Loreau’s (34) seminal contribution, high dispersal should homogenize communities. Therefore, we postulate that the higher the dispersal between the study subject and his/her surroundings, the higher the relative number of bacterial taxa shared between human samples and doormats.

**Fig 1:**
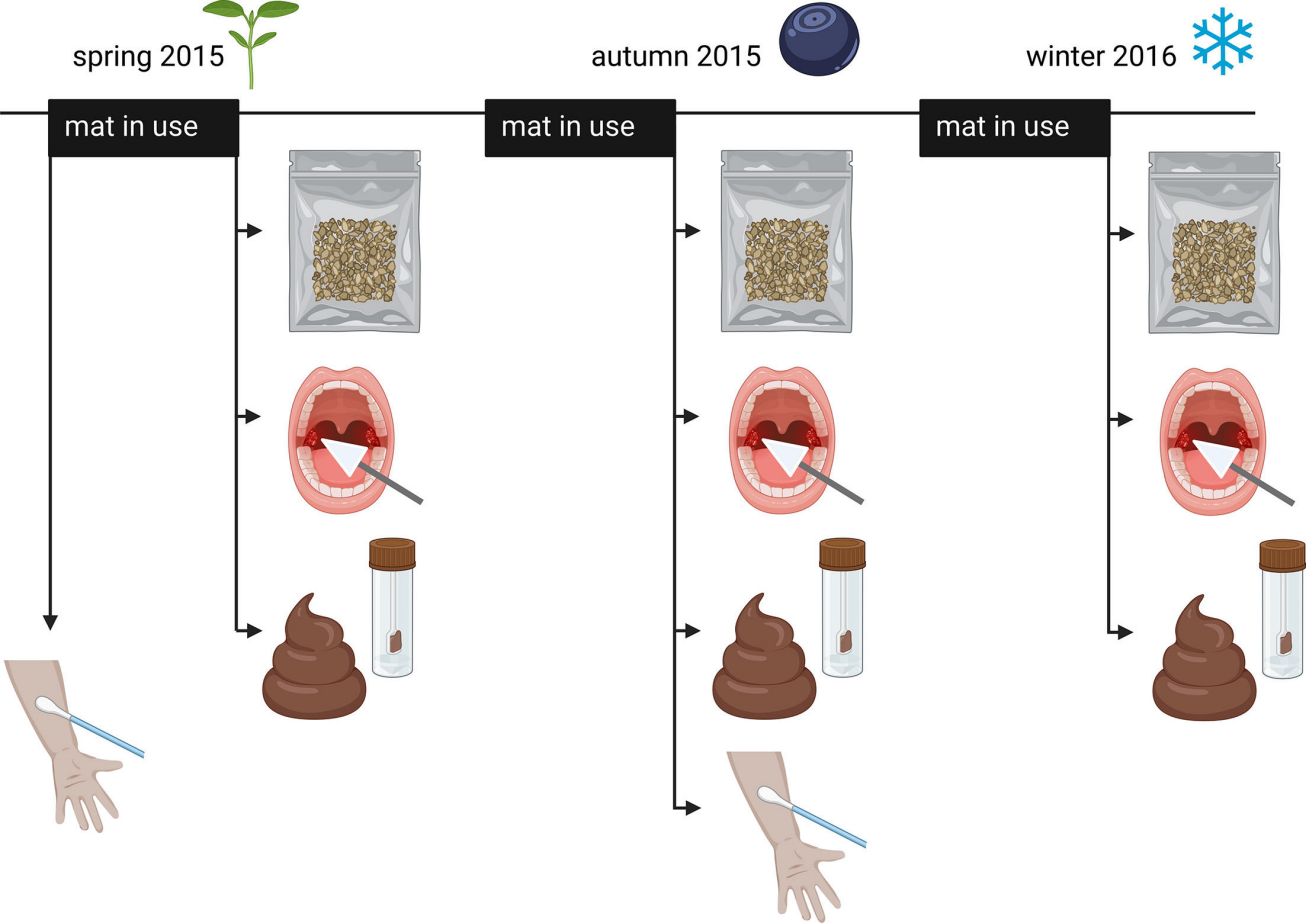
Sampling. Illustration summarizing the sampling protocol. Soil deposited on participants’ doormats was collected into zipper bags (gray bags on the top). From the same study participants, skin swabs and saliva samples were collected using sterile cotton wool sticks. Fecal samples were collected into special fecal sample tubes. In spring, skin swab samples were taken before use of the doormats, but all the other samples were taken at the same time after the use of doormats (see Materials and Methods). Illustration created with BioRender.com.

We analyzed data of a total of 347 bacterial samples of soil deposited on doormats and of human saliva, skin swab, and fecal samples (Fig. 1). These data were collected from 53 elderly people and their residences in the city of Lahti in southern Finland and surrounding countryside. To account for seasonal variation, we sampled at three timepoints: in spring and autumn 2015 and winter 2016 (except skin swabs). Bacterial DNA was extracted, and the hypervariable V4 region within the 16S rDNA was sequenced. We first enumerated bacterial amplicon sequence variants (ASVs, i.e., sequences with at least 99% similarity) that were shared between doormat and one of the human sample types (saliva, skin swab, or feces). As the number of ASVs likely affects the number of shared ASVs, we then calculated the proportion of shared ASVs of the total number of ASVs in each human sample and used this proportion as an indicator of dispersal potential within homes.

Previously, we have reported that the total volume of soil deposited on the doormats, as well as the bacterial richness, is inversely associated with the proportion of built environment in the surroundings of permanent residences in Finland (33). Many studies have related human microbiota with land cover [e.g., see references (35, 36)] and have shown that farm children often have more diverse microbiota than urban dwellers (37), although this relationship is complex (38). Here we build on our previous studies and explore the bacterial dispersal and its association with land cover. Little is known about how urbanization affects bacterial dispersal, a factor key to bacterial transfer from the living environment to human residents. Thus, here we test whether urbanization *per se* influences bacterial dispersal.

In addition to land cover or urbanization, living conditions and lifestyles may affect the degree of bacterial dispersal into and within homes. For example, exposure to outdoor environment is likely to affect bacterial dispersal. Thus, we hypothesize that time spent for outdoor recreation in general, or specifically, for gardening is positively associated with the proportion of shared microbiome. Also, indoor pets can affect the bacterial dispersal into and within homes. Thus, pets can provide an additional source of microbiota by adding their own individual microbial community into residents’ home (22) or they can act as dispersal vectors by either bringing outdoor microbiota indoors (e.g., dogs) or circulate the microbiota when wandering within the home and being in contact with human residents. Similarly, the number of residents and visitors may add shared dispersal sources and thus increase the introduction and circulation of microbes indoors. Hands are potentially a crucial dispersal vector between a human and the environment (39). As the handwashing frequency is likely to affect the hand microbiome, we further hypothesize that frequent handwashing will reduce dispersal, i.e., the number of shared bacterial ASVs between the environment and skin.

To summarize, we evaluated whether built environment is associated with the proportion of bacterial ASVs shared between human and doormat samples within the household. We also hypothesize that:

1. The proportion of bacterial ASVs shared between human and doormat samples is higher in the presence of an indoor pet.
2. The proportion of bacterial ASVs shared between human and doormat samples is lower for those washing hands often.
3. The following variables are positively associated with the proportion of shared bacteria: outdoor recreation, gardening, number of days the doormat has been in use, and number of persons living and visiting the home.

## RESULTS

**Fig 2:**
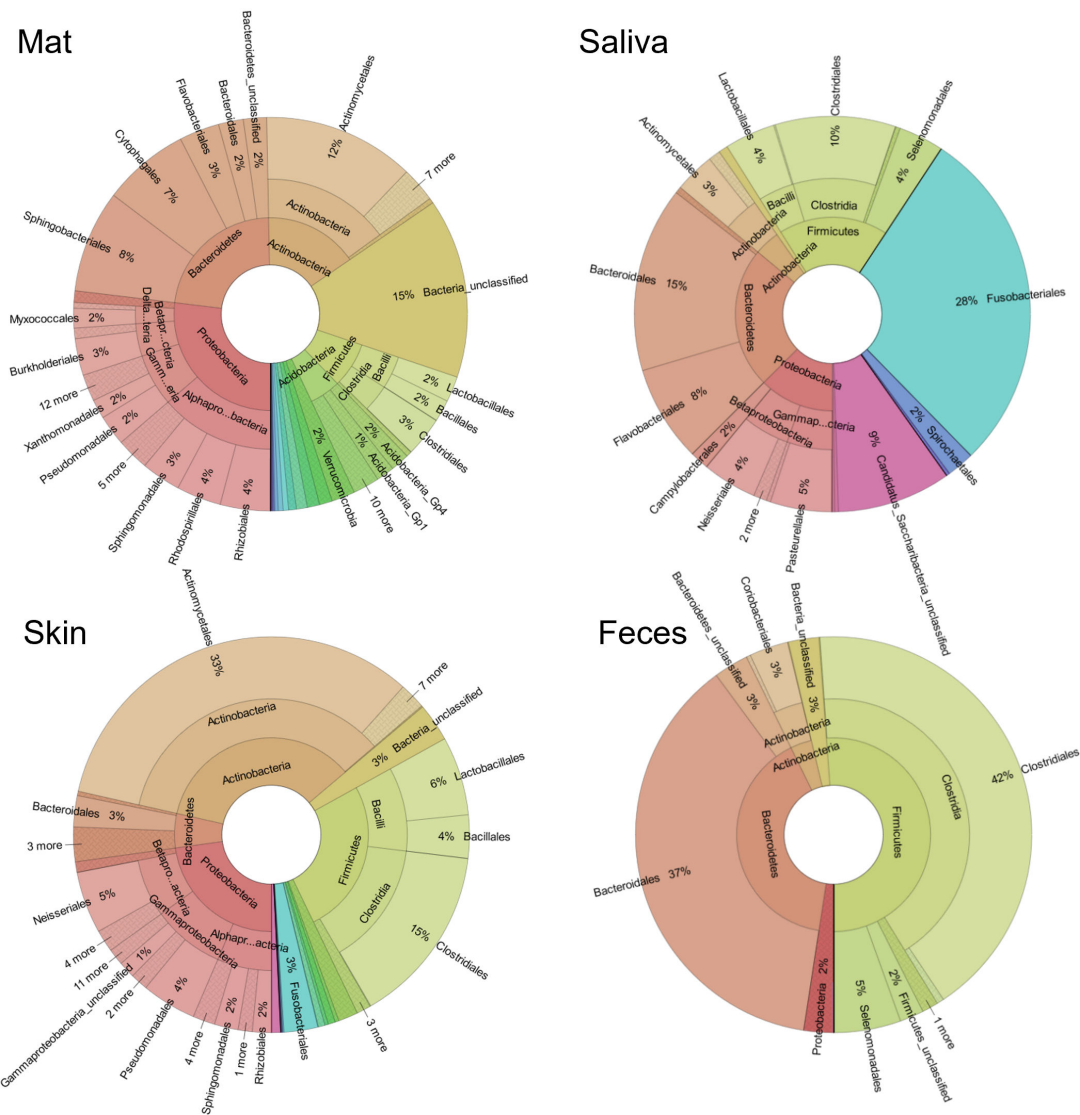
Relative abundance of bacterial taxa. Relative abundance of bacterial phyla, classes, and orders in doormat, saliva, skin swab, and fecal samples in the spring data (June 2015). Only study subjects (*N* = 19) with all sample types available are included. The figure was produced with KRONA (40). Interactive figures showing proportions also at finer taxonomic levels (up to ASV level) are provided in the supplement (Appendix A).

Total number of ASVs ranged from 55 to 218 in saliva, from 117 to 585 on the skin, from 44 to 356 in feces, and from 283 to 894 on doormat samples (see Table 3). Doormat samples had the highest diversity (Fig. 2). The four most abundant phyla in doormat samples were Proteobacteria (27%), Bacteroidetes (23%), Actinobacteria (16%), and Firmicutes (8%). As many as 15% of reads remained unclassified. In saliva samples, most abundant phyla were Fusobacteria (28%), Bacteroidetes (24%), Firmicutes (18%), and Proteobacteria (13%). Only 0.8% of these reads were unclassified. Skin swab samples were dominated by Actinobacteria (35%), followed by Firmicutes (26%), Proteobacteria (23%), and Bacteroidetes (6%). Skin contained several minor phyla, and 3% of reads remained unclassified. Fecal samples were strongly dominated by Firmicutes (51%) and Bacteroidetes (40%); a minority belonged to Actinobacteria (4%) and Proteobacteria (2%). In feces, 3% of reads remained unclassified at the phylum level.

### Community structure

**Fig 3:**
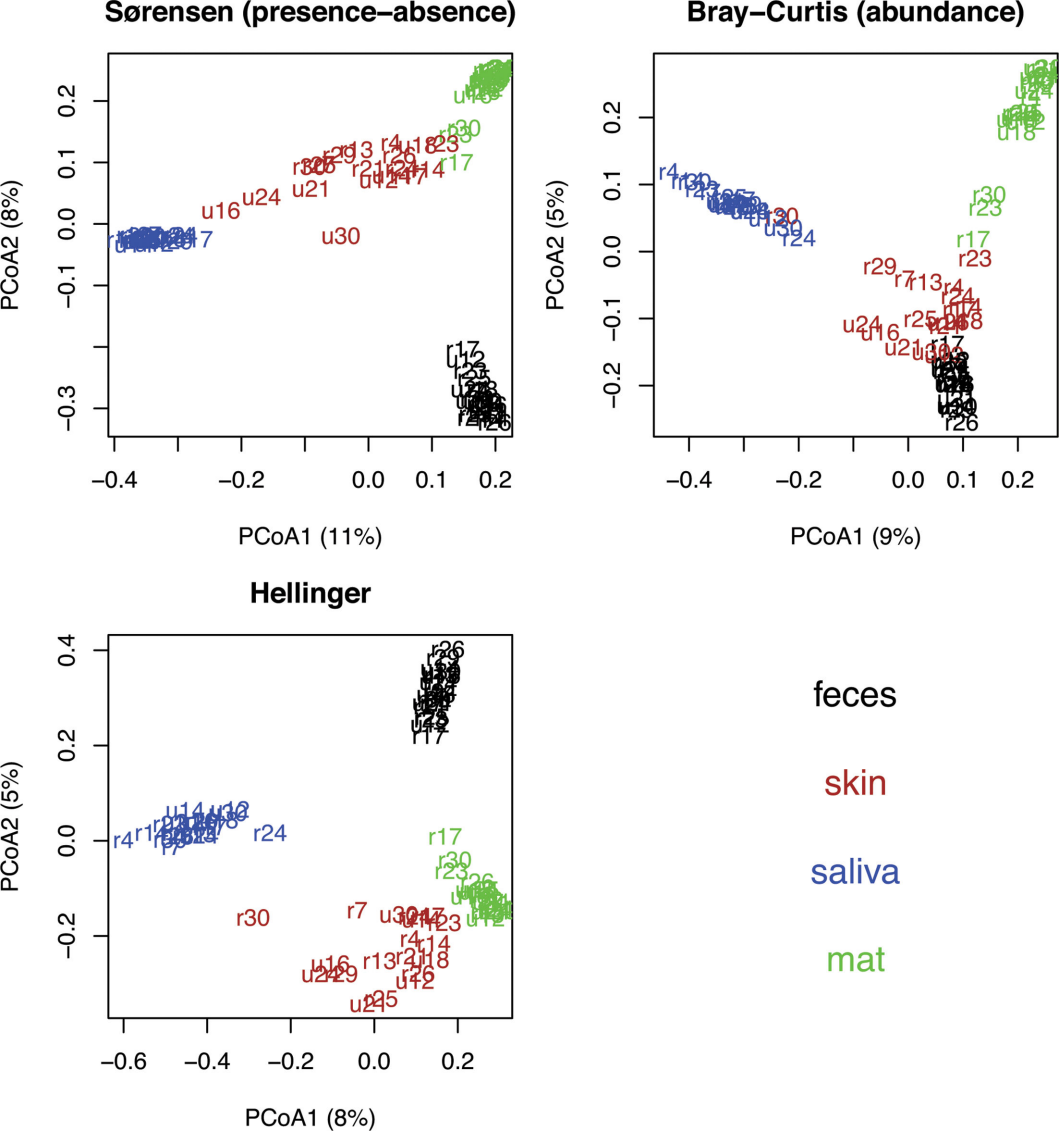
Principal coordinate analysis (PCoA) for all sample matrices. PCoA for all sample matrices (doormat, green; saliva, blue; skin, brown; feces, black) in the spring data (June 2015). We processed presence-absence data with the Bray-Curtis index a.k.a. Sørensen index, abundance data with Bray-Curtis index, and abundance data with Hellinger transformation and Euclidean distance. Only study subjects (*N* = 19) with all sample types available are included.

The sample types mainly clustered to distinct groups [Fig. 3; all permutational multivariate analyses of variance (PERMANOVAs), *P* = 0.001]. Although some overlap was evident when the first and second axes were plotted in principal coordinate analysis (PCoA, Fig. 3), the distinction was clear when the first axis was plotted against the third axis (Fig. S2). Variation captured even by the first axis was low (≤11%).

**Fig 4:**
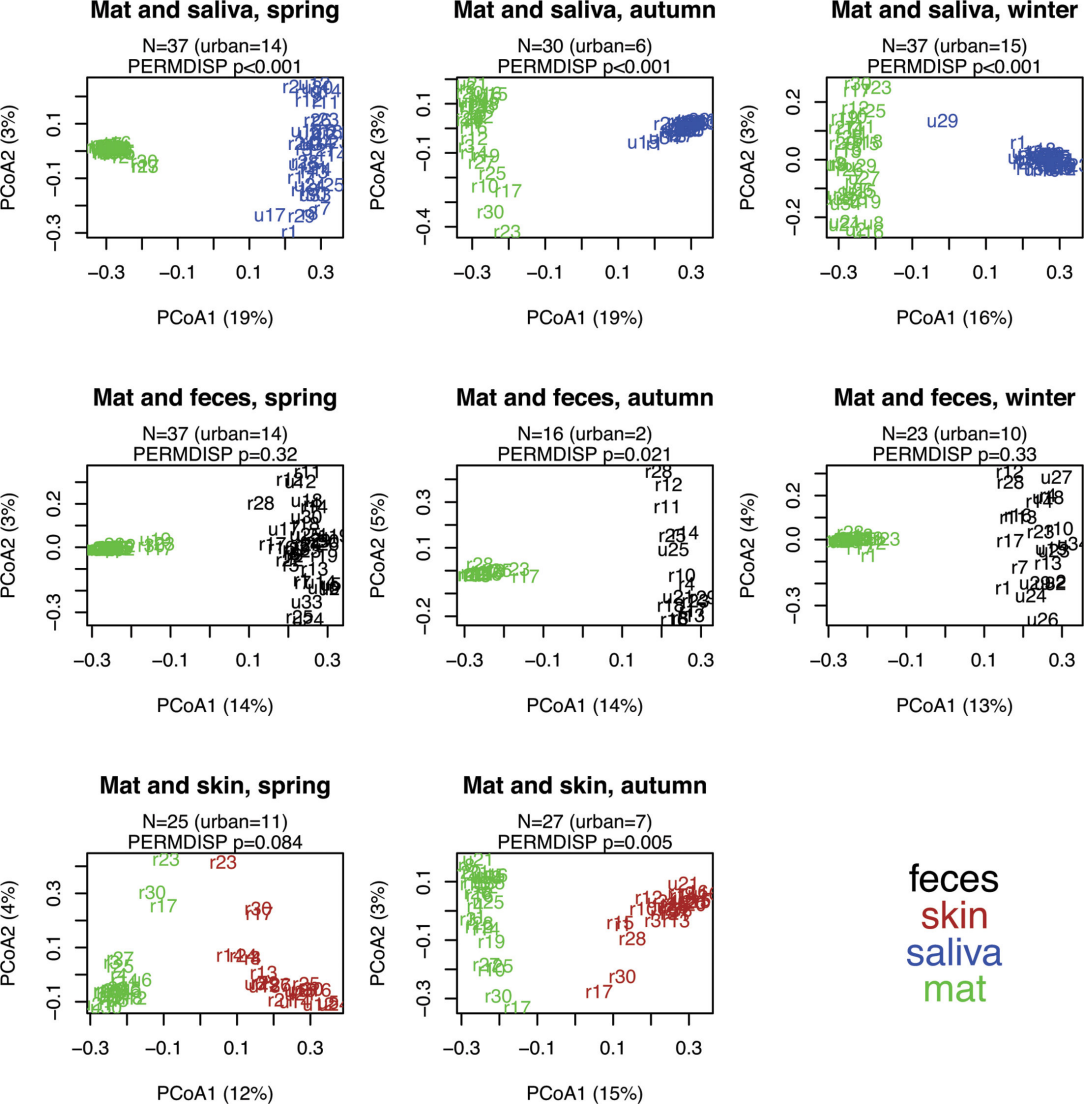
Principal coordinate analysis (PCoA) between environmental (doormat) and human samples. PCoA for environmental (doormat, green) and human samples (saliva, blue; skin, brown; and feces, black) in spring, autumn, and winter (winter was not available for skin samples). Numbers refer to study subjects, and the letter prior to the number refers to either urban (u) or rural (r). Only those study subjects who had both sample matrices available at each timepoint are included. The total number of study subjects (*N*) in each figure is given, as well as the number of urban subjects. Sørensen index (i.e., presence-absence data with Bray-Curtis distance) was used.

We plotted each human sample type at each timepoint together with doormat samples (Fig. 4). As expected, the first and most important PCoA axis separated samples by sample type (all PERMANOVAs, *P* = 0.001). Based on permutational test of multivariate homogeneity of group dispersions (PERMIDSP), human sample types showed sometimes lower and sometimes higher beta diversity compared to mat samples (Table 1). Saliva samples had always lower beta diversity than mat samples. This was also true for skin swab samples in the autumn. In contrast, fecal samples in autumn had higher beta diversity than mat samples. Variation explained by second PCoA axis was very low (≤5%). Interestingly, this axis seemed to simultaneously explain variation in doormat and skin swab samples. Especially, the skin swab bacteria of the rural study subject numbers r17, r23, and r30 appeared to correlate with their doormat bacteria (Fig 4.).

**TABLE 1:** Results of multivariate homogeneity of group dispersions (PERMDISP) comparing each of the human sample types to mat samples in each timepoint

	Average distance to median		PERMDISP ANOVA^*a*^						
	Mat	Human		Df	Sum Sq	Mean Sq	*F* value	Pr(>*F*)	*P* value^*b*^
Saliva spring	0.61	0.56	Groups	1	0.049	0.049	51.4	5.4E-10	***
			Residuals	72	0.068	0.001			
Saliva autumn	0.61	0.56	Groups	1	0.041	0.041	42.4	1.9E-08	***
			Residuals	58	0.056	0.001			
Saliva winter	0.63	0.57	Groups	1	0.055	0.055	41.2	1.3E-08	***
			Residuals	72	0.096	0.001			
Feces spring	0.61	0.62	Groups	1	0.001	0.001	1	0.3196	
			Residuals	72	0.059	0.001			
Feces autumn	0.60	0.62	Groups	1	0.003	0.003	5.94	0.02097	*
			Residuals	30	0.016	0.001			
Feces winter	0.63	0.62	Groups	1	0.001	0.001	0.97	0.3298	
			Residuals	44	0.033	0.001			
Skin spring	0.61	0.62	Groups	1	0.003	0.003	3.1	0.08443	
			Residuals	48	0.047	0.001			
Skin autumn	0.61	0.59	Groups	1	0.008	0.008	8.72	0.00471	**
			Residuals	52	0.050	0.001			

^*a*^ ANOVA, analysis of variance.

^*b*^ *, *P* < 0.05; **, *P* < 0.01; ***, *P* < 0.001.

### Shared taxa

**Fig 5:**
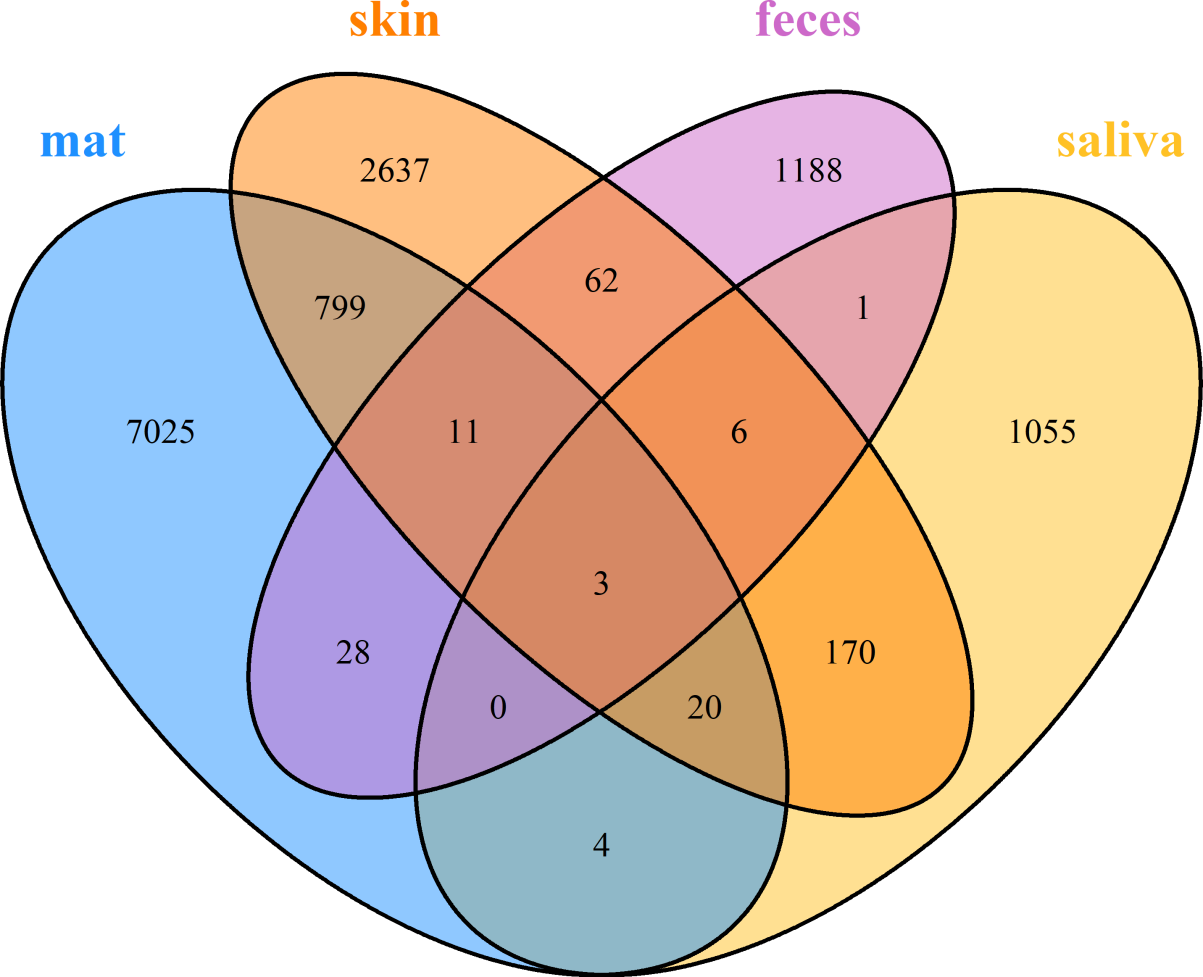
Venn diagram of samples in spring. Only study persons (*N* = 19) with all sample types available are included.

At the first timepoint (spring), all four sample types (i.e., mat, saliva, skin, and feces) were available from 19 study subjects. Venn diagram (Fig. 5) shows that the number of ASVs that were shared between skin and doormat samples was higher (833 ASVs in total) than the number of ASVs shared between doormat and feces (42 in total) and doormat and saliva (27 in total). Only three ASVs were present in all sample types. These three ASVs were assigned to *Lactobacillus*, Lachnospiraceae_unclassified, and Enterobacteriaceae_unclassified.

We enumerated the number of shared taxa between doormat samples and each of the human sample types. The number of these shared ASVs for each study subject varied from 0 to 18 in saliva data sets, from 0 to 228 in skin data sets, and from 0 to 48 in fecal data sets (see Table 3). Finally, we estimated the proportion of shared taxa from the total number in any given human sample type. This proportion varied between 0% and 17% in saliva, between 0% and 43% in the skin, and between 0% and 41% in feces and was on average highest in the skin (see Table 3; Fig. S2).

We used this proportion of shared taxa as a dependent variable in a generalized linear mixed-effects model (GLMM). Initial GLMMs were built for each explanatory variable separately (see flowchart in Fig. S1). These models showed associations especially for saliva and skin data (Tables S1a and b).

**Fig 6:**
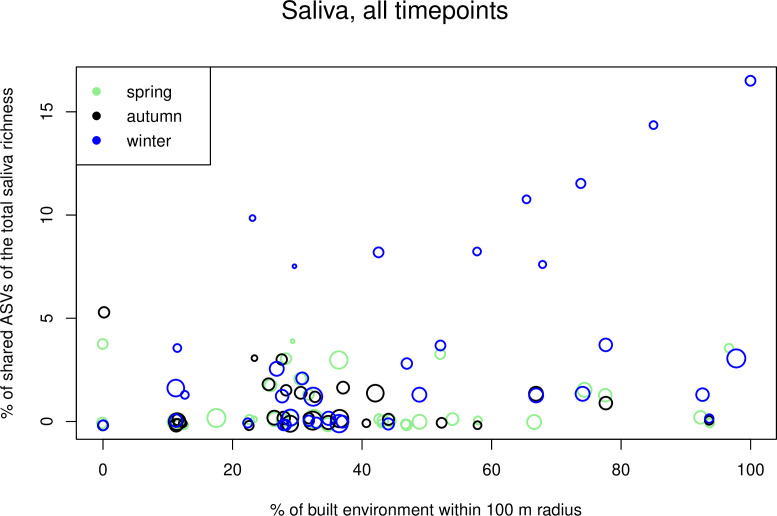
Scatter plot of most important variables in saliva GLMMs. Proportion of shared ASVs in saliva samples plotted against the coverage of built environment. Each timepoint is given in different colors. Point size is relative to the amount of time study subjects spend outdoors (outdoor mean; see Materials and Methods for description). Small amount of random noise was added to point coordinates to improve visualization. Note that for some study subjects, there are two to three observations in this graph, but for readability, they are not highlighted. In the GLMM models, the study subject was included as a random effect.

**Fig 7:**
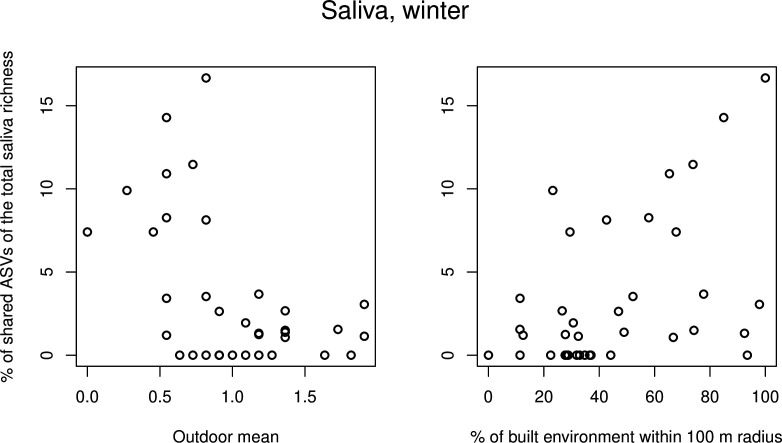
Scatter plots of most important variables in saliva GLMMs. Percentage of shared ASVs in saliva samples in winter plotted against the coverage of outdoor recreation and built environment.

For saliva data, initial GLMMs showed that the proportion of shared ASVs increased when the coverage of built environment increased (estimate 0.019, *P* = 0.006; Table S1a), wehen there were no indoor pets in the household (estimate −1.615, *P* = 0.014), when outdoor recreation decreased (estimate −1.252, *P* = 0.002), when participants did gardening less often (estimate −0.892, *P* = 0.025), and when there were fewer people living and visiting the house (estimate −0.260, *P* = 0.029; Table S1a). None of the variables showed significant interaction with the timepoint. The timepoint itself, however, was significant (*P* < 0.001; Table S1a), and on average, the proportion of shared ASVs was highest in winter (Fig. S3). After forward selection, the final model indicated that the proportion of shared ASVs increased when built environment increased but when the general amount of outdoor recreation decreased (Table 2; Fig. 6). When timepoints were analyzed separately, significant variables appeared only in winter data. This finding, together with inspection of the plot (Fig. 7), suggested that winter data caused the pattern for built environment in the model for all timepoints.

**TABLE 2:** Significant results of GLMM models after forward selection^*a*^

Dependent variable	Timepoints included	Explanatory variables selected in forward selection	*N*	Fixed effects	Estimate	SE	*z* value	Pr(>|*z*|)	*P* value	DHARMa
Proportion of shared ASVs in saliva	W	Built + outdoor	37	(Intercept)	−3.729	0.574	−6.497	<0.001	***	1
				Outdoor	−1.723	0.440	−3.912	<0.001	***	
				Built	0.028	0.007	3.868	<0.001	***	
	S, A, and W	Built + outdoor	104	(Intercept)	−4.591	0.477	−9.627	<0.001	***	1
				Outdoor	−1.276	0.355	−3.594	<0.001	***	
				Built	0.018	0.006	3.260	0.001	**	
				TimepointA	0.140	0.262	0.535	0.592		
				TimepointW	1.071	0.205	5.212	<0.001	***	
Proportion of shared ASVs on skin	S	Built	25	(Intercept)	−1.308	0.199	−6.559	<0.001	***	0
				Built	−0.012	0.004	−2.824	0.005	**	
	S and A	Real.mat.days × timepoint + gardening × timepoint + built	49	(Intercept)	−2.680	0.435	−6.154	<0.001	***	0
				Real.mat.days	0.092	0.031	2.984	0.003	**	
				TimepointA	0.934	0.393	2.373	0.018	*	
				GardeningMONTHLY	0.262	0.204	1.280	0.201		
				Built	−0.009	0.004	−2.478	0.013	*	
				Real.mat.days:timepointA	−0.079	0.030	−2.614	0.009	**	
				TimepointA:gardeningMONTHLY	−0.247	0.165	−1.490	0.136		
Proportion of shared ASVs in feces	S	Outdoor	37	(Intercept)	−2.865	1.325	−2.163	0.031	*	0
				Outdoor	−3.306	1.366	−2.420	0.016	*	
	S, A, and W	Handwashing × timepoint + number.of.persons × timepoint	73	(Intercept)	−8.086	1.985	−4.074	<0.001	***	1, 3
				HandwashingOFTEN	0.291	1.295	0.225	0.822		
				TimepointA	−4.849	2.198	−2.206	0.027	*	
				TimepointW	0.215	1.769	0.122	0.903		
				Number.of.persons	0.599	0.388	1.542	0.123		
				HandwashingOFTEN:timepointA	1.933	0.922	2.096	0.036	*	
				HandwashingOFTEN:timepointW	−1.308	0.656	−1.992	0.046	*	
				TimepointA:number.of.persons	1.081	0.520	2.077	0.038	*	
				TimepointW:number.of.persons	0.260	0.463	0.562	0.574		

^*a*^ Results of the four significant GLMM models that resulted from the forward selection. Timepoints are S, spring; A, autumn; and W, winter. *P* values: *, *P* < 0.05; **, *P* < 0.01; ***, *P* < 0.001. DHARMa was conducted to inspect model quality; see Materials and Methods for details. Numbers denote the following: (0) no warnings, (1) quantile deviations detected, (2) Kolmogorov–Smirnov test (KS test): deviation significant, (3) dispersion test: deviation significant, and (4) within-group deviations from uniformity significant. Variable “number.of.persons” refers to the number of residents/visitors during the time doormat was installed, and “real.mat.days” is the number of days the mat was effectively collecting the material.

**Fig 8:**
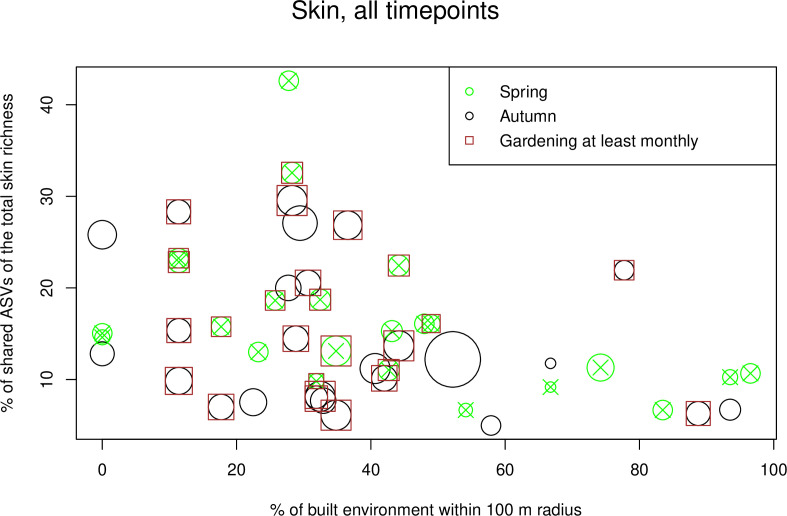
Scatter plot of most important variables in skin GLMMs. Percentage of shared ASVs in skin samples plotted against the coverage of built environment. The size of the bubble is related to the number of effective sampling days. An additional brown square denotes study subjects who did gardening at least monthly. All the other subjects did gardening more rarely. Note that for some study subjects, there are two observations in this graph, but for readability, they are not highlighted. In the GLMM models, study subject was included as a random effect.

**Fig 9:**
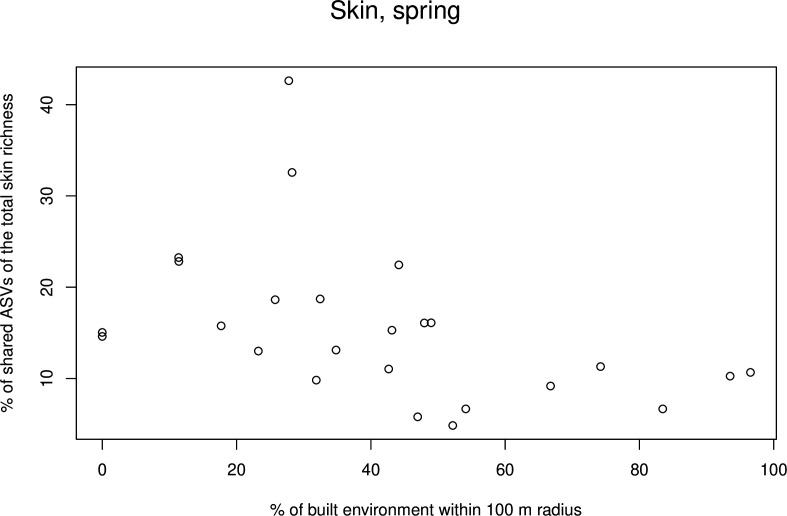
Scatter plot of the most important variable in skin GLMMs. The percentage of shared ASVs in skin samples in the spring data set is plotted against the coverage of built environment.

Contrary to our saliva data, initial models for skin data showed an increasing proportion of shared ASVs with decreasing built environment (estimate −0.011, *P* = 0.008; Table S1b). In skin data, three explanatory variables had interactions with timepoint (i.e., gardening, days that mat was in use, and handwashing). Also contrary to the saliva data, the number of persons living and visiting was positively associated with the proportion of shared ASVs (estimate 0.142, *P* = 0.041). Timepoint was also significant, and the proportion of shared ASVs was lower in autumn compared to spring (estimate −0.183, *P* = 0.005). Forward selection with skin data led to a complex model including two interactions with timepoint (days that mat was in use and gardening) and built environment (Table 2; Fig. 8). When timepoints were analyzed separately, no explanatory variables appeared informative in the autumn. In spring, built environment was the only explanatory variable entering the model (estimate −0.012, *P* = 0.005; Fig. 9).

**Fig 10:**
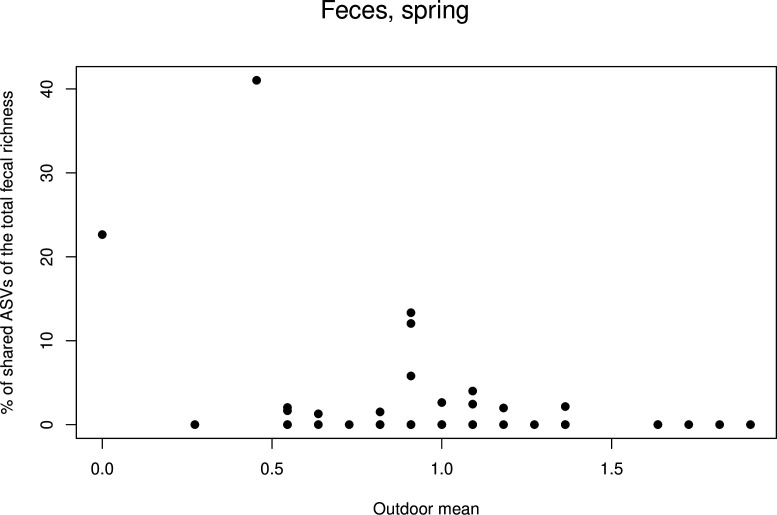
Scatter plot of the most important variables in fecal GLMMs. The percentage of shared ASVs in skin samples is plotted against the index representing the general amount of time the study subject spent outdoors. Note that the pattern is strongly driven by two exceptional observations.

In initial models for fecal data, significant interactions appeared for five explanatory variables (i.e., built environment, inside pets, gardening, number of persons living and visiting in the house, and handwashing; Table S1c). After forward selection, the model included two interactions with timepoint (handwashing and number of residents/visitors, Table 2). When the timepoints were analyzed separately, none of the explanatory variables remained informative in winter. Too few fecal observations were available in autumn data, which permitted the analysis of this timepoint (see Table 3). In spring, however, one explanatory variable entered the model: the general amount of outdoor recreation was negatively associated with the proportion of shared ASVs (estimate −3.306, *P* = 0.016). Further inspection of the scatter plot (Fig. 10) indicated that two observations strongly affected this result.

**TABLE 3:** Data characteristics^*a*^

	Saliva, spring		Saliva, autumn		Saliva, winter		Skin, spring		Skin, autumn		Feces, spring		Feces, autumn		Feces, winter	
*N*	37		30		37		25		27		37		16		23	
Female	15		12		15		12		12		16		4		8	
Male	22		18		22		13		15		21		12		15	
Cohort36–40	16		14		20		11		14		16		7		14	
Cohort46–50	21		16		17		14		13		21		9		9	
Hands.max.once.a.day	9	[1]	10	[1]	10	[1]	6	[1]	9		8	[1]	5	[1]	6	
Hands.many.times.a.day	27		19		26		18		18		28		10		17	
Gardening.rarely	18	[1]	13	[1]	18	[2]	13	[1]	11	[1]	18	[2]	10	[1]	12	[2]
Gardening.at.least.monthly	18		16		17		11		15		17		5		9	
Inside.pets.yes	5		6		5		4		7		6		2		2	
Inside.pets.no	32		24		32		21		20		31		14		21	
Birth year	1944 (1936–1950)		1943 (1936–1950)		1943 (1936–1950)		1944 (1936–1950)		1943 (1936–1950)		1944 (1936–1950)		1944 (1936–1950)		1942 (1936–1950)	
Built %	39 (0–97)		33 (0–94)		46 (0–100)		42 (0–97)		37 (0–94)		37 (0–97)		30 (0–89)		46 (0–98)	
Outdoor	1.02 (0–1.91)		1.05 (0.27–1.91)		1 (0–1.91)		1.02 (0.27–1.91)		1.08 (0–1.91)		0.99 (0–1.91)		0.89 (0–1.82)		0.88 (0–1.91)	
Number of persons (five classes)	3 (1–5)	[1]	4 (2–5)		3 (1–5)		3 (1–5)	[1]	4 (2–5)		3 (1–5)	[1]	3 (2–5)		3 (1–5)	
Real mat days	13 (3–20)	[1]	17 (7–37)		15 (6–28)		13 (7–20)	[1]	18 (7–37)		13 (7–20)	[1]	17 (7–23)		15 (6–18)	
Tot. richness human	130 (86–168)		137 (67–203)		142 (55–218)		292 (117–585)		228 (125–413)		117 (44–240)		142 (55–356)		129 (66–231)	
Tot. richness mat	632 (465–754)		705 (537–805)		589 (351–894)		709 (399–850)		672 (559–768)		641 (457–774)		652 (539–731)		466 (283–684)	
Shared richness	1 (0–6)		1 (0–6)		4 (0–18)		52 (8–228)		34 (0–122)		3 (0–48)		3 (0–20)		3 (0–16)	
% shared ASVs	0.8 (0–4)		0.8 (0–5)		3.4 (0–17)		15.5 (5–43)		13.9 (0–30)		3.1 (0–41)		3.1 (0–22)		3.5 (0–19)	

^*a*^ Data characteristics for independent and dependent variables. *N* refers to the number of study subjects that had both doormat and the given human sample type available. Cohort refers to birth year (either between 1936 and 1940 or between 1946 and 1950). For other variables, see Materials and Methods for the description. For class variables, the number of observations in each class is shown. For numerical variables, mean, minimum, and maximum values are given. The numbers of missing values are given in square brackets.

## DISCUSSION

Although our understanding of the positive effects of microbes on planetary and human health is improving, most research on microbial dispersal thus far has focused on negative health effects of microorganisms including transmission routes of human pathogens and of antimicrobial resistance genes (41). We explored if the proportion of ASVs shared between human and deposit samples could be used as a measure of dispersal. We then evaluated these data in the context of characteristics of the living environment and of the biodiversity hypothesis (4). We assumed that, in general, environmental microbial communities benefit human health.

Our first hypothesis that there are more shared bacteria when there is an indoor pet (dog or cat) in the household was not supported: none of the final models included indoor pets. Only for the initial models with saliva data was this variable significant, but—contrary to our hypothesis—the association was negative. Similarly, our second hypothesis that there are fewer shared bacteria for those who wash hands often was not supported. In the initial and final models, handwashing appeared only in interaction terms with timepoint, making its importance difficult to interpret.

We also hypothesized that outdoor recreation or gardening, the number of days the doormat has been in use, and the number of persons living and visiting the residence would have a positive relationship with the number of shared bacteria. Of these variables, outdoor recreation had a negative relationship with shared bacteria in saliva in winter and in feces in spring. The pattern for feces seemed to be strongly driven by two exceptional observations. Alas, more data are needed to confirm if this pattern is real. For saliva data, outdoor recreation had a negative association in winter. Results are further discussed in the following paragraphs.

For the degree of urbanization, we set no preassumptions. The coverage of built environment that was used as a proxy for urbanization appeared to have significant, but opposite, relationships with shared bacteria in saliva and skin data. When the amount of built environment increased, the proportion of shared ASVs increased in saliva but decreased in the skin. It is possible that there are more environmental bacteria on skin than in saliva, and simultaneously more human-originated bacteria on doormat in urban than in rural areas (see also 12). In other words, the increasing shared ASVs with built environment in our saliva data could be due to increasing proportion of human-associated bacteria in doormat in urban houses. Likewise, the decreasing shared ASVs with built environment in skin data could be due to increasing environmental bacteria on skin.

This could also explain why outdoor recreation was negatively associated with the proportion of shared ASVs in saliva data in winter: if ASVs shared between the mat and saliva samples are mainly of human origin, then outdoor recreation could increase bacteria of environmental origin on the mats and thus decrease the number of shared bacteria.

These observations point to a limitation of the doormat sampling as the mats accumulate bacteria of both environmental and human origin especially when the doormats are placed indoors as in the present study. These findings, however, also highlight the complexity of dispersal as a multidirectional phenomenon. Indeed, it is important to consider not only the dispersal volume but also its direction as well. To be able to estimate the origin of bacterial ASVs, especially in the mat samples, environmental bacterial samples from outdoors and deposit samples indoors may be informative.

Season also was important as a main effect as well as in several significant interactions with other variables, suggesting an important seasonal effect. In the saliva data, the proportion of shared ASVs was highest in the winter. This observation may be attributable to the winter activities, when people in Finland usually spend more time indoors, and more oral human microbiota deposit on to the mat. In spring, on the other hand, people often spend more time outdoors, thus increasing the transport of environmental bacteria onto doormats. In winter, the proportion of environmental bacteria on doormats may also decrease because of snow cover, which potentially decreases the transport of environmental bacteria indoors (31).

Based on previous studies showing frequent microbial oral-gut transmission (42, 43), it is somewhat surprising that our data showed only few shared ASVs between saliva and fecal samples (Fig. 4). Methodological differences may explain this result. For example, the selection of variable region (V4 vs V3–V4), use of taxonomic resolution (ASVs vs operational taxonomic units or genera), number of observations and timepoints as well as sequencing method (16S rDNA vs shot-gun sequencing) all affect the detection of shared taxa and complicate direct comparisons across studies. Importantly, our aim was not to identify which species are shared or to reveal the absolute number of shared ASVs. Instead, we searched for relationships between the proportion of shared ASVs and a group of explanatory variables that may affect the dispersal of bacteria from environment to human and vice versa.

Additionally, the taxonomic composition in this study seems different from other papers. For example, in our saliva data, phylum Fusobacteria dominated, although usually it does not (44). This may be attributable to our choice to remove all ASVs present in any of the negative controls. We made this choice to account for contamination during sampling (e.g., from the sampling personnel), in the laboratory (e.g., reagents or cross-contamination), or sequencing [“barcode hopping” (45)]. We are aware that this kind of contaminant removal may also remove ASVs representing the sample and is generally not recommended (45). However, such contamination could have introduced same contaminants to several sample types and thus could lead to false detection of shared bacteria. Hence, we selected this very rigorous way to deal with contamination. It is important to note that our primary research aim was not to identify which taxa are shared or to enumerate shared ASVs. Instead, we searched for relationships.

Today, microbial studies are often encouraged to reveal bacterial functions or activity by the use of metatranscriptomics, metaproteomics, and metabolomics (11). However, in the context of this paper, simple detection of DNA was justified. As the human immune system is also stimulated by bacterial structures, the stimulation need not rely on bacterial functioning or their metabolic activity; i.e., even inactive or dead bacteria can be important (46). Additionally, immune system stimulation may not require bacterial establishment, but temporary encounters also can be important.

Here we aimed to inspect bacterial dispersal at relatively large spatial scales that do not allow experimental manipulation of bacterial dispersal. A significant improvement to a study of this kind would be to include also purely environmental bacterial samples from outdoors and to deposit samples indoors to enable determination of the origin of the bacteria in both doormat and human samples. Moreover, using collectors installed on residents’ clothing could reveal missing links of bacterial dispersal from environment to humans. Another possible line of further research could be to split the bacterial community data into assemblages based on dispersal related traits such as dormancy or sporulation (19). Such approaches have been used with other organismal groups (47, 48) and could also be applied for human-associated bacteria.

### Conclusions

Our results suggest that studying bacterial taxa that are shared between human and the living environment is an approach that could be developed further for studying the drivers of bacterial dispersal. As skin and saliva bacterial communities had opposing relationships with the degree of urbanization, the results also suggest that different human bacterial communities may differ in their dispersal. This might be related to differing species pools in urban and rural environments. In the future, a similar approach aiming to study the bacterial dispersal at large scales should be accompanied with a more comprehensive sampling scheme, including also non-human sampling locations indoors and outdoors.

## MATERIALS AND METHODS

In this study, we used human and environmental bacterial samples collected from 53 elderly people (65–80 years) residing within the city of Lahti and surrounding rural municipalities in Southern Finland [see also reference (33)]. Participants were initially selected from a large prospective study called Good Aging in Lahti Region (49). Partly the same bacterial data have been used in previous studies (31, 33, 36, 50, 51). Of the participants whose samples were suitable for this study, 24 lived in the urban block of flats in the city of Lahti and 29 lived in rural areas in detached single-family houses outside densely populated communities.

For the original sample collection, we used several exclusion criteria when selecting participants. Thus, at the onset of the study, the participants did not suffer from any non-communicable chronic diseases affecting the immune response, including diabetes, chronic obstructive pulmonary disease, celiac disease, psoriasis requiring medication, dementia, multiple sclerosis, asthma with cortisone treatment, or cancer (active treatment during the last year or largely spread). We also excluded daily smokers and subjects on immunosuppressive or cortisone medications. Only observations preceded by at least 6 months without using any antibiotics were included in this study. Because of the aims of the original studies, the initial selection of the study subjects aimed to exclude pet owners in the urban area, although this criterion was later abandoned because of practical reasons. This, however, partly affected the sparse and unequal distribution of pet owners; i.e., there were less pet owners in urban than rural areas.

The table of data characteristics (Table 3) shows that the data sets included in this study consisted of slightly more males than females; this was especially true for fecal data. The number of study subjects in each of the data sets varied between 16 and 37. Many of the study subjects had gardening as a hobby (5–18 per data set doing gardening at least monthly), while much fewer had indoor pets (2–7 per data set). Percentage of built environment within a 100-m radius from the home varied in some data sets maximally (i.e., 0% and 100%) but at least between 0% and 89%.

### Sampling

Environmental bacteria deposited on doormats as well as human saliva and fecal samples were sampled in spring (June 2015), autumn (August 2015), and winter (February 2016). Skin swab samples were collected in spring (May 2015) and autumn (August 2015).

In spring, skin swabs were collected by a nurse who visited the participants in May 2015 (ca. 2 weeks before the collection of the other sample types in June). In August, skin swabs were taken by the participants following the instructions provided by the nurse. A sterile cotton swab was first soaked in solution containing 0.1% Tween 20 in 0.15-M NaCl in a sterile polyethene sample tube. Then an area of 2 × 2 cm in the middle of the forearm was carefully wiped, and the stick was cut and placed back to the sample tube with the solution. Skin swabs were either immediately placed in dry ice for transportation or first stored in a home freezer (−20°C) and then transported in dry ice. In the laboratory, samples were stored at −80°C until analyzed.

Saliva, feces, and environmental samples were collected in June and August 2015 and February 2016. Saliva samples were collected by the participants following detailed instructions. Samples were collected in the morning prior to eating, drinking, or brushing teeth. Three saliva swabs were collected. Each swab was placed under the tongue for 40 seconds, after which it was twiddled in the mouth and placed again under the tongue until the swab was saturated (total sample time for each swab ca. 1 min). Participants were instructed to take fecal samples with a sampling kit including a clean disposable cardboard plate and polyethylene fecal sample collection tubes. Immediately after taking saliva and fecal samples, the participants stored the samples in the household freezer (usually −20°C). A few days later, the study personnel transported the samples in dry ice to be stored at −80°C until analyzed.

When retrieving the human samples, the study personnel simultaneously collected the microbial samples from doormats [see also reference (33)]. Similar scraper plastic doormats (surface area 45 × 57 cm) were placed indoors immediately at the main entrance door of the study participant’s home for approximately 2 weeks. When collecting the samples from the doormats, any large organic matter (e.g., leaves and twigs) was first collected by hand using clean disposable gloves. The doormat was then turned upside down on a clean aluminum foil and tapped all over for about 10 seconds. The material on the foil was then transferred into a clean zipper plastic bag. The bag was sealed airtight and frozen immediately in dry ice for transportation and then stored at −80°C until analyzed.

### DNA extraction, amplification, and sequencing

Bacterial communities were analyzed using amplicon sequences of the hypervariable V4 region within the 16S rDNA sequenced by Illumina MiSeq (2 × 300 bp, v.3 reagent kit; Illumina, San Diego, CA, USA). From saliva samples, DNA was extracted using PowerSoil DNA Isolation Kit (MoBio Laboratories, Inc., Carlsbad, CA, USA), amplified using two-step PCR protocol (33) in CD Genomics (Shirley, NY, USA) and sequenced by the Integrated Genomics Facility at Kansas State University (Kansas, USA). Skin swab samples were extracted using Fast DNA spin kit for soil (MP Biomedicals, Santa Ana, CA, USA), amplified using two-step PCR protocol (52) in Environmental Laboratory (Lahti, Finland), and sequenced in the Institute for Molecular Medicine Finland FIMM (Helsinki, Finland). For fecal samples, 30–60 mg of frozen and unprocessed feces was used for DNA extraction with PowerSoil DNA Isolation Kit (MoBio Laboratories, Inc.) and amplified with one-step PCR protocol. Fecal samples were processed and sequenced in Charles University, Prague, Czech Republic using one-step PCR protocol (53). For doormat samples, at maximum, three replicates of 0.25 g of doormat debris were used for DNA extraction with PowerSoil DNA Isolation Kit (MoBio Laboratories) and amplified with two-step PCR protocol in Environmental Laboratory [see details from reference (33)]. Doormat samples were sequenced by the Integrated Genomics Facility at Kansas State University. Slightly varying protocols were justified based on experiences on different sample types and the large number of samples inefficient to be processed in a single laboratory. It is noteworthy that protocols do not differ within a sample type and thus do not interfere with the variation within each sample type. Altogether, 26 negative controls from extraction and PCR were also sequenced, but the number and type of controls slightly varied between sequencing batches.

### Bioinformatics

Paired-end sequence data (.fastq) from the rRNA gene data set of bacterial communities were processed using Mothur [v.1.43.0 and 1.44.1 (54)] mainly following previously published protocols (53, 55). Sequences for each sample type were first aligned into contigs after which files were merged and further processed simultaneously in one bioinformatic session. Sequences were screened to remove any sequences longer than 360 bp, with ambiguous bases or homopolymers larger than 8 bp long.

Sequences were aligned using Mothur version of SILVA bacterial reference [v.102 (56)]. Sequences were screened and filtered to start and end at the same place, which simultaneously removed primer sequences from those samples that were processed with two-step PCR (i.e., doormat, saliva, and skin) because fecal sample sequences did not include primer parts as they were processed with one-step PCR. Almost identical sequences (>99% similar) were preclustered to ASVs (57) and screened for chimeras with UCHIME (58), which uses the abundant sequences as a reference. The chimeric sequences were removed. Sequences were classified using the Mothur version of Bayesian classifier (59) with the RDP training set v.16 (60). Sequences classified to chloroplast, mitochondria, unknown, Archaea, and Eukaryota were removed from the analyses. ASVs represented with one in the whole data were considered as sequencing errors and were removed (61). Finally, all ASVs that were present in any of the negative controls were removed from the data. ASV level was selected because we were especially interested in bacterial fauna that is shared between doormat samples and human samples, and as the ASV level allows a difference of only 2 bp, it most probably reveals if the same bacterial species exists. However, it should be noted that when using only one variable region of 16S gene, it is possible that some of the shared ASVs do not belong to the same bacterial species.

### Numerical analyses

Data were first visualized using KRONA (40). All the other analyses and figures were done using RStudio [v.2022.07.2 (62)] and R [v.4.0.0 and 4.1.3 (63)]. The number of shared ASVs in each sample type was visualized using Venn diagrams [package VennDiagram (64)].

ASV data were subsampled to account for varying library sizes. Subsampling was conducted within each sample type, and the level of subsampling was selected as the lowest number of sequences in each sample type (range 1,002–1,208).

We used principal coordinate analysis (PCoA) to visualize communities simultaneously among all sample types. PCoA was run using function *cmdscale* in package *stats*. Permutational multivariate analysis of variance (PERMANOVA) (65) was run using package *vegan* (66) and pairwise contrasts using package *RVAideMemoire*, 67). Permutational test of multivariate homogeneity of group dispersions (PERMDISP, 68) was run using package *vegan*. PERMANOVA and PERMDISP were run corresponding to each PCoA figure using 999 permutations and using three different distance measures (functions *vegdist* and *decostand* in package *vegan*): Bray-Curtis distance, Sørensen (i.e., Bray-Curtis distance for presence-absence data), and Hellinger (i.e., Euclidean distance for Hellinger transformed data; 69).

### Generalized linear mixed-effects models

We used *lme4* (70) to perform a generalized linear mixed-effects model (GLMM) for the relative number of shared ASVs. Because the total amount of ASVs is likely to affect the number of shared ASVs, we counted the proportion of shared ASVs of the total number of ASVs found in a given human sample. We used binomial family in GLMM because of the proportional nature of the dependent variable (71). Timepoint was always included in the models as a fixed effect and person ID as a random effect. If the initial model did not converge, we used an optimizer (“bobyqa”) to solve the issue.

Forward selection was used to find final models. First, explanatory variables were analyzed one by one to check if there was an interaction between the given explanatory variable and timepoint (see flowchart in Fig. S1). If the interaction was absent, a new model without interaction was built. These models were used to select the first variable with the lowest (and below 0.05) *P* value. Next, each of the remaining explanatory variables and significant interactions was included one by one, and each candidate model was compared to the preceding model using the function *anova*(). The second variable entered to the model was that with the lowest Akaike Information Criterion value (AIC). This procedure was carried on until the new variables no longer made the model better. To avoid too complex models, we used three as a threshold; i.e., if the AIC for the more parsimonious model was less than three smaller compared to the AIC for the more complex model, we stopped the selection procedure and selected the parsimonious model. Because there were also some significant interactions, we ran a similar procedure for each timepoint separately. There were some observations missing for some of the variables. For the first step of variable selection, we removed only the rows with missing data for a given variable. For the next steps of the variable selection, all the rows with missing information were removed. Finally, we reran the final models using data where only the rows with missing information in the selected variables were removed.

There were altogether seven explanatory variables that were used to build the final models. Percentage of built environment (including hardscapes) within a 100-m radius from the study subject’s home (variable name “built”) was estimated using the CORINE Land Cover 2012 database. All the other variables were based on questionnaires filled by the study subjects. There were binomial variables indicating the presence/absence of a dog or cat living inside the home (“pets”), if the study subject washed his/her hands at maximum once a day or several times a day (“handwashing”), and if the study subject did gardening at least monthly or less often (“gardening”). The following variables were treated as continuous variables. The mean value for outdoor recreation (“outdoor”) was counted from altogether 11 outdoor activities (walking, cycling, hiking, berry picking, mushroom picking, hunting, fishing, birdwatching, horse riding, gardening, and other outdoor recreation) that had been reported using a Likert scale ranging from 1 (“never”) to 5 (“daily”), although the maximum value reported was 4 (“weekly”). The number of days the doormat was effectively in use (“real.mat.days”) was counted by subtracting the number of days there was nobody home from the number of days the doormat was in use. The number of persons living or visiting the household (“number.of.persons”) during the use of the doormat were collected as a categorical variable (1–10, >10), but it was coded as a continuous numerical variable with values from 1 to 5 to simplify the model interpretation. Residuals of the GLMM models were inspected using package DHARMa (72). Some models showed significant quantile deviations or overdispersion/underdispersion. However, based on a visual inspection, we evaluated the models as acceptable (Fig. S4 to S8), given that the main purpose of this study is rather explore the possible correlations with shared ASVs than making predictions.

In the early stage of this study, we conducted the analyses with the complete data set, i.e., including also those observations where the study subject had used antibiotics in last 6 months prior to sampling. There were 10, 8, and 9 such observations in saliva, skin and fecal data, respectively. Possibly due to very scattered data, together with the presumably strong effect of antibiotics, the models did not work well (data not shown). Thus, we decided to remove these observations. All the numbers of samples and study participants reported earlier in this paper refer to the final data set used in the analyses.

## Data Availability

Raw sequence reads are available in the NCBI Sequence Read Archive under BioProject number PRJNA1114390.

## SUPPLEMENTAL MATERIAL

Appendix A: Interactive Krona figure aem.00903-24-s0001.html Interactive HTML file showing relative abundances of taxa in spring data at all taxonomic levels available (up to ASV level).

Supplemental material aem.00903-24-s0002.pdf Figures S1 to S8; Tables S1a to S1c.

ASM does not own the copyrights to Supplemental Material that may be linked to, or accessed through, an article. The authors have granted ASM a non-exclusive, world-wide license to publish the Supplemental Material files. Please contact the corresponding author directly for reuse.
