# Supplementary material for: Using patterns of shared taxa to infer bacterial dispersal in human living environment in urban and rural areas
Source: Appl Environ Microbiol. 2024 Sep 4;90(10):e00903-24. doi: 10.1128/aem.00903-24 (PMC11498140; doi:10.1128/aem.00903-24)
Supplement: Appendix A: Interactive Krona figure — Interactive HTML file showing relative abundances of taxa in spring data at all taxonomic levels available (up to ASV level). [file aem.00903-24-s0001.html]

Javascript must be enabled to view this page.

magnitude

mat
saliva
skin
stool

 19551
 19551
 19551
 19551

 1017
 1
 315

 290
 1
 116

 2
 1

 2
 1

 2
 1

 1

 1

 0
 1

 70
 0
 41

 70
 0
 41

 70
 0
 41

 1

 1

 1

 1

 1

 1

 1

 1

 2

 2

 1

 2
 0
 1

 1

 1
 0
 2

 5
 0
 10

 1

 1
 0
 2

 1

 3

 12

 16
 0
 6

 2

 2

 1

 1

 1

 1

 1

 1

 1

 1
 0
 1

 1

 1

 0
 0
 1

 0
 0
 2

 0
 0
 1

 0
 0
 1

 0
 0
 1

 0
 0
 1

 0
 0
 2

 0
 0
 1

 0
 0
 1

 0
 0
 1

 0
 0
 1

 0
 0
 3

 0
 0
 1

 0
 0
 1

 0
 0
 1

 3
 0
 1

 3
 0
 1

 3
 0
 1

 2

 1

 0
 0
 1

 16
 0
 25

 16
 0
 25

 16
 0
 25

 1

 1

 1

 1

 1
 0
 1

 1

 1

 1

 2

 1

 1

 1

 1

 1
 0
 1

 1

 0
 0
 1

 0
 0
 2

 0
 0
 1

 0
 0
 1

 0
 0
 1

 0
 0
 2

 0
 0
 1

 0
 0
 1

 0
 0
 1

 0
 0
 1

 0
 0
 1

 0
 0
 4

 0
 0
 1

 0
 0
 1

 0
 0
 1

 0
 0
 1

 0
 0
 1

 0
 0
 1

 147
 0
 37

 147
 0
 37

 147
 0
 37

 1

 1

 2

 3

 2

 2
 0
 1

 1

 1

 6

 9

 1

 3
 0
 1

 2

 2

 1
 0
 4

 2

 1

 1

 2

 4

 3

 1

 4

 2

 1

 2
 0
 3

 1

 1
 0
 1

 4

 1

 17
 0
 2

 14

 3

 2

 1

 3
 0
 3

 8
 0
 1

 3
 0
 2

 1

 1

 5
 0
 4

 4

 1

 2

 2

 2

 1

 1

 3

 3

 1

 1

 1

 0
 0
 1

 0
 0
 9

 0
 0
 1

 0
 0
 4

 2
 0
 1

 2
 0
 1

 2
 0
 1

 1

 1

 0
 0
 1

 50
 0
 11

 50
 0
 11

 50
 0
 11

 1

 1

 1

 1

 1

 8

 16
 0
 2

 4
 0
 5

 15
 0
 2

 2

 0
 0
 1

 0
 0
 1

 16
 0
 7

 16
 0
 7

 16
 0
 7

 16
 0
 7

 1

 1

 1
 0
 2

 2
 0
 1

 1

 2
 0
 2

 4

 2

 1

 1

 0
 0
 1

 0
 0
 1

 68
 0
 35

 68
 0
 35

 68
 0
 35

 68
 0
 35

 1

 1

 1

 1

 1

 1

 1

 1

 1

 1

 1

 2

 2

 1

 3
 0
 4

 4

 5

 7

 1

 1
 0
 5

 13
 0
 7

 2

 2

 1

 1

 3
 0
 2

 1

 1
 0
 2

 1

 1

 1

 1

 1

 1

 1

 0
 0
 1

 0
 0
 1

 0
 0
 1

 0
 0
 1

 0
 0
 1

 0
 0
 2

 0
 0
 1

 0
 0
 1

 0
 0
 2

 0
 0
 1

 0
 0
 1

 0
 0
 1

 0
 0
 1

 6
 0
 8

 6
 0
 8

 6
 0
 8

 6
 0
 8

 1

 1

 1

 2

 1

 0
 0
 3

 0
 0
 1

 0
 0
 1

 0
 0
 1

 0
 0
 1

 0
 0
 1

 34
 0
 26

 34
 0
 26

 34
 0
 26

 34
 0
 26

 1

 1

 1

 1
 0
 2

 1

 1

 1

 1

 1

 2

 1

 7

 1

 1

 2

 2

 1

 1

 2

 1

 1

 2

 1
 0
 2

 0
 0
 1

 0
 0
 3

 0
 0
 1

 0
 0
 5

 0
 0
 1

 0
 0
 1

 0
 0
 1

 0
 0
 2

 0
 0
 2

 0
 0
 1

 0
 0
 1

 0
 0
 1

 0
 0
 2

 1
 0
 1

 1
 0
 1

 1
 0
 1

 1
 0
 1

 1

 0
 0
 1

 195
 0
 37

 86
 0
 11

 86
 0
 11

 86
 0
 11

 1

 1

 1

 1

 1

 1

 1

 1

 1

 1

 1

 2

 2

 3

 5

 1

 8

 1

 1

 1

 2

 2

 1

 1

 3

 2

 4
 0
 1

 1

 4
 0
 1

 1

 1

 7

 1

 1

 1

 1

 1

 2

 1

 2

 4

 1

 1

 1

 1

 1

 1

 1

 1

 0
 0
 1

 0
 0
 1

 0
 0
 2

 0
 0
 1

 0
 0
 1

 0
 0
 2

 0
 0
 1

 1

 1

 1

 1

 7
 0
 4

 7
 0
 4

 7
 0
 4

 1

 1

 1

 1

 2

 1

 0
 0
 1

 0
 0
 1

 0
 0
 2

 99
 0
 20

 99
 0
 20

 99
 0
 20

 1

 1

 1

 1

 1

 1

 1

 1

 1

 2

 1

 1

 1

 1

 1

 2

 1

 1

 1

 2

 1

 1

 2

 7

 1

 1

 2

 1

 1

 1

 1

 3

 1

 3

 4

 1

 1

 6

 3
 0
 1

 1

 3

 1
 0
 1

 3

 2

 1

 1

 1

 3

 1

 1
 0
 1

 1

 2
 0
 1

 1

 3
 0
 2

 1

 1

 2

 1
 0
 1

 1

 1

 1

 1

 1

 0
 0
 1

 0
 0
 2

 0
 0
 1

 0
 0
 1

 0
 0
 1

 0
 0
 1

 0
 0
 1

 0
 0
 1

 0
 0
 1

 0
 0
 1

 0
 0
 1

 0
 0
 1

 2
 0
 2

 2
 0
 2

 2
 0
 2

 2
 0
 2

 303
 0
 26

 180
 0
 15

 180
 0
 15

 180
 0
 15

 1

 1

 1

 2

 1

 1

 1

 1

 3

 2

 1

 6

 2

 1

 1

 1

 1

 2

 1

 4

 2

 3
 0
 1

 3

 9

 39
 0
 1

 1

 2

 33
 0
 8

 5

 1

 1

 2

 1

 1

 1

 1

 1

 23

 1

 3

 1

 1

 1

 2

 1

 1

 2

 1

 1

 1

 1

 0
 0
 1

 0
 0
 2

 0
 0
 1

 0
 0
 1

 51
 0
 8

 51
 0
 8

 51
 0
 8

 1

 1

 2

 1

 1

 2

 12
 0
 2

 5

 2

 11
 0
 2

 2

 3
 0
 1

 3

 3

 1

 1

 0
 0
 1

 0
 0
 2

 57

 57

 57

 1

 1

 1

 1

 1

 23

 7

 10

 2

 3

 5

 1

 1

 15
 0
 3

 15
 0
 3

 15
 0
 3

 1

 1

 1

 2

 1

 2

 1

 1

 1

 1

 1

 1

 1

 0
 0
 1

 0
 0
 1

 0
 0
 1

 1
 0
 1

 1
 0
 1

 1
 0
 1

 1
 0
 1

 1

 0
 0
 1

 95
 0
 47

 95
 0
 47

 95
 0
 47

 95
 0
 47

 1

 1

 1

 2

 1

 1

 1

 1

 1

 1

 1

 1

 1

 1

 3
 0
 2

 1

 2
 0
 1

 3

 3

 1

 1

 1

 1

 4

 1

 5

 1

 1

 2
 0
 6

 1

 2
 0
 1

 1

 4
 0
 3

 1

 1

 1

 1
 0
 1

 2

 6

 1
 0
 2

 1

 1

 1

 1

 1

 2

 1

 1

 1
 0
 1

 1

 1

 1

 1

 1

 1

 1

 1

 1

 1

 1

 1

 1

 2

 2

 1

 1

 0
 0
 2

 0
 0
 1

 0
 0
 1

 0
 0
 1

 0
 0
 1

 0
 0
 2

 0
 0
 2

 0
 0
 1

 0
 0
 1

 0
 0
 1

 0
 0
 2

 0
 0
 1

 0
 0
 1

 0
 0
 1

 0
 0
 1

 0
 0
 1

 0
 0
 2

 0
 0
 1

 0
 0
 1

 0
 0
 1

 0
 0
 1

 0
 0
 2

 0
 0
 1

 0
 0
 1

 7
 0
 10

 7
 0
 10

 7
 0
 10

 7
 0
 10

 1

 1

 1
 0
 3

 1

 1

 1

 1

 0
 0
 1

 0
 0
 1

 0
 0
 2

 0
 0
 1

 0
 0
 2

 1

 1

 1

 1

 1

 0
 0
 1

 0
 0
 1

 0
 0
 1

 0
 0
 1

 0
 0
 1

 3076
 832
 6901
 697

 2995
 832
 6881
 686

 131
 0
 41

 5
 0
 6

 5
 0
 6

 1

 1

 1

 1
 0
 2

 1

 0
 0
 3

 0
 0
 1

 79
 0
 19

 79
 0
 19

 1

 2

 1

 1

 1

 3

 1

 3

 1

 3

 9
 0
 2

 3

 1

 1

 8
 0
 2

 1

 2
 0
 1

 2

 6

 1

 3

 3

 1

 1

 1

 1

 1

 2

 1

 1

 1

 1

 2

 2

 2

 1

 1

 1

 1

 1

 0
 0
 1

 0
 0
 1

 0
 0
 1

 0
 0
 1

 0
 0
 1

 0
 0
 1

 0
 0
 1

 0
 0
 1

 0
 0
 1

 0
 0
 1

 0
 0
 1

 0
 0
 1

 0
 0
 1

 0
 0
 1

 2

 2

 2

 45
 0
 16

 19
 0
 7

 1

 1

 1

 1
 0
 1

 7
 0
 1

 2

 3

 1
 0
 1

 1

 1

 0
 0
 4

 1
 0
 3

 1

 0
 0
 3

 25
 0
 6

 1

 1

 1

 1

 2

 11
 0
 2

 1

 1

 1

 5

 0
 0
 1

 0
 0
 1

 0
 0
 1

 0
 0
 1

 114
 0
 32

 114
 0
 32

 114
 0
 32

 1

 1

 1

 1

 1

 2

 1

 1

 1

 1
 0
 1

 1

 2

 1

 1

 2

 4

 5

 1

 1

 1

 3

 6

 1

 1

 2

 1

 2

 1

 15
 0
 2

 4
 0
 1

 7

 1

 1

 1

 3

 2

 2

 1

 1

 1

 3

 1

 2
 0
 2

 2
 0
 1

 1

 1

 2

 1

 1

 1

 1

 1

 1
 0
 1

 1

 1

 1

 1

 1

 1

 1

 1

 1

 1

 0
 0
 1

 0
 0
 1

 0
 0
 1

 0
 0
 1

 0
 0
 3

 0
 0
 1

 0
 0
 1

 0
 0
 1

 0
 0
 1

 0
 0
 1

 0
 0
 2

 0
 0
 2

 0
 0
 1

 0
 0
 3

 0
 0
 1

 0
 0
 2

 0
 0
 1

 2434
 643
 6462
 2

 6
 114
 305
 2

 2
 113
 245
 2

 1
 0
 0
 1

 1

 0
 1

 0
 1

 0
 2

 0
 1

 0
 3

 0
 2

 0
 1

 0
 2

 0
 1

 0
 1

 0
 1

 0
 3

 0
 1

 0
 1

 0
 1

 0
 2

 0
 3

 0
 30
 37

 0
 11
 55

 0
 2

 0
 3
 11

 0
 2
 8

 0
 4
 2

 0
 9
 12

 0
 3

 0
 3

 0
 7

 0
 1

 0
 1

 0
 2

 0
 2
 4
 1

 0
 1

 0
 2

 0
 1

 0
 1

 0
 1

 0
 0
 1

 0
 0
 4

 0
 0
 8

 0
 0
 77

 0
 0
 3

 0
 0
 14

 0
 0
 7

 0
 0
 1

 0
 0
 1

 2

 2

 2

 1

 1

 0
 1
 2

 0
 1

 0
 0
 2

 0
 0
 3

 0
 0
 3

 0
 0
 13

 0
 0
 11

 0
 0
 1

 0
 0
 1

 0
 0
 3

 0
 0
 3

 0
 0
 39

 0
 0
 1

 0
 0
 34

 0
 0
 3

 0
 0
 1

 324
 5
 101

 324
 5
 101

 1

 1

 1

 1

 1

 1

 1

 1

 1

 1

 1

 1

 1

 1

 1

 1

 1

 1

 1

 1

 2

 1

 1

 1

 1

 1

 1

 1

 1

 1

 1

 2

 1

 1

 1

 2

 1

 3

 1

 1

 1

 3

 1

 2
 0
 2

 1

 1

 2

 4
 0
 3

 5

 9
 0
 4

 1

 3

 3
 0
 1

 2
 0
 3

 1

 1

 4

 1

 1

 6
 0
 1

 1

 1
 0
 1

 1

 1

 1

 3

 2

 1

 13

 1

 21
 0
 1

 1

 4

 13

 1

 4

 12

 1

 2

 11

 5
 0
 2

 3

 1

 14
 0
 1

 1

 6

 1
 0
 1

 1

 1

 2

 1

 6

 2

 3

 1

 6

 1

 3

 4

 1
 0
 1

 1

 1

 1

 1
 0
 2

 3

 1

 5

 3

 1

 2
 0
 1

 1

 1

 1

 1

 2

 1

 1

 4

 1

 3

 1

 1

 4

 1

 1

 2

 1
 0
 1

 1
 0
 1

 1

 1

 1

 1

 1

 1

 1

 1

 1

 1

 1

 1

 1

 1

 1

 1

 1

 1

 1

 1

 1

 1

 0
 1

 0
 1

 0
 3

 0
 0
 2

 0
 0
 1

 0
 0
 1

 0
 0
 1

 0
 0
 2

 0
 0
 1

 0
 0
 1

 0
 0
 1

 0
 0
 1

 0
 0
 1

 0
 0
 3

 0
 0
 1

 0
 0
 1

 0
 0
 1

 0
 0
 3

 0
 0
 1

 0
 0
 1

 0
 0
 1

 0
 0
 1

 0
 0
 2

 0
 0
 1

 0
 0
 1

 0
 0
 3

 0
 0
 1

 0
 0
 1

 0
 0
 1

 0
 0
 2

 0
 0
 1

 0
 0
 1

 0
 0
 1

 0
 0
 1

 0
 0
 1

 0
 0
 1

 0
 0
 1

 0
 0
 5

 0
 0
 5

 0
 0
 1

 0
 0
 1

 0
 0
 2

 0
 0
 1

 0
 0
 1

 0
 0
 2

 0
 0
 1

 0
 0
 1

 0
 0
 1

 0
 0
 2

 0
 0
 1

 0
 0
 1

 0
 0
 1

 0
 0
 1

 0
 0
 1

 0
 0
 1

 0
 0
 2

 1
 0
 1

 1
 0
 1

 1

 0
 0
 1

 22
 0
 118

 21
 0
 118

 1

 2
 0
 33

 18
 0
 58

 0
 0
 2

 0
 0
 14

 0
 0
 2

 0
 0
 7

 0
 0
 1

 0
 0
 1

 1

 1

 8
 0
 8

 8
 0
 7

 1

 6
 0
 1

 1

 0
 0
 1

 0
 0
 3

 0
 0
 1

 0
 0
 1

 0
 0
 1

 0
 0
 1

 130
 160
 3566

 1
 1

 1

 0
 1

 127
 159
 3465

 1
 1

 1

 1

 1

 1

 4
 0
 3

 1

 1

 2

 1

 1
 0
 4

 2
 0
 6

 2

 7
 0
 269

 1
 0
 788

 6
 0
 171

 5
 0
 9

 2
 0
 1

 5
 0
 809

 14
 0
 13

 1
 114
 176

 1
 0
 46

 1

 23
 0
 27

 8
 0
 1

 5

 24

 2
 0
 7

 1

 2

 0
 1

 0
 1

 0
 1

 0
 1

 0
 2

 0
 4

 0
 33

 0
 1

 0
 0
 1

 0
 0
 1

 0
 0
 2

 0
 0
 1

 0
 0
 2

 0
 0
 1

 0
 0
 3

 0
 0
 2

 0
 0
 1

 0
 0
 1

 0
 0
 1

 0
 0
 2

 0
 0
 1

 0
 0
 3

 0
 0
 1

 0
 0
 1

 0
 0
 4

 0
 0
 1

 0
 0
 1

 0
 0
 1

 0
 0
 1

 0
 0
 1

 0
 0
 3

 0
 0
 1

 0
 0
 3

 0
 0
 1

 0
 0
 10

 0
 0
 11

 0
 0
 2

 0
 0
 30

 0
 0
 10

 0
 0
 6

 0
 0
 1

 0
 0
 3

 0
 0
 1

 0
 0
 40

 0
 0
 4

 0
 0
 3

 0
 0
 3

 0
 0
 143

 0
 0
 31

 0
 0
 1

 0
 0
 1

 0
 0
 1

 0
 0
 117

 0
 0
 11

 0
 0
 255

 0
 0
 9

 0
 0
 3

 0
 0
 2

 0
 0
 37

 0
 0
 188

 0
 0
 60

 0
 0
 16

 0
 0
 24

 0
 0
 7

 0
 0
 2

 0
 0
 6

 0
 0
 10

 0
 0
 1

 0
 0
 1

 0
 0
 1

 0
 0
 2

 0
 0
 4

 0
 0
 1

 0
 0
 1

 0
 0
 1

 0
 0
 1

 0
 0
 1

 0
 0
 2

 0
 0
 1

 0
 0
 1

 0
 0
 12

 0
 0
 3

 0
 0
 1

 0
 0
 1

 0
 0
 1

 0
 0
 1

 0
 0
 1

 0
 0
 1

 0
 0
 2

 0
 0
 1

 0
 0
 1

 0
 0
 1

 0
 0
 2

 2
 0
 101

 1
 0
 69

 1

 0
 0
 32

 54
 0
 12

 1

 1

 53
 0
 12

 1

 1

 1

 2
 0
 1

 6

 28
 0
 1

 4

 1

 3

 1
 0
 1

 1
 0
 1

 1
 0
 1

 1

 1

 1

 0
 0
 1

 0
 0
 1

 0
 0
 1

 0
 0
 1

 0
 0
 2

 0
 0
 1

 9

 9

 6

 3

 51
 0
 250

 47
 0
 113

 1

 1

 1
 0
 4

 2
 0
 2

 1
 0
 56

 32
 0
 11

 3
 0
 16

 2

 1

 1

 1

 1

 0
 0
 1

 0
 0
 1

 0
 0
 5

 0
 0
 17

 3
 0
 132

 3
 0
 103

 0
 0
 29

 1
 0
 5

 1

 0
 0
 2

 0
 0
 3

 8
 0
 45

 2
 0
 2

 2
 0
 2

 5
 0
 30

 2
 0
 2

 3
 0
 3

 0
 0
 3

 0
 0
 22

 1
 0
 13

 1

 0
 0
 6

 0
 0
 7

 4
 0
 9

 4
 0
 9

 3
 0
 5

 1

 0
 0
 3

 0
 0
 1

 57
 0
 12

 37
 0
 8

 1

 1

 34
 0
 4

 1

 0
 0
 4

 4
 0
 1

 2

 1

 1

 0
 0
 1

 2
 0
 2

 1

 1

 0
 0
 1

 0
 0
 1

 14
 0
 1

 1

 4

 2

 1

 1

 1

 1

 1

 1

 1

 0
 0
 1

 214
 0
 102

 1

 1

 21
 0
 2

 1

 1

 1

 10

 8
 0
 1

 0
 0
 1

 119
 0
 57

 1

 1

 1

 1

 1

 1

 1

 1

 1

 2

 2
 0
 2

 8

 2

 1

 6
 0
 3

 5
 0
 1

 1

 2

 16
 0
 1

 38
 0
 1

 1

 1
 0
 1

 2

 2
 0
 3

 1

 1

 4

 3

 1

 3
 0
 2

 2

 1

 1

 1

 1

 1

 1

 0
 0
 1

 0
 0
 1

 0
 0
 23

 0
 0
 2

 0
 0
 2

 0
 0
 5

 0
 0
 5

 0
 0
 3

 0
 0
 1

 7
 0
 2

 7
 0
 2

 1
 0
 5

 1

 0
 0
 2

 0
 0
 3

 1
 0
 1

 1
 0
 1

 4
 0
 10

 1

 1
 0
 4

 1
 0
 3

 1

 0
 0
 1

 0
 0
 1

 0
 0
 1

 6
 0
 3

 5
 0
 3

 1

 33
 0
 9

 1

 1

 29
 0
 9

 1

 1

 20
 0
 13

 19
 0
 12

 1
 0
 1

 1

 1

 1

 1

 1

 1

 1

 1

 52
 0
 6

 10

 1

 4

 5

 6
 0
 3

 1

 5
 0
 2

 0
 0
 1

 23
 0
 1

 19

 2

 2
 0
 1

 13
 0
 2

 1

 1

 1

 1

 3

 4

 1

 1

 0
 0
 2

 205
 0
 244

 1

 1

 1
 0
 1

 1

 0
 0
 1

 52
 0
 7

 51
 0
 7

 1

 1

 1

 1
 0
 1

 1

 0
 0
 1

 1

 1

 15
 0
 17

 11
 0
 3

 1
 0
 2

 2
 0
 3

 1

 0
 0
 4

 0
 0
 4

 0
 0
 1

 127
 0
 100

 1

 1

 1

 1

 1

 1

 1

 1

 1

 1

 1

 1

 1

 1

 1

 1

 1

 1

 3
 0
 1

 3

 1

 1

 5

 16
 0
 8

 17
 0
 17

 23
 0
 3

 4
 0
 11

 1
 0
 1

 9
 0
 12

 4

 2

 2

 2

 1

 1

 3
 0
 2

 1

 1
 0
 1

 1
 0
 5

 1

 1

 1

 1

 1

 1

 1

 1

 0
 0
 1

 0
 0
 1

 0
 0
 1

 0
 0
 1

 0
 0
 1

 0
 0
 1

 0
 0
 1

 0
 0
 1

 0
 0
 4

 0
 0
 1

 0
 0
 1

 0
 0
 6

 0
 0
 1

 0
 0
 4

 0
 0
 7

 0
 0
 1

 0
 0
 2

 0
 0
 1

 0
 0
 2

 0
 0
 1

 2
 0
 107

 1
 0
 15

 1
 0
 7

 0
 0
 3

 0
 0
 5

 0
 0
 2

 0
 0
 3

 0
 0
 1

 0
 0
 1

 0
 0
 46

 0
 0
 3

 0
 0
 1

 0
 0
 1

 0
 0
 4

 0
 0
 15

 4
 0
 6

 4
 0
 6

 0
 0
 5

 0
 0
 3

 0
 0
 2

 207
 301
 945

 103
 0
 49

 1

 1

 1

 1

 1

 25
 0
 1

 1
 0
 21

 2
 0
 13

 3
 0
 1

 1

 58
 0
 9

 1

 2

 1

 1

 1

 1

 1

 0
 0
 1

 0
 0
 1

 0
 0
 2

 1
 0
 2

 1

 0
 0
 2

 37
 0
 281

 1

 8
 0
 111

 22
 0
 103

 5
 0
 37

 1

 0
 0
 1

 0
 0
 1

 0
 0
 16

 0
 0
 3

 0
 0
 4

 0
 0
 1

 0
 0
 1

 0
 0
 3

 52
 0
 213

 1

 1

 1

 6
 0
 2

 1

 2

 1

 1
 0
 24

 1
 0
 2

 27

 1

 1

 1
 0
 1

 1

 1

 1

 1

 1

 1

 1

 0
 0
 7

 0
 0
 1

 0
 0
 1

 0
 0
 1

 0
 0
 1

 0
 0
 1

 0
 0
 1

 0
 0
 1

 0
 0
 72

 0
 0
 5

 0
 0
 1

 0
 0
 4

 0
 0
 2

 0
 0
 77

 0
 0
 1

 0
 0
 1

 0
 0
 3

 0
 0
 2

 0
 0
 1

 0
 0
 1

 3
 0
 1

 1

 2

 0
 0
 1

 6
 0
 5

 1

 1

 1

 3
 0
 1

 0
 0
 1

 0
 0
 1

 0
 0
 1

 0
 0
 1

 3
 301
 387

 2
 0
 60

 1

 0
 1

 0
 1

 0
 1

 0
 1

 0
 2

 0
 2

 0
 4

 0
 1

 0
 1

 0
 1

 0
 1

 0
 1

 0
 2

 0
 1

 0
 2

 0
 4

 0
 15

 0
 2

 0
 1

 0
 2

 0
 13

 0
 149

 0
 46

 0
 1

 0
 1

 0
 21

 0
 2

 0
 1

 0
 1

 0
 1

 0
 2

 0
 1

 0
 1

 0
 1

 0
 3

 0
 3

 0
 1

 0
 2

 0
 1

 0
 2

 0
 1

 0
 1

 0
 0
 1

 0
 0
 2

 0
 0
 1

 0
 0
 268

 0
 0
 47

 0
 0
 6

 0
 0
 1

 0
 0
 1

 2
 0
 5

 2

 0
 0
 1

 0
 0
 4

 0
 0
 1

 0
 0
 1

 0
 0
 1

 0
 0
 1

 183
 1
 8

 41

 1

 2

 1

 2

 1

 9

 9

 1

 2

 3

 5

 2

 1

 1

 1

 1

 1

 9

 8

 1

 1

 1

 22
 0
 2

 1

 18
 0
 1

 3

 0
 0
 1

 107
 1
 3

 1

 1

 1

 1

 1

 1

 1

 1

 1

 3

 1

 1

 1

 2

 3

 1

 1

 1

 2

 1

 17

 24

 1

 26

 1

 1

 1

 2

 3

 1

 1

 1

 1

 1

 0
 1

 0
 0
 2

 0
 0
 1

 1

 1

 1

 1

 0
 0
 1

 0
 0
 1

 0
 0
 2

 0
 0
 2

 3

 3

 1

 1

 1

 107
 0
 40

 1

 1

 106
 0
 40

 1

 1

 1

 1

 1

 1

 2

 1
 0
 1

 4

 4

 7
 0
 1

 7
 0
 2

 7

 16
 0
 2

 27

 2

 1

 2

 2
 0
 1

 4
 0
 2

 12

 1
 0
 1

 1

 0
 0
 1

 0
 0
 1

 0
 0
 18

 0
 0
 1

 0
 0
 1

 0
 0
 1

 0
 0
 3

 0
 0
 2

 0
 0
 1

 0
 0
 1

 49
 0
 8

 49
 0
 8

 1

 1

 4
 0
 1

 19
 0
 1

 13
 0
 3

 2
 0
 1

 1

 1

 2

 1

 1

 1

 1

 1

 0
 0
 1

 0
 0
 1

 51
 0
 62

 2
 0
 4

 2

 0
 0
 3

 0
 0
 1

 26
 0
 17

 1

 1

 1

 1

 1

 1

 2

 1

 2
 0
 3

 4

 6
 0
 13

 1

 2

 1

 1

 0
 0
 1

 23
 0
 6

 1

 13
 0
 1

 3
 0
 2

 6

 0
 0
 1

 0
 0
 2

 0
 0
 34

 0
 0
 1

 0
 0
 29

 0
 0
 3

 0
 0
 1

 0
 0
 1

 0
 0
 1

 415
 0
 116

 42
 0
 30

 1

 1

 30
 0
 13

 1

 4

 1

 1

 1

 1

 1

 0
 0
 2

 0
 0
 1

 0
 0
 1

 0
 0
 3

 0
 0
 1

 0
 0
 1

 0
 0
 6

 0
 0
 1

 0
 0
 1

 1
 0
 6

 1
 0
 2

 0
 0
 3

 0
 0
 1

 86
 0
 9

 1

 1

 1

 5

 50
 0
 4

 1

 3

 4

 17
 0
 5

 1

 1

 1

 73
 0
 17

 1

 1

 1

 1

 2
 0
 1

 1

 1

 1
 0
 1

 1

 16

 2
 0
 1

 1

 30

 1
 0
 2

 1

 1

 5

 1

 1
 0
 6

 1

 1

 1

 1

 0
 0
 2

 0
 0
 1

 0
 0
 3

 213
 0
 54

 1

 1

 1

 1

 1

 1

 1

 1
 0
 1

 1

 1

 1

 2
 0
 1

 3
 0
 2

 1

 1

 2
 0
 3

 2

 4
 0
 3

 2

 1

 1

 16
 0
 1

 1

 8
 0
 1

 1
 0
 1

 3

 5

 13
 0
 1

 7
 0
 1

 56
 0
 5

 12

 9
 0
 2

 10
 0
 1

 8
 0
 2

 1

 4

 1

 1

 3

 1

 4

 1
 0
 1

 1

 1
 0
 1

 3

 2

 1

 1

 1

 1

 1

 1

 2

 1

 1

 1

 0
 0
 1

 0
 0
 1

 0
 0
 1

 0
 0
 1

 0
 0
 1

 0
 0
 1

 0
 0
 1

 0
 0
 1

 0
 0
 1

 0
 0
 2

 0
 0
 3

 0
 0
 1

 0
 0
 3

 0
 0
 1

 0
 0
 1

 0
 0
 1

 0
 0
 1

 0
 0
 1

 0
 0
 1

 0
 0
 1

 0
 0
 1

 0
 0
 1

 1
 0
 26

 1
 0
 24

 1
 0
 13

 0
 0
 2

 0
 0
 1

 0
 0
 1

 0
 0
 3

 0
 0
 1

 0
 0
 2

 0
 0
 1

 0
 0
 1

 0
 0
 1

 0
 0
 1

 0
 0
 1

 4
 0
 7

 2
 0
 7

 2
 0
 7

 1

 1

 1

 1

 2
 62
 140

 1
 0
 22

 1

 0
 0
 1

 0
 0
 15

 0
 0
 6

 1
 55
 37

 1

 0
 1

 0
 6

 0
 8
 4

 0
 13
 1

 0
 10
 6

 0
 16
 4

 0
 1

 0
 0
 1

 0
 0
 1

 0
 0
 1

 0
 0
 2

 0
 0
 2

 0
 0
 11

 0
 0
 4

 0
 7
 56

 0
 4
 7

 0
 2

 0
 1
 1

 0
 0
 7

 0
 0
 1

 0
 0
 1

 0
 0
 1

 0
 0
 2

 0
 0
 1

 0
 0
 4

 0
 0
 3

 0
 0
 2

 0
 0
 1

 0
 0
 2

 0
 0
 6

 0
 0
 3

 0
 0
 3

 0
 0
 2

 0
 0
 1

 0
 0
 3

 0
 0
 1

 0
 0
 1

 0
 0
 1

 0
 0
 1

 0
 0
 1

 0
 0
 5

 0
 0
 1

 0
 0
 3

 0
 0
 1

 0
 0
 8

 0
 0
 5

 0
 0
 3

 0
 0
 2

 0
 0
 1

 0
 0
 1

 0
 0
 2

 0
 0
 2

 0
 0
 4

 0
 0
 4

 0
 0
 4

 0
 0
 2

 0
 0
 1

 0
 0
 1

 202
 0
 204

 1

 1

 29
 0
 64

 1

 1

 23
 0
 6

 1

 2

 1

 0
 0
 1

 0
 0
 1

 0
 0
 3

 0
 0
 2

 0
 0
 3

 0
 0
 5

 0
 0
 3

 0
 0
 40

 161
 0
 53

 1

 1

 1

 7

 1

 1

 2
 0
 33

 1

 1

 45
 0
 1

 36

 12

 12

 21

 12

 1

 1

 1

 1

 1

 1

 1

 0
 0
 1

 0
 0
 1

 0
 0
 4

 0
 0
 1

 0
 0
 1

 0
 0
 2

 0
 0
 3

 0
 0
 2

 0
 0
 1

 0
 0
 3

 11
 0
 14

 1

 1

 1

 1

 1

 2

 2

 1

 1

 0
 0
 1

 0
 0
 1

 0
 0
 4

 0
 0
 6

 0
 0
 1

 0
 0
 1

 0
 0
 3

 0
 0
 2

 0
 0
 1

 0
 0
 69

 0
 0
 4

 0
 0
 46

 0
 0
 18

 0
 0
 1

 0
 0
 1

 0
 0
 1

 15
 0
 3

 15
 0
 3

 1

 14
 0
 3

 1
 0
 1

 1
 0
 1

 1

 0
 0
 1

 5
 0
 97

 2
 0
 4

 2

 0
 0
 1

 0
 0
 3

 1
 0
 59

 1

 0
 0
 1

 0
 0
 1

 0
 0
 3

 0
 0
 1

 0
 0
 3

 0
 0
 10

 0
 0
 1

 0
 0
 2

 0
 0
 8

 0
 0
 2

 0
 0
 25

 0
 0
 1

 0
 0
 1

 2
 0
 34

 1

 1

 0
 0
 33

 0
 0
 1

 42
 0
 11

 23
 0
 5

 23
 0
 5

 19
 0
 6

 1

 1

 1

 6
 0
 4

 2

 8

 0
 0
 1

 0
 0
 1

 0
 0
 2

 0
 0
 2

 0
 0
 2

 0
 0
 5

 0
 0
 5

 0
 0
 5

 0
 0
 3

 0
 0
 3

 0
 0
 3

 0
 0
 2

 0
 0
 2

 0
 0
 2

 0
 0
 3

 0
 0
 1

 0
 0
 1

 0
 0
 2

 0
 0
 1

 0
 0
 1

 20
 99
 191
 65

 20
 99
 191
 65

 20
 97
 7
 62

 9

 4

 5
 0
 0
 19

 1

 1
 0
 0
 1

 0
 1

 0
 2

 0
 93

 0
 1

 0
 0
 1

 0
 0
 6
 17

 0
 0
 0
 1

 0
 0
 0
 1

 0
 0
 0
 1

 0
 0
 0
 1

 0
 0
 0
 1

 0
 0
 0
 13

 0
 0
 0
 1

 0
 0
 0
 3

 0
 0
 0
 1

 0
 0
 0
 1

 0
 0
 0
 1

 0
 2
 176
 3

 0
 2
 3

 0
 0
 173
 3

 0
 0
 8

 0
 0
 1

 0
 0
 6

 0
 0
 1

 5
 90
 47
 619

 5
 90
 47
 619

 1
 0
 0
 34

 1

 0
 0
 0
 1

 0
 0
 0
 4

 0
 0
 0
 8

 0
 0
 0
 17

 0
 0
 0
 3

 0
 0
 0
 1

 4
 14
 6
 19

 1

 2
 0
 1

 1

 0
 1

 0
 1

 0
 1

 0
 11
 2

 0
 0
 1
 4

 0
 0
 1

 0
 0
 1

 0
 0
 0
 13

 0
 0
 0
 1

 0
 0
 0
 1

 0
 33
 25

 0
 2

 0
 1

 0
 1

 0
 18
 3

 0
 7

 0
 2

 0
 2

 0
 0
 9

 0
 0
 3

 0
 0
 10

 0
 3
 13
 58

 0
 3

 0
 0
 1

 0
 0
 2

 0
 0
 2
 2

 0
 0
 1

 0
 0
 2

 0
 0
 3

 0
 0
 1

 0
 0
 1

 0
 0
 0
 8

 0
 0
 0
 1

 0
 0
 0
 1

 0
 0
 0
 6

 0
 0
 0
 1

 0
 0
 0
 1

 0
 0
 0
 15

 0
 0
 0
 1

 0
 0
 0
 1

 0
 0
 0
 1

 0
 0
 0
 4

 0
 0
 0
 8

 0
 0
 0
 4

 0
 0
 0
 2

 0
 0
 0
 1

 0
 0
 0
 1

 0
 15
 1

 0
 15
 1

 0
 25
 0
 251

 0
 24

 0
 1

 0
 0
 0
 2

 0
 0
 0
 248

 0
 0
 0
 1

 0
 0
 2
 54

 0
 0
 2
 54

 0
 0
 0
 12

 0
 0
 0
 12

 0
 0
 0
 18

 0
 0
 0
 18

 0
 0
 0
 5

 0
 0
 0
 5

 0
 0
 0
 168

 0
 0
 0
 151

 0
 0
 0
 17

 86
 0
 52

 86
 0
 52

 86
 0
 52

 1

 3

 1

 1

 1

 1

 1

 1

 2

 1

 1

 1

 1

 1

 1

 1

 1

 1

 2
 0
 4

 1

 3
 0
 1

 1

 2

 1

 1

 1

 1

 3
 0
 1

 2

 9
 0
 1

 2

 1

 1

 2

 1

 1

 2

 1
 0
 6

 1

 1

 1
 0
 2

 8
 0
 1

 1

 1

 3
 0
 1

 1

 1

 1

 1

 1

 1

 1

 1
 0
 1

 1

 1

 1

 0
 0
 1

 0
 0
 1

 0
 0
 1

 0
 0
 1

 0
 0
 1

 0
 0
 1

 0
 0
 1

 0
 0
 1

 0
 0
 1

 0
 0
 1

 0
 0
 1

 0
 0
 1

 0
 0
 2

 0
 0
 2

 0
 0
 1

 0
 0
 1

 0
 0
 2

 0
 0
 1

 0
 0
 1

 0
 0
 3

 0
 0
 1

 0
 0
 1

 0
 0
 1

 0
 0
 1

 0
 0
 1

 0
 0
 2

 0
 0
 1

 0
 0
 1

 11
 0
 3

 11
 0
 3

 11
 0
 3

 1

 9

 1

 0
 0
 1

 0
 0
 1

 0
 0
 1

 194
 0
 53

 72
 0
 13

 72
 0
 13

 1

 1

 1

 1

 1

 1

 1

 1

 3

 1

 1

 1

 1

 8

 1

 4

 17
 0
 3

 6
 0
 1

 1

 1

 1

 2

 5

 2
 0
 2

 1

 1

 1

 3

 1

 1

 1

 0
 0
 1

 0
 0
 2

 0
 0
 1

 0
 0
 1

 0
 0
 1

 0
 0
 1

 6
 0
 13

 6
 0
 13

 2
 0
 12

 1

 2

 1

 0
 0
 1

 65
 0
 8

 65
 0
 8

 1

 1

 1

 2

 1

 1

 1

 1

 1

 3

 2

 1

 9
 0
 1

 26
 0
 1

 1

 7

 2

 2

 1

 1

 0
 0
 5

 0
 0
 1

 51
 0
 19

 51
 0
 19

 1

 1

 1

 1

 1

 3

 1

 1

 3

 2

 1
 0
 2

 4
 0
 2

 1

 2

 2
 0
 2

 1

 1

 1

 3
 0
 3

 1

 2

 1

 1

 1
 0
 1

 6

 6

 1

 1
 0
 2

 0
 0
 1

 0
 0
 1

 0
 0
 1

 0
 0
 1

 0
 0
 2

 0
 0
 1

 80
 0
 20
 11

 80
 0
 20
 11

 80
 0
 20
 11

 80
 0
 20
 11

 1

 2

 1

 1

 1

 1

 1

 2

 1

 1

 1
 0
 1

 1

 3

 1

 1

 1

 2

 20
 0
 1

 22
 0
 1

 2

 2

 1

 4
 0
 1

 1

 1

 1

 1
 0
 1

 1

 1

 1

 0
 0
 1

 0
 0
 5

 0
 0
 1

 0
 0
 1

 0
 0
 1

 0
 0
 1

 0
 0
 1

 0
 0
 1

 0
 0
 1

 0
 0
 1

 0
 0
 1

 0
 0
 0
 11

 1

 1

 1

 1

 1

 123
 0
 9

 15
 0
 1

 15
 0
 1

 15
 0
 1

 15
 0
 1

 1

 2

 1

 1

 1

 4

 1

 1

 1

 1

 1

 0
 0
 1

 16
 0
 1

 16
 0
 1

 16
 0
 1

 16
 0
 1

 1

 1

 1

 1

 4

 4

 1

 1

 1

 1

 0
 0
 1

 78
 0
 6

 78
 0
 6

 78
 0
 6

 78
 0
 6

 1

 1

 1

 1

 1

 1

 1

 2

 1

 2

 1

 1

 1

 1

 1

 1

 4

 3

 1

 2

 3

 1

 14

 2

 1

 1

 1

 1

 1

 1

 1

 1

 1

 1

 1

 1

 1

 1

 1

 1

 3

 1

 1

 1

 1

 2

 1

 1

 1

 1

 1

 0
 0
 1

 0
 0
 1

 0
 0
 2

 0
 0
 1

 0
 0
 1

 12
 0
 1

 12
 0
 1

 12
 0
 1

 12
 0
 1

 1

 1

 1

 3

 1

 3

 1

 1

 0
 0
 1

 2

 2

 2

 2

 1

 1

 2885
 148
 611
 490

 2885
 148
 611
 490

 2885
 148
 611
 490

 2885
 148
 611
 490

 2885
 148
 611
 490

 1

 1

 1

 1

 1

 1

 1

 1

 1

 1

 1

 1

 1

 1

 1

 1

 1

 1

 1

 1

 1

 1

 1

 1

 1

 1

 1

 1

 1

 1

 1

 1

 1

 2

 1

 1

 1

 1

 1

 1

 1

 1

 1

 1

 1

 1

 1

 1

 1

 1

 1

 1

 1

 1

 1

 1

 1

 1

 2

 1

 1

 1

 1

 1

 1

 1

 1

 1

 2

 1

 1

 1

 1

 1

 1

 1

 1

 1

 1

 1

 1

 1

 2

 1

 1

 1

 1

 1

 1

 1

 1

 1

 1

 1

 1

 1

 1

 1

 1

 1

 1

 1

 1

 1

 1

 1

 1

 1

 1

 1

 1

 1

 1

 1

 2

 1

 1

 1

 1

 1

 1

 1

 1

 1

 1

 1

 1

 1

 1

 1

 1

 1

 1

 1

 1

 1

 1

 1

 1

 2

 1

 2

 1

 1

 1

 1

 1

 1

 1

 1

 1

 1

 2

 1

 1

 1

 1

 1

 1

 1

 1

 2

 1

 1

 1

 1

 1

 1

 1

 1

 1

 1

 1

 2

 1

 1

 1

 1

 1
 0
 1

 1

 1

 1

 1

 1

 1

 1

 1

 1

 1

 1

 1

 1

 1

 1

 2

 1

 1

 1

 1

 1

 1

 1

 1

 1

 4

 1

 1

 1

 1

 2

 9

 1

 2

 1

 2

 1

 1

 1

 1

 1

 1

 1

 1

 1

 1

 1

 1

 1

 2

 1

 1

 1

 1

 1

 1

 3

 1

 1

 1

 3

 1

 1

 1

 1

 1

 2

 1

 1

 1

 4

 1

 1

 1

 1

 1

 1

 1

 1

 1

 1

 1

 1

 1

 1

 2

 2

 3

 1

 5

 2

 1

 1

 1

 2

 5

 1

 1

 4

 1

 1
 0
 1

 2

 1

 3

 3

 1

 1

 1

 1

 2

 2

 273

 1

 3

 1

 1

 3

 2

 1

 7

 1

 1

 1

 2

 1

 1

 3

 3

 1

 1

 1

 1

 1

 1

 1

 1

 1

 1

 1
 0
 2

 1

 1

 1

 1

 1

 1

 1

 1

 1

 2

 1

 1

 1

 1

 1

 1

 1

 2

 1

 1

 1

 1

 2

 4

 1

 1

 1

 1

 1

 1

 1

 1

 1

 2

 1
 0
 1

 1

 1

 2

 1

 1

 1

 1

 1

 1

 1

 1

 1

 1

 1

 1

 1

 1

 1

 3

 1

 1

 1

 1

 2

 1

 1

 1

 2

 1

 1

 1

 1

 5

 2

 1

 1

 1

 1

 2

 1

 1

 1

 1

 1

 1

 2

 1

 1
 0
 0
 34

 1

 1

 1

 1

 1

 1

 1

 1

 1

 3

 1

 1

 1

 1

 1

 1

 1

 1

 1

 1

 1

 1

 1

 1

 6

 1

 5

 3

 1

 1

 1

 1

 3

 1

 3

 1

 1

 1

 1

 2

 2

 1

 1

 1

 3

 2

 2

 1

 6

 1

 1

 1

 1

 1
 0
 2

 2

 1

 1

 1

 2

 3

 1

 2

 4
 0
 1

 1

 1

 1

 6

 1

 4

 1

 4

 1

 5

 4

 2

 1

 3

 2
 0
 2

 1

 1

 1

 2

 2

 1

 1

 1

 1

 6

 5

 2
 0
 1

 1

 1

 2

 1

 3

 1

 3
 0
 3

 1

 1

 1

 2

 1

 2

 1

 2

 1

 1

 1

 1

 1

 1

 1

 1

 1

 1

 1

 1

 1

 2
 0
 1

 3
 0
 3

 1

 1

 2

 2

 1
 0
 1

 2

 5

 1

 1

 1

 1

 1

 1

 1

 1

 1

 1

 1

 1

 1

 1

 1

 1

 1

 1

 1

 2

 1

 1

 1

 1

 1

 2

 1

 2

 1

 1

 2

 1

 2

 1

 1

 1

 1

 1

 4

 2

 1

 1

 3

 1

 8

 1

 2

 1

 3

 2

 1
 0
 1

 1

 1

 6

 2

 1

 3

 1

 7
 0
 1

 42
 0
 1

 18

 1

 10

 11
 0
 1

 2

 1

 3

 1

 1

 3

 1

 1

 3

 1

 2
 0
 1

 1

 22
 0
 1

 4

 1

 3

 69

 2

 1

 1

 16

 1

 3

 1

 1

 2

 1

 1

 1

 7

 1

 2

 9

 7
 0
 1

 1

 2

 5

 1

 5

 1

 1

 3
 0
 1

 12

 1

 1

 16

 2

 1

 1

 1

 1

 2

 2

 4

 1

 1

 1

 1

 16

 3

 3

 5

 5

 4
 0
 2

 17

 6

 12

 1

 3
 0
 2

 3

 24

 4

 1

 1

 5

 7

 4

 3

 1

 4

 1

 3

 2

 3

 15

 10

 3

 44

 2

 2

 3

 2

 1

 1

 43
 0
 2

 2

 2

 8

 1

 2

 1

 13

 5

 9

 10
 0
 2

 28
 0
 2

 1

 3

 22

 1

 9

 15

 3

 1

 2

 1

 62
 0
 59

 1

 12

 1

 1

 6
 0
 3

 5

 3

 1

 1

 1

 2

 4

 1

 13

 1

 1

 1

 1

 1

 5

 1

 3
 0
 1

 5

 2

 2

 2

 3

 2

 2

 1

 1

 1

 1

 4

 6

 2

 1

 1

 4

 1

 10

 1

 4

 2

 1

 1

 1

 1

 2

 3

 1

 3
 0
 1

 1

 2

 2

 2

 1

 3

 2

 9
 0
 32

 1

 5

 7

 2

 2

 1

 2

 1

 1

 6

 1
 0
 1

 1
 0
 1

 4

 1

 6

 5

 6

 1

 1

 2

 4

 2

 4

 1
 0
 2

 1

 4

 1

 4

 1
 0
 1

 3

 8

 4

 1

 1

 2

 4

 1

 1

 2

 8

 9

 3

 1

 1

 3

 1

 7

 2
 0
 1

 1

 2

 1

 1

 1

 1

 1

 1

 1

 2

 1

 1

 2

 1

 1

 1

 1

 1

 1

 1

 1

 1

 1

 2

 1

 2

 2

 1

 1

 1

 1

 2

 4

 1

 1

 1
 0
 1

 1

 1

 2

 2

 2

 8

 1

 1

 2

 1

 3

 1

 3

 1

 1

 1

 1

 1

 3

 1

 1

 1

 6

 1

 2

 1

 1

 1

 1

 1

 2

 1

 1

 1

 1

 1

 1

 1

 1

 1

 1

 1

 1

 1

 1

 2

 2

 1

 1

 6

 1

 1

 2

 1

 1

 1

 1

 1

 1

 1

 1

 1

 1

 1

 1

 1

 2

 3

 1

 1

 6

 3

 1

 2

 1

 1

 1

 1

 3

 1

 1

 3

 4

 27

 1

 1

 2
 0
 1

 2

 4

 2

 3

 1

 1

 1

 5

 1

 2

 1

 6

 1

 1

 2
 0
 2

 1

 1

 2

 2

 2

 1

 1

 1

 4

 1

 1

 1

 1

 1

 1

 1

 1

 1
 0
 1

 1

 1

 1
 0
 2

 3

 5

 1

 1

 1

 4

 1

 3

 1

 1

 2

 1

 1

 1

 1

 1

 1

 1

 2

 2

 37

 1

 1

 1

 2

 1

 1

 1

 2

 1

 2

 1

 1

 1

 1

 2

 1

 2

 1

 1

 1

 1

 1

 1

 1

 1

 1

 1

 1

 1

 2

 1

 1

 1

 1

 1

 1

 1

 1

 1

 1

 1

 1

 1

 1

 1

 1

 1

 1

 1

 1

 1

 1

 1

 1

 1

 1

 1

 1

 1

 1

 1

 1

 2

 1

 1

 1

 1

 1

 1

 1

 1

 1

 1

 1

 1

 1

 1

 1

 1

 1

 1

 1

 1

 1

 1

 1

 1

 2

 3

 1

 1

 1

 1

 1

 1

 1

 1

 1

 1

 1

 1

 2

 1

 1

 1

 1

 1

 1

 1

 1

 1

 1

 1

 1

 1

 1

 4

 1

 1

 1

 1

 1

 1
 0
 1

 1

 2

 1

 2

 1

 1

 4

 1

 1

 1

 1

 1

 1

 1

 1

 1

 1

 1

 1

 1

 1

 1

 1

 1

 1

 1

 2

 2

 1

 1

 1

 1

 1

 1

 1

 2

 1

 1

 1

 1

 1

 1

 1

 1

 1

 2

 1

 1

 1

 1

 1

 3

 1

 1

 1

 1

 1

 1

 1

 2

 1

 1

 5

 1

 1

 1

 1

 1

 1

 1

 1

 1

 1

 1

 2

 1

 1

 5

 1

 1

 1

 1

 1

 1

 1

 1

 1

 1

 1
 0
 1

 1

 1

 1

 1

 3

 1

 1

 1

 1

 1

 1

 1

 1

 1

 1

 2

 1

 1

 1

 1

 1

 2

 1

 1

 1

 1

 1

 1

 1

 1

 1

 1

 1

 1

 1

 1

 1

 1

 1

 1

 1

 1

 1

 1

 1

 1

 1

 1

 1

 1

 2

 1

 1

 1

 1

 1

 1

 2

 1

 1

 1

 1

 1

 1

 1

 1

 1

 1

 1

 1

 1

 1

 1

 1

 1

 1

 1

 1

 1

 1

 1

 1

 1

 1

 1

 1

 1

 1

 1

 1

 1

 1

 1

 1

 1

 1

 1

 1

 1

 1

 1

 1

 1

 1

 1

 1

 1

 1

 1

 1

 1

 1

 1

 1

 1

 1

 1

 1

 1

 1

 1

 1

 1

 0
 1

 0
 8

 0
 2

 0
 1

 0
 5
 1

 0
 3

 0
 3

 0
 1

 0
 1

 0
 5

 0
 11
 1

 0
 1

 0
 2
 2

 0
 10
 4

 0
 10

 0
 8
 1

 0
 47

 0
 7

 0
 4

 0
 6

 0
 2

 0
 3
 2

 0
 2

 0
 1

 0
 1
 1

 0
 3

 0
 0
 1

 0
 0
 1

 0
 0
 1

 0
 0
 1

 0
 0
 1

 0
 0
 1

 0
 0
 1

 0
 0
 1

 0
 0
 1

 0
 0
 1

 0
 0
 1

 0
 0
 1

 0
 0
 1

 0
 0
 1

 0
 0
 1

 0
 0
 1

 0
 0
 1

 0
 0
 1

 0
 0
 1

 0
 0
 1

 0
 0
 1

 0
 0
 1

 0
 0
 1

 0
 0
 1

 0
 0
 1

 0
 0
 1

 0
 0
 1

 0
 0
 1

 0
 0
 1

 0
 0
 1

 0
 0
 1

 0
 0
 1

 0
 0
 2

 0
 0
 1

 0
 0
 1

 0
 0
 1

 0
 0
 5

 0
 0
 3

 0
 0
 1

 0
 0
 1

 0
 0
 1

 0
 0
 1

 0
 0
 1

 0
 0
 1

 0
 0
 1

 0
 0
 1

 0
 0
 3

 0
 0
 1

 0
 0
 1

 0
 0
 1

 0
 0
 1

 0
 0
 1

 0
 0
 1

 0
 0
 1

 0
 0
 1

 0
 0
 2

 0
 0
 1

 0
 0
 5

 0
 0
 1

 0
 0
 1

 0
 0
 1

 0
 0
 1

 0
 0
 1

 0
 0
 1

 0
 0
 2

 0
 0
 1

 0
 0
 1

 0
 0
 1

 0
 0
 1

 0
 0
 1

 0
 0
 2

 0
 0
 2

 0
 0
 1

 0
 0
 1

 0
 0
 7

 0
 0
 1

 0
 0
 1

 0
 0
 1

 0
 0
 2

 0
 0
 1

 0
 0
 3

 0
 0
 1

 0
 0
 1

 0
 0
 3

 0
 0
 1

 0
 0
 1

 0
 0
 1

 0
 0
 3
 1

 0
 0
 1

 0
 0
 1

 0
 0
 1

 0
 0
 1

 0
 0
 2

 0
 0
 1

 0
 0
 1

 0
 0
 3

 0
 0
 1

 0
 0
 1

 0
 0
 1

 0
 0
 1

 0
 0
 1

 0
 0
 1

 0
 0
 1

 0
 0
 1

 0
 0
 1

 0
 0
 1

 0
 0
 3

 0
 0
 1

 0
 0
 4

 0
 0
 1

 0
 0
 1

 0
 0
 6

 0
 0
 1

 0
 0
 10

 0
 0
 1

 0
 0
 1

 0
 0
 1

 0
 0
 1

 0
 0
 5

 0
 0
 4

 0
 0
 1

 0
 0
 1

 0
 0
 3

 0
 0
 65

 0
 0
 5

 0
 0
 9

 0
 0
 1

 0
 0
 3

 0
 0
 1

 0
 0
 1

 0
 0
 2

 0
 0
 2

 0
 0
 5

 0
 0
 1

 0
 0
 2

 0
 0
 2

 0
 0
 1

 0
 0
 1

 0
 0
 1

 0
 0
 1

 0
 0
 1

 0
 0
 1

 0
 0
 2

 0
 0
 2

 0
 0
 1

 0
 0
 1

 0
 0
 6

 0
 0
 1

 0
 0
 6

 0
 0
 1

 0
 0
 3

 0
 0
 1

 0
 0
 1

 0
 0
 1

 0
 0
 1

 0
 0
 1

 0
 0
 1

 0
 0
 1

 0
 0
 1

 0
 0
 2

 0
 0
 1

 0
 0
 6

 0
 0
 1

 0
 0
 1

 0
 0
 1

 0
 0
 1

 0
 0
 1

 0
 0
 1

 0
 0
 2

 0
 0
 1

 0
 0
 1

 0
 0
 1

 0
 0
 1

 0
 0
 1

 0
 0
 1

 0
 0
 1

 0
 0
 1

 0
 0
 1

 0
 0
 2

 0
 0
 1

 0
 0
 1

 0
 0
 1

 0
 0
 1

 0
 0
 1

 0
 0
 1

 0
 0
 1

 0
 0
 1
 6

 0
 0
 1

 0
 0
 1

 0
 0
 1

 0
 0
 1

 0
 0
 1

 0
 0
 1

 0
 0
 1

 0
 0
 1

 0
 0
 1

 0
 0
 2

 0
 0
 1

 0
 0
 1

 0
 0
 3

 0
 0
 1

 0
 0
 1

 0
 0
 1

 0
 0
 1

 0
 0
 1

 0
 0
 1

 0
 0
 1

 0
 0
 1

 0
 0
 1

 0
 0
 1

 0
 0
 2

 0
 0
 1

 0
 0
 1

 0
 0
 2

 0
 0
 1

 0
 0
 1

 0
 0
 1

 0
 0
 1

 0
 0
 1

 0
 0
 2

 0
 0
 2

 0
 0
 1

 0
 0
 1

 0
 0
 1

 0
 0
 1

 0
 0
 1

 0
 0
 1

 0
 0
 1

 0
 0
 1

 0
 0
 2

 0
 0
 1

 0
 0
 2

 0
 0
 1

 0
 0
 1

 0
 0
 1

 0
 0
 1

 0
 0
 1

 0
 0
 1

 0
 0
 1

 0
 0
 3

 0
 0
 1

 0
 0
 1

 0
 0
 1

 0
 0
 1

 0
 0
 2

 0
 0
 1

 0
 0
 1

 0
 0
 1

 0
 0
 1

 0
 0
 1

 0
 0
 1

 0
 0
 1

 0
 0
 2

 0
 0
 1

 0
 0
 1

 0
 0
 2

 0
 0
 2

 0
 0
 1

 0
 0
 1

 0
 0
 1

 0
 0
 1

 0
 0
 1

 0
 0
 2

 0
 0
 1

 0
 0
 1

 0
 0
 1

 0
 0
 0
 5

 0
 0
 0
 1

 0
 0
 0
 1

 0
 0
 0
 1

 0
 0
 0
 7

 0
 0
 0
 9

 0
 0
 0
 1

 0
 0
 0
 1

 0
 0
 0
 1

 0
 0
 0
 2

 0
 0
 0
 21

 0
 0
 0
 1

 0
 0
 0
 1

 0
 0
 0
 1

 0
 0
 0
 3

 0
 0
 0
 5

 0
 0
 0
 1

 0
 0
 0
 1

 0
 0
 0
 1

 0
 0
 0
 1

 0
 0
 0
 1

 0
 0
 0
 1

 0
 0
 0
 1

 0
 0
 0
 1

 0
 0
 0
 5

 0
 0
 0
 1

 0
 0
 0
 1

 0
 0
 0
 5

 0
 0
 0
 8

 0
 0
 0
 1

 0
 0
 0
 5

 0
 0
 0
 5

 0
 0
 0
 1

 0
 0
 0
 22

 0
 0
 0
 6

 0
 0
 0
 29

 0
 0
 0
 1

 0
 0
 0
 20

 0
 0
 0
 3

 0
 0
 0
 121

 0
 0
 0
 13

 0
 0
 0
 3

 0
 0
 0
 18

 0
 0
 0
 10

 0
 0
 0
 1

 0
 0
 0
 1

 0
 0
 0
 7

 0
 0
 0
 1

 0
 0
 0
 6

 0
 0
 0
 1

 0
 0
 0
 1

 0
 0
 0
 7

 0
 0
 0
 3

 0
 0
 0
 6

 0
 0
 0
 1

 0
 0
 0
 1

 0
 0
 0
 1

 0
 0
 0
 1

 0
 0
 0
 1

 0
 0
 0
 4

 0
 0
 0
 5

 0
 0
 0
 1

 0
 0
 0
 3

 0
 0
 0
 4

 0
 0
 0
 2

 0
 0
 0
 1

 0
 0
 0
 1

 0
 0
 0
 9

 0
 0
 0
 6

 0
 0
 0
 3

 0
 0
 0
 2

 0
 0
 0
 4

 0
 0
 0
 14

 0
 0
 0
 1

 0
 0
 0
 3

 4455
 4607
 1119
 7884

 372
 104
 69
 556

 372
 104
 69
 556

 372
 104
 69
 556

 372
 104
 69
 556

 1

 1

 1

 1

 1

 1

 1

 2

 1

 1

 3

 1

 1

 1

 2

 1

 1

 1

 1

 1

 1

 1

 1

 1

 1

 5
 0
 1

 2

 1

 1
 0
 1

 1

 1

 1

 5

 2

 1

 1

 2

 1

 1

 1

 3

 2

 1

 1
 0
 1

 1

 1

 1

 1

 1

 1

 2

 1

 2

 1

 1

 3
 0
 3

 1

 1

 2

 1

 3

 1

 1

 2

 1
 0
 1

 1

 1

 7

 2

 4

 1

 3

 1

 1

 2

 1

 12

 1

 1

 4
 0
 1

 1

 1

 1

 1

 1

 2

 2

 2

 2

 4

 1
 0
 1

 1

 2

 3

 1

 8

 1

 5

 1

 6

 1

 13

 1

 1

 1

 1
 0
 1

 2

 1

 1

 11

 1

 4

 1
 0
 1

 5

 3

 2
 0
 1

 5

 4

 5
 0
 1

 3
 0
 1

 1

 1

 5

 1

 2

 4

 1
 0
 1

 1

 1

 2

 7

 1

 1

 1

 1

 5

 1

 1

 1

 2

 1

 2

 1

 6

 1

 3

 2

 3

 1

 5

 1

 1

 1

 5

 1

 2

 1

 1

 1

 4

 2

 1

 1

 1

 2

 1

 1

 1

 1

 1

 1

 1

 1

 2

 1

 1
 0
 1

 1

 1

 1

 1
 0
 1

 1

 1

 1

 1

 1

 1

 1

 1

 1

 1

 1

 1

 1

 1

 1

 1

 1

 1

 1

 1

 1

 0
 1

 0
 48

 0
 40
 4

 0
 8

 0
 3

 0
 2

 0
 2

 0
 0
 1

 0
 0
 1

 0
 0
 1

 0
 0
 1

 0
 0
 1

 0
 0
 1

 0
 0
 1

 0
 0
 3

 0
 0
 3

 0
 0
 20

 0
 0
 1

 0
 0
 1

 0
 0
 1

 0
 0
 1

 0
 0
 1

 0
 0
 1

 0
 0
 1

 0
 0
 1

 0
 0
 1

 0
 0
 1

 0
 0
 1

 0
 0
 1

 0
 0
 1

 0
 0
 2

 0
 0
 0
 2

 0
 0
 0
 4

 0
 0
 0
 1

 0
 0
 0
 1

 0
 0
 0
 13

 0
 0
 0
 1

 0
 0
 0
 58

 0
 0
 0
 11

 0
 0
 0
 137

 0
 0
 0
 77

 0
 0
 0
 12

 0
 0
 0
 197

 0
 0
 0
 2

 0
 0
 0
 2

 0
 0
 0
 38

 399
 2960
 498
 7328

 399
 2960
 498
 7328

 101
 0
 11
 526

 101
 0
 11
 525

 1

 3

 1

 2
 0
 1

 3

 6
 0
 0
 4

 4

 1

 3

 3

 2

 5

 1

 1

 5

 3
 0
 1

 3

 1

 8

 1

 5

 1

 11
 0
 3

 13

 2

 3
 0
 1

 2

 3

 1

 3

 0
 0
 1
 1

 0
 0
 2

 0
 0
 2
 118

 0
 0
 0
 1

 0
 0
 0
 2

 0
 0
 0
 1

 0
 0
 0
 1

 0
 0
 0
 2

 0
 0
 0
 1

 0
 0
 0
 1

 0
 0
 0
 1

 0
 0
 0
 7

 0
 0
 0
 1

 0
 0
 0
 1

 0
 0
 0
 3

 0
 0
 0
 2

 0
 0
 0
 1

 0
 0
 0
 1

 0
 0
 0
 2

 0
 0
 0
 2

 0
 0
 0
 1

 0
 0
 0
 1

 0
 0
 0
 1

 0
 0
 0
 1

 0
 0
 0
 110

 0
 0
 0
 1

 0
 0
 0
 1

 0
 0
 0
 1

 0
 0
 0
 9

 0
 0
 0
 4

 0
 0
 0
 1

 0
 0
 0
 15

 0
 0
 0
 3

 0
 0
 0
 11

 0
 0
 0
 1

 0
 0
 0
 1

 0
 0
 0
 1

 0
 0
 0
 2

 0
 0
 0
 1

 0
 0
 0
 4

 0
 0
 0
 5

 0
 0
 0
 25

 0
 0
 0
 50

 0
 0
 0
 1

 0
 0
 0
 2

 0
 0
 0
 1

 0
 0
 0
 78

 0
 0
 0
 2

 0
 0
 0
 2

 0
 0
 0
 1

 0
 0
 0
 2

 0
 0
 0
 5

 0
 0
 0
 1

 0
 0
 0
 3

 0
 0
 0
 1

 0
 0
 0
 1

 0
 0
 0
 1

 0
 0
 0
 1

 0
 0
 0
 1

 0
 0
 0
 2

 0
 0
 0
 1

 0
 0
 0
 1

 0
 0
 0
 3

 0
 0
 0
 1

 0
 0
 0
 1

 0
 0
 0
 1

 0
 0
 0
 1

 0
 0
 0
 4

 0
 0
 0
 3

 0
 0
 0
 1

 0
 0
 0
 1

 0
 0
 0
 1

 143
 20
 18
 903

 143
 20
 18
 903

 1

 1

 1

 1

 2

 1

 2

 1

 2

 1

 1

 1

 1

 1

 1

 1

 1

 1

 1

 5

 1

 2

 1

 1

 1

 6

 11

 5

 1

 4

 3
 0
 2

 6

 1

 1

 33
 0
 5

 1
 0
 1

 2

 18

 1

 3

 1

 1

 1

 6

 1

 1
 0
 0
 2

 1
 0
 1

 1

 1

 0
 2
 1

 0
 4

 0
 9

 0
 1

 0
 2

 0
 2

 0
 0
 1

 0
 0
 1
 246

 0
 0
 1

 0
 0
 2

 0
 0
 1
 121

 0
 0
 1
 113

 0
 0
 1

 0
 0
 0
 1

 0
 0
 0
 1

 0
 0
 0
 1

 0
 0
 0
 2

 0
 0
 0
 86

 0
 0
 0
 54

 0
 0
 0
 79

 0
 0
 0
 191

 0
 0
 0
 1

 0
 0
 0
 1

 0
 0
 0
 3

 0
 0
 0
 1

 1

 1

 1

 64
 416
 131
 1640

 1
 0
 1
 693

 1

 0
 0
 1
 278

 0
 0
 0
 6

 0
 0
 0
 26

 0
 0
 0
 1

 0
 0
 0
 1

 0
 0
 0
 204

 0
 0
 0
 4

 0
 0
 0
 73

 0
 0
 0
 72

 0
 0
 0
 27

 0
 0
 0
 1

 10
 0
 1

 1

 7
 0
 1

 1

 1

 1

 1

 6
 0
 2

 1
 0
 1

 2

 1

 1

 1

 0
 0
 1

 7
 0
 1
 57

 3
 0
 1
 6

 4

 0
 0
 0
 6

 0
 0
 0
 16

 0
 0
 0
 29

 6

 3

 2

 1

 30
 26
 9
 667

 1

 1

 2

 2

 1

 1

 1

 1

 1

 2

 1

 1

 1

 1

 2

 3

 1

 1
 0
 1

 1

 1

 1

 1

 1

 1

 0
 1

 0
 1

 0
 2

 0
 1

 0
 10

 0
 1

 0
 2

 0
 1

 0
 2

 0
 1

 0
 1

 0
 1

 0
 1

 0
 1

 0
 0
 2

 0
 0
 1

 0
 0
 3

 0
 0
 1

 0
 0
 1

 0
 0
 0
 1

 0
 0
 0
 2

 0
 0
 0
 1

 0
 0
 0
 1

 0
 0
 0
 1

 0
 0
 0
 1

 0
 0
 0
 12

 0
 0
 0
 1

 0
 0
 0
 3

 0
 0
 0
 2

 0
 0
 0
 6

 0
 0
 0
 1

 0
 0
 0
 8

 0
 0
 0
 15

 0
 0
 0
 8

 0
 0
 0
 28

 0
 0
 0
 49

 0
 0
 0
 379

 0
 0
 0
 20

 0
 0
 0
 51

 0
 0
 0
 7

 0
 0
 0
 18

 0
 0
 0
 10

 0
 0
 0
 41

 0
 0
 0
 1

 3
 221
 87
 6

 1
 0
 16

 1
 110
 20

 1

 0
 1

 0
 1

 0
 1

 0
 1

 0
 8
 1

 0
 9
 2

 0
 4

 0
 2

 0
 59

 0
 2

 0
 4

 0
 11

 0
 4

 0
 2

 0
 2

 0
 0
 1

 0
 0
 1
 6

 0
 0
 6

 0
 0
 5

 0
 0
 27

 0
 0
 5

 0
 0
 1

 0
 0
 2

 0
 5
 0
 3

 0
 5

 0
 0
 0
 1

 0
 0
 0
 2

 0
 164
 30

 0
 1

 0
 16

 0
 17

 0
 127
 30

 0
 2

 0
 1

 0
 0
 0
 192

 0
 0
 0
 1

 0
 0
 0
 3

 0
 0
 0
 5

 0
 0
 0
 32

 0
 0
 0
 20

 0
 0
 0
 38

 0
 0
 0
 42

 0
 0
 0
 1

 0
 0
 0
 26

 0
 0
 0
 16

 0
 0
 0
 8

 0
 0
 0
 22

 0
 0
 0
 22

 57
 2520
 332
 4058

 11
 116
 15
 1319

 1

 10
 0
 0
 1

 0
 7
 2

 0
 2

 0
 4
 2

 0
 6

 0
 42
 7

 0
 38

 0
 2

 0
 9

 0
 5

 0
 1

 0
 0
 4
 551

 0
 0
 0
 4

 0
 0
 0
 36

 0
 0
 0
 2

 0
 0
 0
 282

 0
 0
 0
 442

 0
 0
 0
 1

 6
 0
 3
 373

 1

 1

 2

 2

 0
 0
 2

 0
 0
 1

 0
 0
 0
 45

 0
 0
 0
 1

 0
 0
 0
 1

 0
 0
 0
 1

 0
 0
 0
 1

 0
 0
 0
 2

 0
 0
 0
 1

 0
 0
 0
 7

 0
 0
 0
 2

 0
 0
 0
 244

 0
 0
 0
 2

 0
 0
 0
 46

 0
 0
 0
 13

 0
 0
 0
 1

 0
 0
 0
 1

 0
 0
 0
 1

 0
 0
 0
 1

 0
 0
 0
 1

 0
 0
 0
 2

 28
 2366
 296
 1691

 1

 1

 1

 1
 61
 16

 4
 0
 1
 254

 1
 0
 21
 10

 10

 1
 0
 35

 4

 1

 1

 1

 1

 0
 1

 0
 1

 0
 1

 0
 1

 0
 3

 0
 2

 0
 3

 0
 2

 0
 1

 0
 1

 0
 1

 0
 2

 0
 1

 0
 1

 0
 1

 0
 1

 0
 1

 0
 6

 0
 2

 0
 1

 0
 1

 0
 4

 0
 3

 0
 7

 0
 1

 0
 2

 0
 1

 0
 2

 0
 1

 0
 4

 0
 2

 0
 1

 0
 1

 0
 1

 0
 1

 0
 1

 0
 1

 0
 1

 0
 1

 0
 1

 0
 1

 0
 1

 0
 2

 0
 9

 0
 1
 3

 0
 6

 0
 1

 0
 2

 0
 10

 0
 2

 0
 1

 0
 4

 0
 3

 0
 3

 0
 1

 0
 1

 0
 2

 0
 2

 0
 3

 0
 11

 0
 9

 0
 1

 0
 14
 1

 0
 2

 0
 1

 0
 3

 0
 1

 0
 10

 0
 1

 0
 2

 0
 9

 0
 5

 0
 4

 0
 72
 3

 0
 4

 0
 2

 0
 41
 1

 0
 17

 0
 39

 0
 77
 1

 0
 12

 0
 12

 0
 11

 0
 17

 0
 10

 0
 18

 0
 38

 0
 1

 0
 6

 0
 209
 1

 0
 95

 0
 106

 0
 11
 2

 0
 84

 0
 21

 0
 75
 15

 0
 35
 10

 0
 3

 0
 21
 25

 0
 91

 0
 18
 2

 0
 30

 0
 44

 0
 7

 0
 381
 8

 0
 4

 0
 57
 26

 0
 5
 3

 0
 1

 0
 1

 0
 31
 1

 0
 1
 1

 0
 2

 0
 6

 0
 4
 1

 0
 41
 1

 0
 6

 0
 1

 0
 4

 0
 85

 0
 28
 20

 0
 5

 0
 1

 0
 2

 0
 7

 0
 2

 0
 1

 0
 1

 0
 5

 0
 2

 0
 1

 0
 1

 0
 14

 0
 3

 0
 2

 0
 2

 0
 8
 1

 0
 21

 0
 27

 0
 4

 0
 3

 0
 1

 0
 1

 0
 1

 0
 1

 0
 1

 0
 5

 0
 1

 0
 2

 0
 1

 0
 1

 0
 4

 0
 1

 0
 1

 0
 2

 0
 1

 0
 1

 0
 1

 0
 33

 0
 22

 0
 16

 0
 1

 0
 1

 0
 1

 0
 1

 0
 1

 0
 2

 0
 1

 0
 1

 0
 4

 0
 0
 2

 0
 0
 1

 0
 0
 1

 0
 0
 11
 1

 0
 0
 10
 4

 0
 0
 11

 0
 0
 2

 0
 0
 1

 0
 0
 7

 0
 0
 12
 1

 0
 0
 8

 0
 0
 3

 0
 0
 2
 1

 0
 0
 8

 0
 0
 11

 0
 0
 1

 0
 0
 3

 0
 0
 2

 0
 0
 1

 0
 0
 0
 2

 0
 0
 0
 5

 0
 0
 0
 2

 0
 0
 0
 2

 0
 0
 0
 4

 0
 0
 0
 3

 0
 0
 0
 1

 0
 0
 0
 2

 0
 0
 0
 2

 0
 0
 0
 3

 0
 0
 0
 1

 0
 0
 0
 1

 0
 0
 0
 1

 0
 0
 0
 3

 0
 0
 0
 1

 0
 0
 0
 2

 0
 0
 0
 10

 0
 0
 0
 5

 0
 0
 0
 1

 0
 0
 0
 2

 0
 0
 0
 4

 0
 0
 0
 1

 0
 0
 0
 1

 0
 0
 0
 11

 0
 0
 0
 1

 0
 0
 0
 2

 0
 0
 0
 1

 0
 0
 0
 75

 0
 0
 0
 3

 0
 0
 0
 2

 0
 0
 0
 1

 0
 0
 0
 597

 0
 0
 0
 3

 0
 0
 0
 21

 0
 0
 0
 13

 0
 0
 0
 1

 0
 0
 0
 2

 0
 0
 0
 3

 0
 0
 0
 216

 0
 0
 0
 67

 0
 0
 0
 1

 0
 0
 0
 41

 0
 0
 0
 1

 0
 0
 0
 1

 0
 0
 0
 13

 0
 0
 0
 1

 0
 0
 0
 1

 0
 0
 0
 1

 0
 0
 0
 1

 0
 0
 0
 273

 0
 0
 0
 8

 12
 38
 18
 675

 4
 0
 2

 4

 1
 0
 4
 403

 1

 1
 0
 0
 56

 1

 0
 9

 0
 1
 1

 0
 6
 2

 0
 18
 6

 0
 4

 0
 0
 1

 0
 0
 1

 0
 0
 1

 0
 0
 0
 2

 0
 0
 0
 83

 0
 0
 0
 25

 0
 0
 0
 106

 2
 0
 1

 2
 0
 1

 1

 1

 0
 0
 1

 31
 0
 5
 201

 30
 0
 5
 201

 1

 1

 7
 0
 1

 1

 2

 1

 4

 2
 0
 1

 2
 0
 1

 1

 3

 2

 2

 1

 0
 0
 1

 0
 0
 1

 0
 0
 0
 1

 0
 0
 0
 1

 0
 0
 0
 8

 0
 0
 0
 2

 0
 0
 0
 16

 0
 0
 0
 1

 0
 0
 0
 41

 0
 0
 0
 4

 0
 0
 0
 66

 0
 0
 0
 58

 0
 0
 0
 1

 0
 0
 0
 2

 1

 1

 0
 4

 0
 4

 0
 4

 1393
 0
 101

 1393
 0
 101

 20
 0
 3

 20
 0
 3

 1
 0
 2

 1

 1

 4

 2

 3

 3
 0
 1

 1

 2

 1

 1

 15
 0
 3

 13

 13

 2
 0
 3

 1

 1

 0
 0
 1

 0
 0
 2

 1262
 0
 89

 51
 0
 1

 1

 1

 2

 1

 4

 1

 2

 1

 2

 5

 12

 2

 3

 3

 2

 2

 1

 2

 1

 2

 1

 0
 0
 1

 2

 2

 15
 0
 3

 1

 5
 0
 2

 8
 0
 1

 1

 46
 0
 4

 1

 2

 1

 1

 1

 1

 1

 1

 2

 2

 1
 0
 1

 1

 1

 3

 1

 3

 1

 8

 1

 2

 2

 1

 1

 1

 1

 2

 2

 1

 0
 0
 3

 211
 0
 9

 1

 1

 2

 1

 1

 1

 2

 4

 1

 1

 22

 11

 16
 0
 1

 5

 13
 0
 1

 61
 0
 1

 1

 3

 4

 2

 5
 0
 1

 1

 25
 0
 1

 1

 5
 0
 2

 4

 2

 1

 3

 3

 2
 0
 1

 1

 1

 1

 2

 1

 0
 0
 1

 7

 1

 1

 3

 2

 26
 0
 3

 1

 6

 1

 14
 0
 2

 3

 1

 0
 0
 1

 2

 1

 1

 605
 0
 51

 1

 1

 1

 1

 1

 1

 1

 1

 1

 1

 1

 1

 1

 1

 1

 1

 1

 1

 1

 1

 1

 1

 1

 1

 3

 2

 1

 2

 1

 2

 1

 1

 1

 1

 1

 5

 1

 2

 2

 1

 1

 1

 1

 1

 1

 4

 1

 1

 2

 1

 1

 6

 1

 2

 1

 1

 6

 1

 1

 2
 0
 2

 1

 1

 2

 2

 2

 1

 3
 0
 1

 3

 11

 1

 2

 1

 3

 1

 5

 4
 0
 2

 1

 10

 5
 0
 2

 92
 0
 3

 7

 30
 0
 2

 73

 1

 14

 2
 0
 1

 1

 11
 0
 1

 2
 0
 1

 2

 6

 4

 5

 1

 4

 8

 3

 2

 4
 0
 1

 1

 9

 2

 2

 7

 2

 1

 2

 42
 0
 1

 2

 5

 22

 12

 4
 0
 1

 3

 1

 2

 1

 1

 1

 1

 6

 1

 2

 2

 2

 2

 3

 1

 3

 2

 1

 1

 1

 5

 1

 1

 1

 1

 1

 1

 1

 1

 1

 1

 1

 1

 1

 1

 1

 1

 1

 1

 1

 1

 1

 1

 1

 1

 1

 1

 1

 1

 1

 1

 1

 1

 0
 0
 1

 0
 0
 1

 0
 0
 4

 0
 0
 1

 0
 0
 1

 0
 0
 1

 0
 0
 1

 0
 0
 1

 0
 0
 1

 0
 0
 1

 0
 0
 1

 0
 0
 1

 0
 0
 3

 0
 0
 3

 0
 0
 5

 0
 0
 1

 0
 0
 1

 0
 0
 1

 0
 0
 1

 0
 0
 1

 0
 0
 2

 7
 0
 1

 1

 2

 1

 1

 1

 1

 0
 0
 1

 2
 0
 1

 2

 0
 0
 1

 7

 1

 1

 2

 3

 5

 2

 1

 1

 1

 1

 1

 1

 1

 273
 0
 14

 1

 1

 1

 1

 1

 1

 1

 3

 2

 2

 7

 5

 3

 4

 3

 3

 2

 2

 2

 3

 1

 1

 5

 3

 7

 12
 0
 3

 28

 1

 4

 3

 3

 1

 1

 14
 0
 2

 12
 0
 2

 1

 6

 4

 2

 1
 0
 3

 13

 4

 2

 1

 1

 3

 1

 2

 4
 0
 1

 3

 1

 2

 5

 5

 6

 4

 2

 1

 5

 5

 1

 2

 2

 3

 11
 0
 2

 1

 5

 1

 2

 1

 2

 1

 1

 3

 1

 1

 2

 1

 1

 1

 1

 1

 0
 0
 1

 1
 0
 2

 1

 0
 0
 2

 90
 0
 6

 90
 0
 6

 1

 1

 1

 1

 1

 1

 1

 1

 1

 2

 1

 4

 2

 2

 2

 1

 1
 0
 1

 1

 1

 10
 0
 1

 2

 1

 6

 3

 1

 1

 1
 0
 1

 4

 3

 3

 2

 1

 1

 1

 1

 3

 1

 1

 2

 2

 1

 4

 1

 1

 2

 1

 1

 1

 1

 1

 0
 0
 1

 0
 0
 1

 0
 0
 1

 6

 6

 1

 1

 1

 1

 1

 1

 645
 1542
 247

 645
 1542
 247

 12
 0
 3

 2
 0
 1

 1
 0
 1

 1

 1

 1

 5

 1

 1

 1

 1

 1

 4
 0
 2

 1

 1

 1

 1

 0
 0
 1

 0
 0
 1

 632
 1542
 244

 120
 0
 1

 1

 1

 1

 1

 1

 4

 5

 96

 3

 4

 1

 1

 1

 0
 0
 1

 1
 1362
 81

 1
 657
 65

 0
 1

 0
 1

 0
 1

 0
 2

 0
 4

 0
 1

 0
 1

 0
 1

 0
 1

 0
 2

 0
 1

 0
 3

 0
 1

 0
 1

 0
 1

 0
 1

 0
 1

 0
 13

 0
 1

 0
 1

 0
 1

 0
 2

 0
 8

 0
 1

 0
 1

 0
 77
 3

 0
 139
 2

 0
 21

 0
 8

 0
 164
 2

 0
 2

 0
 38
 3

 0
 46
 1

 0
 2

 0
 20

 0
 17

 0
 1

 0
 2

 0
 51

 0
 5

 0
 5

 0
 3

 0
 2

 0
 13

 0
 2

 0
 3

 0
 1

 0
 2

 0
 2

 0
 1

 0
 2

 0
 1

 0
 1

 0
 4

 0
 2

 0
 2

 0
 1

 0
 1

 0
 1

 0
 8

 0
 1

 0
 1

 0
 1

 0
 0
 1

 0
 0
 2

 0
 0
 1

 0
 0
 1

 1

 1

 90
 0
 72

 1

 1

 1

 1

 1

 1

 1

 1

 3

 7

 7

 2

 5

 2

 2

 4

 1

 18
 0
 3

 1
 0
 1

 5
 0
 3

 6
 0
 6

 1
 0
 7

 3

 1

 1

 1

 1

 1

 1

 4

 1

 1

 1

 1

 1

 0
 0
 1

 0
 0
 1

 0
 0
 13

 0
 0
 8

 0
 0
 29

 7
 0
 2

 7
 0
 1

 0
 0
 1

 55
 180
 29

 3

 1

 1
 0
 2

 1

 1

 1

 1

 1

 1

 7

 1

 3

 2

 14
 0
 2

 1

 1

 1

 1

 2

 1

 1

 1

 1

 1
 0
 1

 1

 1

 2

 2

 0
 1

 0
 1

 0
 2

 0
 1

 0
 14
 1

 0
 5
 1

 0
 6
 1

 0
 2

 0
 1

 0
 1

 0
 26
 2

 0
 51
 1

 0
 48
 2

 0
 19
 2

 0
 1

 0
 1

 0
 0
 1

 0
 0
 1

 0
 0
 1

 0
 0
 1

 0
 0
 1

 0
 0
 2

 0
 0
 5

 0
 0
 1

 0
 0
 1

 310
 0
 56

 1

 1

 1

 1

 1

 1

 1

 1

 1

 1

 2

 1

 1

 1

 1

 3

 1

 1

 1
 0
 4

 1

 9

 1

 1

 1

 7

 1

 1

 1

 1

 10

 2

 2

 3

 1

 1

 12
 0
 7

 8
 0
 1

 95
 0
 5

 3

 2

 1

 2

 1

 7
 0
 4

 4
 0
 1

 2

 16

 2

 3
 0
 4

 11
 0
 3

 3

 4
 0
 1

 3

 1

 1

 1

 2

 1

 7
 0
 2

 1

 1

 1

 2

 3

 1

 2

 3

 1

 1

 1

 1

 2

 1

 1

 1

 1

 1

 1

 2

 1

 1

 1

 2

 1

 2

 1

 1

 1

 1

 1

 1

 1

 1

 1

 1

 1

 1

 2

 1

 1

 1

 0
 0
 1

 0
 0
 2

 0
 0
 1

 0
 0
 2

 0
 0
 1

 0
 0
 3

 0
 0
 2

 0
 0
 1

 0
 0
 1

 0
 0
 1

 0
 0
 2

 0
 0
 1

 0
 0
 1

 0
 0
 1

 0
 0
 4

 18

 18

 13
 0
 1

 1

 12

 0
 0
 1

 2

 1

 1

 1

 1

 13
 0
 1

 1

 1

 1

 5

 1
 0
 1

 1

 1

 1

 1

 1

 1

 0
 0
 1

 0
 0
 1

 1

 1

 1

 1646
 0
 204

 1646
 0
 204

 796
 0
 51

 4

 2

 1

 1

 4
 0
 1

 1

 2

 1

 0
 0
 1

 516
 0
 24

 1

 1

 1

 1

 1

 1

 1

 1

 1

 1

 1

 1

 1

 1

 1

 1

 1

 1

 1

 1

 1

 1

 1

 1

 1

 1

 3

 1

 1

 1

 2

 1

 2

 1

 1

 1

 2

 6
 0
 2

 1

 1
 0
 1

 1

 1

 1

 1

 2

 1

 1

 1

 1

 1

 1

 1

 3

 1

 1

 5

 3

 2

 1

 1

 2

 1

 1

 3

 4

 1

 7

 2

 1

 1

 1

 1

 3

 1

 1

 2

 1

 1

 1

 1

 1

 1

 1

 3

 4

 4

 6

 2

 1

 1

 1

 2

 1

 3

 2

 3

 3

 2
 0
 1

 3

 12

 2

 2

 1

 19

 2

 5

 2

 1

 2

 1

 15

 4

 6

 2

 1

 2

 3

 8

 1

 1

 2

 4

 9

 1

 1

 33
 0
 2

 7

 5

 1

 1

 3

 2

 19

 4

 1

 1

 5

 1

 1

 5

 10
 0
 1

 6

 2

 2

 22

 2

 3

 2

 4

 1

 1

 1

 1

 2

 2

 1
 0
 1

 1

 1

 1

 1

 1

 2

 2

 1

 1

 1

 1

 2

 1

 1
 0
 1

 1

 1

 3

 1

 1

 4

 2

 1

 1

 1

 1
 0
 1

 1

 1

 1

 1

 6
 0
 1

 2

 1

 3

 1

 3

 1
 0
 1

 2

 1

 1

 1

 1

 1

 1

 2

 1

 1

 1

 1

 1

 1

 1

 1

 1

 1

 1

 1

 1

 1

 2

 1

 1

 1

 1

 1

 1

 1

 1

 1

 1

 1

 2

 1

 1

 0
 0
 1

 0
 0
 1

 0
 0
 1

 0
 0
 1

 0
 0
 1

 0
 0
 1

 0
 0
 1

 0
 0
 1

 0
 0
 1

 0
 0
 1

 0
 0
 1

 0
 0
 1

 74
 0
 7

 1

 1

 1

 1

 1

 1

 2

 1

 1

 1

 3

 1

 1

 2

 1

 16
 0
 1

 3

 1

 1

 1

 1

 6

 1

 1

 1
 0
 1

 3
 0
 2

 1

 1

 3

 1

 4

 1

 1

 1

 1

 1

 1

 1

 1

 1

 1

 0
 0
 1

 0
 0
 1

 0
 0
 1

 56
 0
 2

 1

 1

 1

 1

 2

 43
 0
 1

 2
 0
 1

 1

 1

 1

 1

 1

 4

 1

 1

 1

 1

 30
 0
 1

 1

 4

 2

 2

 2

 5
 0
 1

 1

 2

 2

 4

 5

 2
 0
 1

 1

 1
 0
 1

 10
 0
 1

 2

 1

 1

 1

 2

 3
 0
 1

 54
 0
 5

 1

 1

 1

 1

 1

 2

 1

 1

 6

 3

 12
 0
 1

 1

 1

 1

 6

 1

 2
 0
 2

 4

 1

 2

 1

 1

 1

 1

 1

 0
 0
 1

 0
 0
 1

 5
 0
 4

 1

 1

 2
 0
 1

 1

 0
 0
 2

 0
 0
 1

 37
 0
 4

 1

 5

 1

 22
 0
 1

 2

 1
 0
 1

 1

 1

 1

 1

 1

 0
 0
 1

 0
 0
 1

 0
 0
 1

 0
 0
 1

 23
 0
 7

 23
 0
 1

 4

 1

 1

 1

 11
 0
 1

 4

 1

 0
 0
 6

 0
 0
 1

 0
 0
 1

 0
 0
 2

 0
 0
 2

 14
 0
 1

 8

 1

 1

 3

 1

 1

 1

 1

 1

 5
 0
 1

 1

 1

 1

 1

 1

 0
 0
 1

 804
 0
 142

 2
 0
 1

 2
 0
 1

 191
 0
 13

 1

 1

 1

 1

 1

 1

 1

 1

 1

 1

 1

 1

 1

 1

 1

 1

 1

 1

 1

 1

 1

 1

 6

 1

 1

 2

 1

 4

 2

 5

 1

 21
 0
 2

 4

 1

 1

 13
 0
 1

 4

 3

 4

 4

 10
 0
 1

 16

 1

 1

 2

 9

 9

 6

 4

 2

 2

 3
 0
 1

 1

 3

 1

 2

 1
 0
 1

 1

 1

 2

 2

 1

 2

 1

 1

 1

 1

 1

 1

 1

 1

 1

 0
 0
 1

 0
 0
 1

 0
 0
 2

 0
 0
 2

 0
 0
 1

 1
 0
 1

 1

 0
 0
 1

 2

 1

 1

 466
 0
 69

 1

 1

 1

 1

 1

 1

 2

 1

 1

 1

 1

 2

 2
 0
 2

 3
 0
 1

 1

 4

 1

 1

 2

 3

 2

 1

 41
 0
 2

 5

 3

 22

 17
 0
 3

 1

 4

 20
 0
 6

 27
 0
 2

 3
 0
 1

 14

 3

 5
 0
 2

 2

 57
 0
 12

 1
 0
 2

 4

 1

 4

 5
 0
 1

 119
 0
 7

 1

 1

 5

 11

 15

 2

 5

 1
 0
 2

 10

 1

 4

 1

 1

 2

 3

 4

 1

 1

 1

 1

 1

 1

 1

 0
 0
 2

 0
 0
 3

 0
 0
 1

 0
 0
 1

 0
 0
 1

 0
 0
 7

 0
 0
 6

 0
 0
 1

 0
 0
 1

 0
 0
 1

 0
 0
 2

 2

 2

 73
 0
 21

 1

 1

 1

 1

 1

 1

 2

 1

 2

 3

 6

 3

 1

 1

 1

 1

 4

 3

 1

 4
 0
 1

 2

 12

 1

 5
 0
 2

 1
 0
 6

 1

 1

 1
 0
 2

 1

 1

 1

 1

 1

 1

 1

 1

 2

 0
 0
 1

 0
 0
 1

 0
 0
 3

 0
 0
 1

 0
 0
 1

 0
 0
 2

 0
 0
 1

 67
 0
 37

 1

 1

 1

 1

 1

 1

 2

 7
 0
 2

 1

 38
 0
 19

 1
 0
 3

 2

 1

 1

 1

 1

 1

 1

 1

 2

 1

 0
 0
 1

 0
 0
 1

 0
 0
 1

 0
 0
 2

 0
 0
 1

 0
 0
 3

 0
 0
 3

 0
 0
 1

 9
 0
 3

 9
 0
 3

 1

 1

 1

 1

 1

 1

 2

 1

 0
 0
 2

 0
 0
 1

 0
 1

 0
 1

 0
 1

 0
 1

 0
 1

 53
 0
 5

 53
 0
 5

 53
 0
 5

 53
 0
 5

 53
 0
 5

 1

 1

 1

 2

 1

 1

 1

 1

 1

 1

 1

 1

 2

 1

 1

 1

 1

 1

 2

 2

 1

 1

 1

 2

 1
 0
 1

 1

 1

 1

 1

 1

 2

 1

 1

 1

 1

 1

 1

 1

 1

 1

 2

 1

 1

 1

 1

 1

 0
 0
 1

 0
 0
 1

 0
 0
 1

 0
 0
 1

 8
 0
 3

 8
 0
 3

 8
 0
 3

 8
 0
 3

 8
 0
 3

 1

 1

 2
 0
 1

 1

 1

 1

 1

 0
 0
 1

 0
 0
 1

 15
 0
 7

 15
 0
 7

 15
 0
 7

 10
 0
 1

 10
 0
 1

 7

 1

 2

 0
 0
 1

 5
 0
 6

 2
 0
 1

 1
 0
 1

 1

 3
 0
 4

 1

 1

 1

 0
 0
 1

 0
 0
 2

 0
 0
 1

 0
 0
 1

 0
 0
 1

 197
 0
 55

 56
 0
 6

 56
 0
 6

 56
 0
 6

 55
 0
 6

 1

 2

 1

 1

 2

 1

 1

 1

 1

 3

 1

 1

 2

 1

 1

 1

 1

 6

 2

 3

 2

 2

 1

 2

 3

 1

 1

 1

 6

 1

 1

 1

 0
 0
 1

 0
 0
 1

 0
 0
 1

 0
 0
 2

 0
 0
 1

 1

 1

 10
 0
 3

 10
 0
 3

 10
 0
 3

 4
 0
 2

 1

 1

 1

 1

 0
 0
 1

 0
 0
 1

 6
 0
 1

 1

 2

 1

 1

 1

 0
 0
 1

 52
 0
 12

 52
 0
 12

 52
 0
 12

 52
 0
 12

 1

 1

 1

 1

 1

 1

 1

 1

 1

 1

 1

 1

 1

 1

 2

 3

 1

 1

 2

 4

 1

 1

 1

 2

 2

 1

 2

 1

 1

 1

 1

 2

 1

 1

 1

 3

 1

 1

 1

 0
 0
 1

 0
 0
 1

 0
 0
 5

 0
 0
 1

 0
 0
 2

 0
 0
 2

 10
 0
 1

 4

 2

 2

 1

 1

 2

 2

 1

 1

 4

 4

 4

 1

 2

 1

 2
 0
 1

 2
 0
 1

 2
 0
 1

 2

 0
 0
 1

 33
 0
 13

 33
 0
 13

 8
 0
 9

 8
 0
 9

 1

 1

 2

 2

 1

 1

 0
 0
 2

 0
 0
 2

 0
 0
 1

 0
 0
 1

 0
 0
 1

 0
 0
 1

 0
 0
 1

 25
 0
 4

 25
 0
 4

 1

 2

 9

 1

 1
 0
 1

 2

 1

 5

 1

 1

 1

 0
 0
 1

 0
 0
 1

 0
 0
 1

 36
 0
 20

 29
 0
 18

 29
 0
 18

 29
 0
 18

 1

 1

 1

 2

 3
 0
 2

 2

 1

 12
 0
 1

 1

 1

 1

 1

 1

 1

 0
 0
 1

 0
 0
 1

 0
 0
 4

 0
 0
 1

 0
 0
 5

 0
 0
 1

 0
 0
 1

 0
 0
 1

 7
 0
 2

 7
 0
 2

 7
 0
 2

 2

 5
 0
 1

 0
 0
 1

 1

 1

 1

 1

 1

 1

 176
 0
 106

 176
 0
 106

 175
 0
 83

 151
 0
 79

 151
 0
 79

 1

 1

 3

 1

 1

 1

 1

 1

 3

 1

 4

 3

 6
 0
 1

 17
 0
 1

 5
 0
 4

 2

 11

 18

 16
 0
 1

 7

 1
 0
 21

 1
 0
 6

 7

 7

 1
 0
 4

 1
 0
 13

 3

 2
 0
 1

 1

 8

 1

 4
 0
 1

 2
 0
 4

 3

 3

 1

 1

 1

 0
 0
 3

 0
 0
 1

 0
 0
 2

 0
 0
 3

 0
 0
 1

 0
 0
 2

 0
 0
 2

 0
 0
 1

 0
 0
 1

 0
 0
 1

 0
 0
 2

 0
 0
 1

 0
 0
 2

 24
 0
 4

 24
 0
 4

 3

 1

 5
 0
 2

 1

 5

 2

 3

 1

 1

 1

 1

 0
 0
 2

 1
 0
 23

 1
 0
 23

 1
 0
 23

 1

 0
 0
 17

 0
 0
 1

 0
 0
 4

 0
 0
 1

 1
 0
 1

 1
 0
 1

 1
 0
 1

 1
 0
 1

 1
 0
 1

 1

 0
 0
 1

 1
 0
 1

 1
 0
 1

 1
 0
 1

 1
 0
 1

 1
 0
 1

 1
 0
 1

 1490
 3553
 5065
 9997

 765
 832
 1951
 14

 349
 22
 691

 4
 0
 28

 1
 0
 4

 1

 0
 0
 4

 3
 0
 8

 1

 1

 1

 0
 0
 1

 0
 0
 1

 0
 0
 1

 0
 0
 1

 0
 0
 3

 0
 0
 1

 0
 0
 2

 0
 0
 1

 0
 0
 1

 0
 0
 14

 0
 0
 1

 0
 0
 3

 0
 0
 5

 0
 0
 1

 0
 0
 1

 0
 0
 1

 0
 0
 1

 0
 0
 1

 9
 1
 38

 1
 0
 7

 1

 0
 0
 1

 0
 0
 1

 0
 0
 2

 0
 0
 1

 0
 0
 1

 0
 0
 1

 1
 0
 7

 1

 0
 0
 1

 0
 0
 1

 0
 0
 1

 0
 0
 1

 0
 0
 1

 0
 0
 1

 0
 0
 1

 6
 1
 20

 1

 1
 1
 5

 4
 0
 3

 0
 0
 1

 0
 0
 1

 0
 0
 1

 0
 0
 1

 0
 0
 8

 1
 0
 2

 1

 0
 0
 2

 0
 0
 2

 0
 0
 2

 32
 0
 30

 25
 0
 15

 1

 1

 6

 8

 4
 0
 1

 2

 1

 1

 1

 0
 0
 2

 0
 0
 2

 0
 0
 1

 0
 0
 4

 0
 0
 2

 0
 0
 2

 0
 0
 1

 1
 0
 7

 1
 0
 7

 2
 0
 2

 1

 1

 0
 0
 2

 4
 0
 1

 3

 1

 0
 0
 1

 0
 0
 1

 0
 0
 1

 0
 0
 3

 0
 0
 3

 0
 0
 1

 0
 0
 1

 1
 0
 90

 1
 0
 90

 1

 0
 0
 90

 54
 0
 53

 54
 0
 53

 1

 1

 1
 0
 1

 1

 1

 1

 1

 1

 24

 1
 0
 1

 1

 4
 0
 1

 1

 1

 2
 0
 4

 1

 1

 6

 2

 1

 1

 0
 0
 1

 0
 0
 1

 0
 0
 1

 0
 0
 1

 0
 0
 1

 0
 0
 1

 0
 0
 1

 0
 0
 1

 0
 0
 2

 0
 0
 1

 0
 0
 5

 0
 0
 1

 0
 0
 4

 0
 0
 1

 0
 0
 1

 0
 0
 3

 0
 0
 1

 0
 0
 1

 0
 0
 2

 0
 0
 4

 0
 0
 2

 0
 0
 1

 0
 0
 1

 0
 0
 1

 0
 0
 1

 0
 0
 1

 0
 0
 2

 0
 0
 2

 0
 0
 1

 4
 0
 87

 4
 0
 86

 4
 0
 86

 0
 0
 1

 0
 0
 1

 7
 0
 80

 2
 0
 17

 1

 1

 0
 0
 1

 0
 0
 1

 0
 0
 1

 0
 0
 2

 0
 0
 1

 0
 0
 3

 0
 0
 2

 0
 0
 1

 0
 0
 1

 0
 0
 2

 0
 0
 1

 0
 0
 1

 5
 0
 44

 1

 1
 0
 9

 1
 0
 1

 1

 1

 0
 0
 3

 0
 0
 1

 0
 0
 1

 0
 0
 2

 0
 0
 1

 0
 0
 2

 0
 0
 1

 0
 0
 1

 0
 0
 2

 0
 0
 1

 0
 0
 1

 0
 0
 1

 0
 0
 2

 0
 0
 1

 0
 0
 1

 0
 0
 3

 0
 0
 2

 0
 0
 2

 0
 0
 1

 0
 0
 1

 0
 0
 1

 0
 0
 1

 0
 0
 1

 0
 0
 1

 0
 0
 11

 0
 0
 2

 0
 0
 2

 0
 0
 2

 0
 0
 4

 0
 0
 1

 0
 0
 8

 0
 0
 2

 0
 0
 1

 0
 0
 1

 0
 0
 3

 0
 0
 1

 30
 0
 101

 6
 0
 5

 6
 0
 2

 0
 0
 3

 3
 0
 42

 1

 1
 0
 6

 1

 0
 0
 1

 0
 0
 6

 0
 0
 1

 0
 0
 28

 19
 0
 29

 1

 1

 1

 1

 1

 1
 0
 3

 5
 0
 1

 3
 0
 1

 3
 0
 1

 1

 1

 0
 0
 1

 0
 0
 1

 0
 0
 1

 0
 0
 8

 0
 0
 3

 0
 0
 1

 0
 0
 4

 0
 0
 2

 0
 0
 1

 0
 0
 1

 2
 0
 13

 2
 0
 1

 0
 0
 1

 0
 0
 2

 0
 0
 1

 0
 0
 8

 0
 0
 8

 0
 0
 8

 0
 0
 1

 0
 0
 1

 0
 0
 1

 0
 0
 1

 0
 0
 2

 0
 0
 2

 205
 0
 114

 25
 0
 27

 1

 3
 0
 2

 1
 0
 1

 1

 15
 0
 24

 3

 1

 7
 0
 24

 3
 0
 3

 3
 0
 14

 1
 0
 7

 5
 0
 7

 5
 0
 4

 0
 0
 3

 2
 0
 6

 1

 1

 0
 0
 1

 0
 0
 1

 0
 0
 3

 0
 0
 1

 166
 0
 50

 1

 1

 1
 0
 1

 1
 0
 1

 1
 0
 2

 1

 4
 0
 2

 148
 0
 10

 1

 1

 1

 1

 2

 1

 1

 0
 0
 1

 0
 0
 1

 0
 0
 1

 0
 0
 2

 0
 0
 2

 0
 0
 7

 0
 0
 4

 0
 0
 2

 0
 0
 4

 0
 0
 1

 0
 0
 3

 0
 0
 2

 0
 0
 1

 0
 0
 1

 0
 0
 1

 0
 0
 1

 2
 0
 11

 1
 0
 1

 1

 0
 0
 1

 1
 0
 5

 1
 0
 1

 0
 0
 1

 0
 0
 1

 0
 0
 1

 0
 0
 1

 0
 0
 1

 0
 0
 1

 0
 0
 4

 0
 0
 1

 0
 0
 3

 1
 0
 1

 1
 0
 1

 1

 0
 0
 1

 0
 21
 30

 0
 21
 30

 0
 1

 0
 1

 0
 1

 0
 1

 0
 6

 0
 3

 0
 2

 0
 1

 0
 1

 0
 1

 0
 2

 0
 1

 0
 0
 4

 0
 0
 25

 0
 0
 1

 0
 0
 13

 0
 0
 13

 0
 0
 1

 0
 0
 2

 0
 0
 2

 0
 0
 1

 0
 0
 1

 0
 0
 2

 0
 0
 1

 0
 0
 1

 0
 0
 1

 0
 0
 1

 0
 0
 4

 0
 0
 4

 0
 0
 4

 0
 0
 11

 0
 0
 11

 0
 0
 11

 6
 2
 1

 6
 2
 1

 6
 2
 1

 1
 0
 1

 5

 0
 2

 410
 808
 1259
 14

 26
 9
 118

 9
 0
 15

 1

 4

 1
 0
 3

 1

 2

 0
 0
 7

 0
 0
 3

 0
 0
 2

 2
 0
 2

 1

 1

 0
 0
 1

 0
 0
 1

 1
 0
 1

 1
 0
 1

 13
 0
 100

 1

 10
 0
 4

 2
 0
 7

 0
 0
 1

 0
 0
 42

 0
 0
 22

 0
 0
 7

 0
 0
 17

 1

 1

 0
 9

 0
 1

 0
 7

 0
 1

 131
 8
 48

 3

 1

 2

 22
 0
 13

 9
 0
 4

 5
 0
 8

 1

 4

 2

 1

 0
 0
 1

 11
 2

 1

 9

 1

 0
 2

 16
 0
 6

 1
 0
 6

 15

 75

 4

 71

 2

 1

 1

 1
 6
 2

 1

 0
 1

 0
 3
 1

 0
 2

 0
 0
 1

 1
 0
 2

 1

 0
 0
 2

 0
 0
 25

 0
 0
 25

 22
 14
 20

 10
 11
 11

 9
 0
 10

 1

 0
 11

 0
 0
 1

 9
 3
 6

 3

 5
 0
 6

 1

 0
 3

 3
 0
 3

 1

 1

 1
 0
 1

 0
 0
 2

 94
 18
 225
 10

 93
 18
 213
 10

 1

 1

 1

 1

 3

 1
 0
 3

 1

 39
 0
 6

 3
 3
 17

 6
 0
 2

 4

 3

 2
 1
 1
 2

 1

 1

 3
 0
 1

 1

 6
 0
 44

 1
 0
 4

 3
 0
 4

 1

 4

 2
 0
 3

 1

 1

 1

 1

 0
 10

 0
 1

 0
 1
 9
 1

 0
 2

 0
 0
 1

 0
 0
 1

 0
 0
 1

 0
 0
 2

 0
 0
 1

 0
 0
 1

 0
 0
 5

 0
 0
 5

 0
 0
 1

 0
 0
 2

 0
 0
 1

 0
 0
 1

 0
 0
 57

 0
 0
 20

 0
 0
 1

 0
 0
 4

 0
 0
 3

 0
 0
 12

 0
 0
 0
 1

 0
 0
 0
 1

 0
 0
 0
 2

 0
 0
 0
 2

 0
 0
 0
 1

 1
 0
 10

 1
 0
 3

 0
 0
 1

 0
 0
 1

 0
 0
 5

 0
 0
 2

 0
 0
 2

 41
 10
 19

 41
 10
 19

 1

 2

 1

 2
 0
 1

 4
 0
 3

 5

 15
 0
 9

 10

 1

 0
 1

 0
 6

 0
 1

 0
 2

 0
 0
 4

 0
 0
 1

 0
 0
 1

 40
 0
 703

 17
 0
 340

 1
 0
 38

 2
 0
 4

 13
 0
 142

 1
 0
 20

 0
 0
 1

 0
 0
 1

 0
 0
 2

 0
 0
 64

 0
 0
 56

 0
 0
 9

 0
 0
 2

 0
 0
 1

 2

 2

 21
 0
 363

 1

 1
 0
 1

 2
 0
 1

 16

 1

 0
 0
 2

 0
 0
 3

 0
 0
 337

 0
 0
 14

 0
 0
 1

 0
 0
 4

 56
 749
 126
 4

 22
 0
 12
 1

 1

 1

 13
 0
 6
 1

 2
 0
 1

 2

 1

 1

 1

 0
 0
 5

 34
 749
 114
 3

 1

 1

 1

 1

 1

 1

 12
 0
 8

 9
 4
 5

 1
 0
 4

 1

 1

 1

 1

 1

 1

 0
 1

 0
 1

 0
 1

 0
 1

 0
 1

 0
 2

 0
 2

 0
 1

 0
 1

 0
 1

 0
 1

 0
 1

 0
 1

 0
 1

 0
 4

 0
 1

 0
 2

 0
 1

 0
 1

 0
 2

 0
 1

 0
 1

 0
 1

 0
 1

 0
 1

 0
 2

 0
 1

 0
 1

 0
 1

 0
 3

 0
 1

 0
 1

 0
 1

 0
 1

 0
 2

 0
 1

 0
 3

 0
 4
 1

 0
 2

 0
 1
 1

 0
 21
 1

 0
 10
 6

 0
 269
 2

 0
 205
 3

 0
 6
 3

 0
 21

 0
 1

 0
 1

 0
 6

 0
 41
 43

 0
 1

 0
 9

 0
 2

 0
 1
 1

 0
 20

 0
 2

 0
 3

 0
 1

 0
 2

 0
 23

 0
 1

 0
 1

 0
 3

 0
 6

 0
 1

 0
 1

 0
 1

 0
 1

 0
 1

 0
 1

 0
 1

 0
 1

 0
 2

 0
 1

 0
 1

 0
 1

 0
 1

 0
 1

 0
 2

 0
 1

 0
 2

 0
 1

 0
 1

 0
 1

 0
 1

 0
 1

 0
 1

 0
 1

 0
 1

 0
 2

 0
 1

 0
 1

 0
 0
 1

 0
 0
 1

 0
 0
 1

 0
 0
 1

 0
 0
 1

 0
 0
 3

 0
 0
 1

 0
 0
 1

 0
 0
 1

 0
 0
 1

 0
 0
 1

 0
 0
 1

 0
 0
 2

 0
 0
 9

 0
 0
 1

 0
 0
 2

 0
 0
 1

 0
 0
 2

 0
 0
 1

 0
 0
 1

 0
 0
 1

 0
 0
 1

 0
 0
 1

 0
 0
 0
 1

 0
 0
 0
 1

 0
 0
 0
 1

 640
 1925
 2886
 8278

 5
 1
 11
 115

 5
 1
 11
 115

 5
 1
 11
 115

 2

 1

 1

 1

 0
 1

 0
 0
 1

 0
 0
 1

 0
 0
 2

 0
 0
 1

 0
 0
 1

 0
 0
 1

 0
 0
 2

 0
 0
 1

 0
 0
 1

 0
 0
 0
 3

 0
 0
 0
 5

 0
 0
 0
 14

 0
 0
 0
 40

 0
 0
 0
 2

 0
 0
 0
 1

 0
 0
 0
 9

 0
 0
 0
 1

 0
 0
 0
 5

 0
 0
 0
 21

 0
 0
 0
 2

 0
 0
 0
 12

 635
 1924
 2875
 8163

 104
 2
 104
 7

 8
 0
 8

 1

 1

 3
 0
 3

 1

 1

 1

 0
 0
 1

 0
 0
 1

 0
 0
 1

 0
 0
 1

 0
 0
 1

 93
 2
 94
 7

 1

 1

 1

 3
 0
 3

 13

 48
 0
 53

 17
 0
 1
 2

 1

 1

 1

 1
 0
 1

 1

 1

 1

 2

 0
 2

 0
 0
 1

 0
 0
 1

 0
 0
 1

 0
 0
 5

 0
 0
 3

 0
 0
 1

 0
 0
 1

 0
 0
 2

 0
 0
 13

 0
 0
 1

 0
 0
 1
 2

 0
 0
 2

 0
 0
 1

 0
 0
 2

 0
 0
 1

 0
 0
 0
 1

 0
 0
 0
 1

 0
 0
 0
 1

 1

 1

 2
 0
 1

 2
 0
 1

 0
 0
 1

 0
 0
 1

 23
 102
 1924

 7
 4
 979

 1

 2
 0
 57

 1
 0
 81

 2
 0
 18

 1
 2
 154

 0
 1

 0
 1
 148

 0
 0
 1

 0
 0
 1

 0
 0
 2

 0
 0
 1

 0
 0
 3

 0
 0
 4

 0
 0
 1

 0
 0
 3

 0
 0
 2

 0
 0
 1

 0
 0
 1

 0
 0
 4

 0
 0
 2

 0
 0
 2

 0
 0
 2

 0
 0
 1

 0
 0
 3

 0
 0
 106

 0
 0
 31

 0
 0
 8

 0
 0
 9

 0
 0
 64

 0
 0
 2

 0
 0
 4

 0
 0
 75

 0
 0
 3

 0
 0
 10

 0
 0
 50

 0
 0
 21

 0
 0
 10

 0
 0
 3

 0
 0
 11

 0
 0
 2

 0
 0
 1

 0
 0
 12

 0
 0
 5

 0
 0
 7

 0
 0
 2

 0
 0
 15

 0
 0
 16

 0
 0
 7

 0
 0
 2

 0
 0
 5

 0
 0
 3

 0
 0
 1

 0
 0
 1

 0
 0
 1

 2
 1
 34

 1

 1

 0
 1

 0
 0
 1

 0
 0
 1

 0
 0
 1

 0
 0
 1

 0
 0
 22

 0
 0
 2

 0
 0
 2

 0
 0
 1

 0
 0
 2

 0
 0
 1

 3
 0
 82

 3
 0
 48

 0
 0
 32

 0
 0
 2

 3
 1
 751

 3
 1
 749

 0
 0
 2

 2
 0
 22

 1
 0
 14

 1

 0
 0
 1

 0
 0
 1

 0
 0
 2

 0
 0
 4

 2
 96
 42

 2

 0
 96
 4

 0
 0
 5

 0
 0
 31

 0
 0
 2

 4
 0
 2

 2

 1

 1

 0
 0
 1

 0
 0
 1

 0
 0
 1

 0
 0
 1

 0
 0
 10

 0
 0
 6

 0
 0
 2

 0
 0
 2

 0
 0
 1

 0
 0
 1

 4
 240
 29
 40

 2
 1
 2
 4

 1
 0
 1

 1
 0
 1

 0
 1

 0
 0
 0
 3

 0
 0
 0
 1

 2
 239
 25
 36

 1
 0
 5

 1

 0
 1

 0
 2

 0
 236
 6
 4

 0
 0
 1

 0
 0
 7

 0
 0
 6

 0
 0
 0
 32

 0
 0
 2

 0
 0
 1

 0
 0
 1

 93
 35
 110
 1689

 93
 35
 110
 1689

 1

 1

 1

 1

 1

 1

 1
 0
 0
 4

 1
 0
 2

 1

 2

 1

 1

 3

 1

 1

 1

 2

 1

 1

 1

 1

 1

 1

 1
 0
 5

 3

 1

 1
 0
 1

 3

 1

 1

 1

 2

 1
 0
 1

 1

 17
 0
 2

 1

 1

 1

 2

 1

 1

 1

 1

 1
 0
 1

 1

 2

 1

 1
 0
 0
 72

 1

 1

 1

 1

 1

 1
 0
 1

 1

 1

 1

 1

 1

 1
 0
 0
 2

 2

 1

 1

 1

 1

 0
 1

 0
 2

 0
 1

 0
 1

 0
 1

 0
 1

 0
 4
 1

 0
 5

 0
 2

 0
 2

 0
 1

 0
 1

 0
 7

 0
 2

 0
 2

 0
 1

 0
 1

 0
 0
 1

 0
 0
 1

 0
 0
 1

 0
 0
 1

 0
 0
 1

 0
 0
 1

 0
 0
 3

 0
 0
 1

 0
 0
 1

 0
 0
 1
 46

 0
 0
 2

 0
 0
 1

 0
 0
 1

 0
 0
 1

 0
 0
 1

 0
 0
 1

 0
 0
 1

 0
 0
 1

 0
 0
 1

 0
 0
 1

 0
 0
 1

 0
 0
 1

 0
 0
 1

 0
 0
 3

 0
 0
 2

 0
 0
 5

 0
 0
 5

 0
 0
 1
 28

 0
 0
 1
 1

 0
 0
 11

 0
 0
 1
 155

 0
 0
 6

 0
 0
 1
 2

 0
 0
 1

 0
 0
 1

 0
 0
 3

 0
 0
 2

 0
 0
 4
 21

 0
 0
 3
 44

 0
 0
 1

 0
 0
 1

 0
 0
 1

 0
 0
 1

 0
 0
 1

 0
 0
 1

 0
 0
 1

 0
 0
 1

 0
 0
 1

 0
 0
 1

 0
 0
 2

 0
 0
 1

 0
 0
 1

 0
 0
 1
 7

 0
 0
 1

 0
 0
 1

 0
 0
 1

 0
 0
 1

 0
 0
 1

 0
 0
 0
 1

 0
 0
 0
 1

 0
 0
 0
 4

 0
 0
 0
 2

 0
 0
 0
 1

 0
 0
 0
 1

 0
 0
 0
 3

 0
 0
 0
 1

 0
 0
 0
 10

 0
 0
 0
 1

 0
 0
 0
 1

 0
 0
 0
 1

 0
 0
 0
 3

 0
 0
 0
 1

 0
 0
 0
 2

 0
 0
 0
 1

 0
 0
 0
 2

 0
 0
 0
 2

 0
 0
 0
 1

 0
 0
 0
 15

 0
 0
 0
 1

 0
 0
 0
 2

 0
 0
 0
 3

 0
 0
 0
 1

 0
 0
 0
 1

 0
 0
 0
 1

 0
 0
 0
 1

 0
 0
 0
 1

 0
 0
 0
 1

 0
 0
 0
 1

 0
 0
 0
 2

 0
 0
 0
 5

 0
 0
 0
 6

 0
 0
 0
 1

 0
 0
 0
 2

 0
 0
 0
 6

 0
 0
 0
 6

 0
 0
 0
 1

 0
 0
 0
 1

 0
 0
 0
 2

 0
 0
 0
 4

 0
 0
 0
 26

 0
 0
 0
 16

 0
 0
 0
 6

 0
 0
 0
 18

 0
 0
 0
 1

 0
 0
 0
 2

 0
 0
 0
 1

 0
 0
 0
 8

 0
 0
 0
 8

 0
 0
 0
 3

 0
 0
 0
 3

 0
 0
 0
 14

 0
 0
 0
 2

 0
 0
 0
 2

 0
 0
 0
 7

 0
 0
 0
 2

 0
 0
 0
 4

 0
 0
 0
 6

 0
 0
 0
 2

 0
 0
 0
 14

 0
 0
 0
 6

 0
 0
 0
 8

 0
 0
 0
 1

 0
 0
 0
 16

 0
 0
 0
 1

 0
 0
 0
 3

 0
 0
 0
 5

 0
 0
 0
 6

 0
 0
 0
 3

 0
 0
 0
 1

 0
 0
 0
 5

 0
 0
 0
 6

 0
 0
 0
 1

 0
 0
 0
 1

 0
 0
 0
 1

 0
 0
 0
 4

 0
 0
 0
 11

 0
 0
 0
 12

 0
 0
 0
 4

 0
 0
 0
 1

 0
 0
 0
 3

 0
 0
 0
 29

 0
 0
 0
 212

 0
 0
 0
 10

 0
 0
 0
 5

 0
 0
 0
 51

 0
 0
 0
 1

 0
 0
 0
 3

 0
 0
 0
 6

 0
 0
 0
 17

 0
 0
 0
 65

 0
 0
 0
 2

 0
 0
 0
 3

 0
 0
 0
 2

 0
 0
 0
 1

 0
 0
 0
 4

 0
 0
 0
 6

 0
 0
 0
 4

 0
 0
 0
 3

 0
 0
 0
 4

 0
 0
 0
 4

 0
 0
 0
 4

 0
 0
 0
 7

 0
 0
 0
 4

 0
 0
 0
 5

 0
 0
 0
 1

 0
 0
 0
 9

 0
 0
 0
 274

 0
 0
 0
 3

 0
 0
 0
 1

 0
 0
 0
 1

 0
 0
 0
 2

 0
 0
 0
 11

 0
 0
 0
 2

 0
 0
 0
 64

 0
 0
 0
 51

 0
 0
 0
 1

 0
 0
 0
 1

 0
 0
 0
 1

 0
 0
 0
 1

 0
 0
 0
 2

 0
 0
 0
 7

 0
 0
 0
 1

 0
 0
 0
 4

 0
 0
 0
 2

 0
 0
 0
 1

 0
 0
 0
 3

 0
 0
 0
 4

 0
 0
 0
 1

 0
 0
 0
 1

 0
 0
 0
 3

 0
 0
 0
 20

 0
 0
 0
 1

 0
 0
 0
 1

 0
 0
 0
 2

 0
 0
 0
 1

 0
 0
 0
 5

 0
 0
 0
 1

 0
 0
 0
 1

 0
 0
 0
 2

 0
 0
 0
 2

 0
 0
 0
 3

 0
 0
 0
 4

 0
 0
 0
 1

 0
 0
 0
 1

 0
 0
 0
 1

 0
 0
 0
 5

 0
 0
 0
 3

 0
 0
 0
 2

 1
 0
 3

 1
 0
 3

 1

 0
 0
 1

 0
 0
 2

 143
 620
 230
 3557

 1
 0
 0
 22

 1

 0
 0
 0
 15

 0
 0
 0
 2

 0
 0
 0
 2

 0
 0
 0
 1

 0
 0
 0
 1

 0
 0
 0
 1

 13
 0
 3
 155

 1

 1

 6
 0
 1

 1

 2
 0
 0
 3

 1

 1
 0
 0
 2

 0
 0
 1
 69

 0
 0
 1

 0
 0
 0
 1

 0
 0
 0
 1

 0
 0
 0
 1

 0
 0
 0
 1

 0
 0
 0
 1

 0
 0
 0
 1

 0
 0
 0
 2

 0
 0
 0
 1

 0
 0
 0
 12

 0
 0
 0
 1

 0
 0
 0
 1

 0
 0
 0
 1

 0
 0
 0
 1

 0
 0
 0
 1

 0
 0
 0
 8

 0
 0
 0
 5

 0
 0
 0
 1

 0
 0
 0
 5

 0
 0
 0
 1

 0
 0
 0
 1

 0
 0
 0
 5

 0
 0
 0
 1

 0
 0
 0
 22

 0
 0
 0
 2

 0
 0
 0
 1

 0
 0
 0
 1

 0
 0
 0
 2

 10
 0
 5
 140

 1

 1

 2

 1

 3

 1

 1

 0
 0
 2

 0
 0
 1

 0
 0
 1

 0
 0
 1

 0
 0
 0
 3

 0
 0
 0
 8

 0
 0
 0
 2

 0
 0
 0
 3

 0
 0
 0
 3

 0
 0
 0
 2

 0
 0
 0
 1

 0
 0
 0
 4

 0
 0
 0
 1

 0
 0
 0
 14

 0
 0
 0
 5

 0
 0
 0
 3

 0
 0
 0
 8

 0
 0
 0
 4

 0
 0
 0
 1

 0
 0
 0
 4

 0
 0
 0
 72

 0
 0
 0
 1

 0
 0
 0
 1

 3
 0
 1
 32

 1

 2

 0
 0
 1
 8

 0
 0
 0
 1

 0
 0
 0
 1

 0
 0
 0
 1

 0
 0
 0
 7

 0
 0
 0
 3

 0
 0
 0
 9

 0
 0
 0
 1

 0
 0
 0
 1

 2
 0
 12
 291

 1

 1
 0
 11
 274

 0
 0
 1

 0
 0
 0
 1

 0
 0
 0
 2

 0
 0
 0
 1

 0
 0
 0
 1

 0
 0
 0
 1

 0
 0
 0
 2

 0
 0
 0
 1

 0
 0
 0
 1

 0
 0
 0
 1

 0
 0
 0
 4

 0
 0
 0
 1

 0
 0
 0
 1

 1
 0
 2
 1

 1

 0
 0
 1

 0
 0
 1

 0
 0
 0
 1

 108
 48
 95
 2331

 1

 1

 2

 1
 0
 0
 1

 1

 1

 1

 1

 1

 1

 1

 1

 1

 1

 1
 0
 0
 10

 1

 1

 3
 0
 2

 1

 1

 2

 2

 1
 0
 2

 2

 1

 1
 0
 1

 1

 1

 1
 0
 1

 1
 0
 1

 1

 1

 3

 1
 0
 1

 1
 0
 10
 248

 7
 0
 0
 3

 2
 0
 3

 4
 0
 2

 1

 1

 1

 1
 23
 10
 2

 1

 1

 4
 0
 2

 2

 1
 0
 3
 158

 1

 1

 1

 1

 2

 1

 2
 0
 1

 1

 3

 3
 0
 0
 398

 1

 2
 0
 0
 1

 1

 2

 1

 1

 1

 1

 1

 1

 1

 1

 1

 2

 1

 1

 1

 1

 1

 1

 0
 3

 0
 1

 0
 3

 0
 1
 1

 0
 9
 5

 0
 2

 0
 4

 0
 2

 0
 0
 1

 0
 0
 1

 0
 0
 1

 0
 0
 1

 0
 0
 1

 0
 0
 1

 0
 0
 1

 0
 0
 1
 35

 0
 0
 1
 3

 0
 0
 1

 0
 0
 2

 0
 0
 2
 49

 0
 0
 1

 0
 0
 1

 0
 0
 1

 0
 0
 1

 0
 0
 3

 0
 0
 1

 0
 0
 4
 222

 0
 0
 1

 0
 0
 1

 0
 0
 3
 305

 0
 0
 1
 4

 0
 0
 1
 35

 0
 0
 1

 0
 0
 4

 0
 0
 1
 116

 0
 0
 2

 0
 0
 1

 0
 0
 1

 0
 0
 1

 0
 0
 1

 0
 0
 1

 0
 0
 1

 0
 0
 1

 0
 0
 1

 0
 0
 1
 8

 0
 0
 0
 2

 0
 0
 0
 1

 0
 0
 0
 1

 0
 0
 0
 1

 0
 0
 0
 1

 0
 0
 0
 1

 0
 0
 0
 2

 0
 0
 0
 1

 0
 0
 0
 1

 0
 0
 0
 1

 0
 0
 0
 1

 0
 0
 0
 1

 0
 0
 0
 1

 0
 0
 0
 1

 0
 0
 0
 9

 0
 0
 0
 1

 0
 0
 0
 1

 0
 0
 0
 2

 0
 0
 0
 1

 0
 0
 0
 1

 0
 0
 0
 3

 0
 0
 0
 2

 0
 0
 0
 1

 0
 0
 0
 7

 0
 0
 0
 1

 0
 0
 0
 1

 0
 0
 0
 1

 0
 0
 0
 1

 0
 0
 0
 2

 0
 0
 0
 10

 0
 0
 0
 1

 0
 0
 0
 2

 0
 0
 0
 10

 0
 0
 0
 3

 0
 0
 0
 4

 0
 0
 0
 2

 0
 0
 0
 5

 0
 0
 0
 1

 0
 0
 0
 2

 0
 0
 0
 5

 0
 0
 0
 4

 0
 0
 0
 1

 0
 0
 0
 11

 0
 0
 0
 1

 0
 0
 0
 2

 0
 0
 0
 1

 0
 0
 0
 4

 0
 0
 0
 6

 0
 0
 0
 1

 0
 0
 0
 2

 0
 0
 0
 1

 0
 0
 0
 2

 0
 0
 0
 3

 0
 0
 0
 3

 0
 0
 0
 13

 0
 0
 0
 27

 0
 0
 0
 3

 0
 0
 0
 9

 0
 0
 0
 3

 0
 0
 0
 2

 0
 0
 0
 1

 0
 0
 0
 2

 0
 0
 0
 3

 0
 0
 0
 8

 0
 0
 0
 2

 0
 0
 0
 14

 0
 0
 0
 9

 0
 0
 0
 1

 0
 0
 0
 2

 0
 0
 0
 1

 0
 0
 0
 61

 0
 0
 0
 7

 0
 0
 0
 9

 0
 0
 0
 5

 0
 0
 0
 5

 0
 0
 0
 19

 0
 0
 0
 2

 0
 0
 0
 1

 0
 0
 0
 2

 0
 0
 0
 4

 0
 0
 0
 19

 0
 0
 0
 4

 0
 0
 0
 1

 0
 0
 0
 1

 0
 0
 0
 2

 0
 0
 0
 5

 0
 0
 0
 1

 0
 0
 0
 9

 0
 0
 0
 1

 0
 0
 0
 2

 0
 0
 0
 15

 0
 0
 0
 20

 0
 0
 0
 5

 0
 0
 0
 1

 0
 0
 0
 1

 0
 0
 0
 26

 0
 0
 0
 12

 0
 0
 0
 50

 0
 0
 0
 5

 0
 0
 0
 3

 0
 0
 0
 3

 0
 0
 0
 1

 0
 0
 0
 2

 0
 0
 0
 4

 0
 0
 0
 3

 0
 0
 0
 2

 0
 0
 0
 10

 0
 0
 0
 2

 0
 0
 0
 1

 0
 0
 0
 2

 0
 0
 0
 1

 0
 0
 0
 5

 0
 0
 0
 1

 0
 0
 0
 1

 0
 0
 0
 2

 0
 0
 0
 1

 0
 0
 0
 16

 0
 0
 0
 4

 0
 0
 0
 1

 0
 0
 0
 59

 0
 0
 0
 2

 0
 0
 0
 4

 0
 0
 0
 2

 0
 0
 0
 2

 0
 0
 0
 20

 0
 0
 0
 5

 0
 0
 0
 5

 0
 0
 0
 6

 0
 0
 0
 1

 0
 0
 0
 2

 0
 0
 0
 27

 0
 0
 0
 1

 1
 92
 11
 1

 1
 5
 7

 0
 1

 0
 2

 0
 51
 4
 1

 0
 15

 0
 11

 0
 4

 0
 1

 0
 2

 4
 0
 3
 451

 1

 1

 2
 0
 0
 427

 0
 0
 1

 0
 0
 1

 0
 0
 1

 0
 0
 0
 1

 0
 0
 0
 1

 0
 0
 0
 2

 0
 0
 0
 1

 0
 0
 0
 1

 0
 0
 0
 1

 0
 0
 0
 2

 0
 0
 0
 1

 0
 0
 0
 7

 0
 0
 0
 1

 0
 0
 0
 4

 0
 0
 0
 1

 0
 0
 0
 1

 0
 98
 6

 0
 1

 0
 3

 0
 10

 0
 37
 5

 0
 44

 0
 3

 0
 0
 1

 0
 4
 1

 0
 3

 0
 1
 1

 0
 4

 0
 2

 0
 1

 0
 1

 0
 139
 47

 0
 2

 0
 20
 18

 0
 112
 9

 0
 1

 0
 1

 0
 3

 0
 0
 19

 0
 0
 1

 0
 13
 2

 0
 3

 0
 7
 2

 0
 2

 0
 1

 0
 222
 37

 0
 1

 0
 1

 0
 72
 24

 0
 146
 10

 0
 1

 0
 1

 0
 0
 3

 0
 0
 1
 2

 0
 0
 1

 0
 0
 0
 2

 0
 0
 1

 0
 0
 1

 0
 0
 3
 125

 0
 0
 3
 125

 0
 0
 0
 1

 0
 0
 0
 1

 0
 0
 0
 1

 0
 0
 0
 1

 0
 0
 0
 3

 0
 0
 0
 3

 0
 0
 0
 1

 0
 0
 0
 1

 1
 0
 12
 14

 1
 0
 1

 1

 0
 0
 1

 0
 0
 5

 0
 0
 1

 0
 0
 3

 0
 0
 1

 0
 0
 1

 0
 0
 1

 0
 0
 5
 14

 0
 0
 5

 0
 0
 0
 14

 3
 1
 244

 1
 0
 4

 1

 0
 0
 4

 2
 1
 240

 1
 1
 33

 1
 0
 16

 0
 0
 3

 0
 0
 1

 0
 0
 35

 0
 0
 5

 0
 0
 30

 0
 0
 95

 0
 0
 1

 0
 0
 6

 0
 0
 6

 0
 0
 7

 0
 0
 2

 26
 837
 112
 14

 16
 0
 27
 3

 5
 0
 26

 1

 10

 0
 0
 1

 0
 0
 0
 3

 4
 0
 19

 3
 0
 17

 1

 0
 0
 1

 0
 0
 1

 2
 716
 57
 3

 1

 1
 707
 36

 0
 1

 0
 3

 0
 1

 0
 1

 0
 1

 0
 1

 0
 1

 0
 0
 21
 3

 3
 0
 0
 1

 1

 1

 1

 0
 0
 0
 1

 1
 0
 0
 6

 1

 0
 0
 0
 3

 0
 0
 0
 1

 0
 0
 0
 1

 0
 0
 0
 1

 0
 121
 4

 0
 120
 4

 0
 1

 0
 0
 1
 1

 0
 0
 1

 0
 0
 0
 1

 0
 0
 4

 0
 0
 3

 0
 0
 1

 237
 0
 97
 2835

 3
 0
 0
 39

 1
 0
 0
 2

 1

 1

 0
 0
 0
 1

 0
 0
 0
 28

 0
 0
 0
 2

 0
 0
 0
 4

 0
 0
 0
 1

 0
 0
 0
 1

 3
 0
 1
 14

 1

 1

 1

 0
 0
 1

 0
 0
 0
 14

 6
 0
 4
 330

 1

 3

 1

 1

 0
 0
 1
 85

 0
 0
 1
 24

 0
 0
 1
 99

 0
 0
 1

 0
 0
 0
 2

 0
 0
 0
 1

 0
 0
 0
 1

 0
 0
 0
 1

 0
 0
 0
 1

 0
 0
 0
 30

 0
 0
 0
 5

 0
 0
 0
 1

 0
 0
 0
 4

 0
 0
 0
 1

 0
 0
 0
 2

 0
 0
 0
 1

 0
 0
 0
 2

 0
 0
 0
 23

 0
 0
 0
 27

 0
 0
 0
 8

 0
 0
 0
 2

 0
 0
 0
 1

 0
 0
 0
 1

 0
 0
 0
 6

 0
 0
 0
 2

 95
 0
 11
 617

 1

 1

 1

 1

 61
 0
 1

 28
 0
 1

 1

 1

 0
 0
 1

 0
 0
 4
 22

 0
 0
 2

 0
 0
 1

 0
 0
 1

 0
 0
 0
 1

 0
 0
 0
 1

 0
 0
 0
 1

 0
 0
 0
 9

 0
 0
 0
 1

 0
 0
 0
 1

 0
 0
 0
 2

 0
 0
 0
 55

 0
 0
 0
 2

 0
 0
 0
 1

 0
 0
 0
 1

 0
 0
 0
 1

 0
 0
 0
 1

 0
 0
 0
 1

 0
 0
 0
 3

 0
 0
 0
 5

 0
 0
 0
 1

 0
 0
 0
 9

 0
 0
 0
 13

 0
 0
 0
 6

 0
 0
 0
 5

 0
 0
 0
 24

 0
 0
 0
 1

 0
 0
 0
 99

 0
 0
 0
 2

 0
 0
 0
 1

 0
 0
 0
 1

 0
 0
 0
 19

 0
 0
 0
 75

 0
 0
 0
 15

 0
 0
 0
 34

 0
 0
 0
 2

 0
 0
 0
 9

 0
 0
 0
 6

 0
 0
 0
 1

 0
 0
 0
 1

 0
 0
 0
 47

 0
 0
 0
 2

 0
 0
 0
 3

 0
 0
 0
 1

 0
 0
 0
 7

 0
 0
 0
 1

 0
 0
 0
 72

 0
 0
 0
 1

 0
 0
 0
 1

 0
 0
 0
 27

 0
 0
 0
 1

 0
 0
 0
 1

 0
 0
 0
 1

 0
 0
 0
 12

 0
 0
 0
 8

 2
 0
 1

 2
 0
 1

 1
 0
 1
 2

 1
 0
 1

 0
 0
 0
 2

 4
 0
 3
 257

 2

 1
 0
 0
 50

 1

 0
 0
 1

 0
 0
 1
 20

 0
 0
 1

 0
 0
 0
 1

 0
 0
 0
 1

 0
 0
 0
 5

 0
 0
 0
 1

 0
 0
 0
 2

 0
 0
 0
 2

 0
 0
 0
 9

 0
 0
 0
 4

 0
 0
 0
 20

 0
 0
 0
 1

 0
 0
 0
 1

 0
 0
 0
 81

 0
 0
 0
 9

 0
 0
 0
 1

 0
 0
 0
 5

 0
 0
 0
 2

 0
 0
 0
 8

 0
 0
 0
 21

 0
 0
 0
 2

 0
 0
 0
 1

 0
 0
 0
 4

 0
 0
 0
 1

 0
 0
 0
 1

 0
 0
 0
 3

 0
 0
 0
 1

 118
 0
 55
 1210

 1

 1

 1

 1

 1

 1

 1

 1

 1

 1

 1

 1
 0
 0
 41

 1

 1

 2

 1

 2

 1
 0
 2

 1

 1

 2

 1

 1

 1
 0
 0
 2

 31
 0
 8

 1

 1

 1
 0
 0
 14

 4

 3
 0
 5

 4
 0
 4

 1

 2

 4
 0
 3

 1

 1

 2

 1

 1

 1

 2

 2

 1

 2
 0
 1

 2

 1

 1

 1

 1
 0
 0
 138

 1

 1

 1

 1

 1

 1

 1

 1

 1

 1

 1
 0
 1

 1

 1

 1

 1

 1

 1

 1

 1

 0
 0
 1

 0
 0
 1

 0
 0
 1

 0
 0
 1

 0
 0
 1
 4

 0
 0
 1

 0
 0
 1
 18

 0
 0
 1

 0
 0
 1

 0
 0
 1

 0
 0
 3

 0
 0
 1

 0
 0
 2

 0
 0
 4
 54

 0
 0
 1

 0
 0
 1
 3

 0
 0
 2

 0
 0
 1

 0
 0
 2

 0
 0
 1
 24

 0
 0
 1

 0
 0
 1

 0
 0
 1

 0
 0
 0
 2

 0
 0
 0
 1

 0
 0
 0
 1

 0
 0
 0
 3

 0
 0
 0
 1

 0
 0
 0
 2

 0
 0
 0
 1

 0
 0
 0
 1

 0
 0
 0
 3

 0
 0
 0
 1

 0
 0
 0
 1

 0
 0
 0
 1

 0
 0
 0
 3

 0
 0
 0
 1

 0
 0
 0
 1

 0
 0
 0
 1

 0
 0
 0
 3

 0
 0
 0
 1

 0
 0
 0
 1

 0
 0
 0
 1

 0
 0
 0
 1

 0
 0
 0
 5

 0
 0
 0
 34

 0
 0
 0
 1

 0
 0
 0
 3

 0
 0
 0
 1

 0
 0
 0
 1

 0
 0
 0
 3

 0
 0
 0
 1

 0
 0
 0
 1

 0
 0
 0
 1

 0
 0
 0
 2

 0
 0
 0
 1

 0
 0
 0
 1

 0
 0
 0
 2

 0
 0
 0
 2

 0
 0
 0
 1

 0
 0
 0
 1

 0
 0
 0
 1

 0
 0
 0
 1

 0
 0
 0
 1

 0
 0
 0
 1

 0
 0
 0
 1

 0
 0
 0
 1

 0
 0
 0
 1

 0
 0
 0
 1

 0
 0
 0
 3

 0
 0
 0
 1

 0
 0
 0
 1

 0
 0
 0
 1

 0
 0
 0
 4

 0
 0
 0
 1

 0
 0
 0
 10

 0
 0
 0
 1

 0
 0
 0
 1

 0
 0
 0
 2

 0
 0
 0
 7

 0
 0
 0
 3

 0
 0
 0
 2

 0
 0
 0
 2

 0
 0
 0
 2

 0
 0
 0
 1

 0
 0
 0
 5

 0
 0
 0
 2

 0
 0
 0
 1

 0
 0
 0
 1

 0
 0
 0
 2

 0
 0
 0
 4

 0
 0
 0
 3

 0
 0
 0
 1

 0
 0
 0
 3

 0
 0
 0
 6

 0
 0
 0
 1

 0
 0
 0
 1

 0
 0
 0
 1

 0
 0
 0
 2

 0
 0
 0
 1

 0
 0
 0
 1

 0
 0
 0
 29

 0
 0
 0
 1

 0
 0
 0
 10

 0
 0
 0
 2

 0
 0
 0
 5

 0
 0
 0
 1

 0
 0
 0
 24

 0
 0
 0
 2

 0
 0
 0
 2

 0
 0
 0
 3

 0
 0
 0
 7

 0
 0
 0
 24

 0
 0
 0
 2

 0
 0
 0
 7

 0
 0
 0
 6

 0
 0
 0
 7

 0
 0
 0
 13

 0
 0
 0
 1

 0
 0
 0
 26

 0
 0
 0
 4

 0
 0
 0
 1

 0
 0
 0
 86

 0
 0
 0
 6

 0
 0
 0
 4

 0
 0
 0
 6

 0
 0
 0
 5

 0
 0
 0
 4

 0
 0
 0
 6

 0
 0
 0
 1

 0
 0
 0
 1

 0
 0
 0
 1

 0
 0
 0
 1

 0
 0
 0
 2

 0
 0
 0
 1

 0
 0
 0
 21

 0
 0
 0
 16

 0
 0
 0
 180

 0
 0
 0
 2

 0
 0
 0
 1

 0
 0
 0
 5

 0
 0
 0
 9

 0
 0
 0
 17

 0
 0
 0
 3

 0
 0
 0
 1

 0
 0
 0
 8

 0
 0
 0
 9

 0
 0
 0
 6

 0
 0
 0
 1

 0
 0
 0
 27

 0
 0
 0
 2

 0
 0
 0
 1

 0
 0
 0
 2

 0
 0
 0
 1

 0
 0
 0
 2

 0
 0
 0
 1

 0
 0
 0
 1

 0
 0
 0
 6

 0
 0
 0
 1

 0
 0
 0
 1

 0
 0
 0
 6

 0
 0
 0
 3

 0
 0
 0
 2

 0
 0
 0
 3

 0
 0
 0
 4

 0
 0
 0
 2

 0
 0
 0
 15

 0
 0
 0
 2

 0
 0
 0
 4

 0
 0
 0
 1

 0
 0
 0
 1

 0
 0
 0
 3

 0
 0
 0
 6

 0
 0
 0
 1

 0
 0
 0
 2

 0
 0
 0
 1

 0
 0
 0
 1

 0
 0
 0
 3

 0
 0
 0
 1

 0
 0
 0
 1

 0
 0
 0
 1

 0
 0
 0
 1

 0
 0
 0
 1

 0
 0
 0
 14

 0
 0
 0
 9

 0
 0
 0
 1

 0
 0
 0
 1

 0
 0
 0
 1

 0
 0
 0
 1

 0
 0
 0
 1

 0
 0
 0
 1

 0
 0
 0
 3

 0
 0
 0
 7

 0
 0
 0
 4

 0
 0
 0
 2

 1
 0
 10
 322

 1

 0
 0
 9
 82

 0
 0
 1
 148

 0
 0
 0
 1

 0
 0
 0
 1

 0
 0
 0
 7

 0
 0
 0
 1

 0
 0
 0
 15

 0
 0
 0
 2

 0
 0
 0
 1

 0
 0
 0
 30

 0
 0
 0
 25

 0
 0
 0
 8

 0
 0
 0
 1

 1
 0
 3

 1

 0
 0
 3

 3
 0
 0
 9

 3

 0
 0
 0
 6

 0
 0
 0
 2

 0
 0
 0
 1

 0
 0
 1
 6

 0
 0
 1
 2

 0
 0
 0
 2

 0
 0
 0
 2

 0
 0
 6

 0
 0
 3

 0
 0
 1

 0
 0
 1

 0
 0
 1

 0
 0
 1

 0
 0
 1

 0
 0
 0
 7

 0
 0
 0
 7

 0
 0
 0
 2

 0
 0
 0
 1

 0
 0
 0
 1

 0
 0
 0
 9

 0
 0
 0
 1

 0
 0
 0
 6

 0
 0
 0
 1

 0
 0
 0
 1

 0
 0
 0
 11

 0
 0
 0
 3

 0
 0
 0
 1

 0
 0
 0
 1

 0
 0
 0
 6

 0
 87
 3
 4

 0
 75
 3

 0
 1

 0
 69
 3

 0
 1

 0
 3

 0
 1

 0
 12

 0
 12

 0
 0
 0
 4

 0
 0
 0
 1

 0
 0
 0
 2

 0
 0
 0
 1

 0
 0
 6

 0
 0
 6

 0
 0
 6

 0
 0
 1

 0
 0
 1

 0
 0
 1

 0
 0
 0
 1

 0
 0
 0
 1

 0
 0
 0
 1

 0
 0
 0
 2

 0
 0
 0
 2

 0
 0
 0
 1

 0
 0
 0
 1

 40
 27
 35
 259

 40
 27
 35
 259

 40
 27
 35
 259

 3
 0
 2
 110

 3
 0
 2
 110

 4
 0
 3
 33

 1

 1
 0
 0
 18

 2
 0
 1

 0
 0
 2
 15

 8
 0
 10

 3

 3
 0
 9

 1

 1

 0
 0
 1

 8
 0
 9
 5

 1

 1

 1

 1
 0
 1

 1

 2

 1

 0
 0
 1

 0
 0
 1

 0
 0
 1
 3

 0
 0
 1

 0
 0
 1

 0
 0
 1

 0
 0
 1

 0
 0
 1

 0
 0
 0
 1

 0
 0
 0
 1

 1
 0
 0
 7

 1
 0
 0
 3

 0
 0
 0
 1

 0
 0
 0
 3

 7

 7

 9
 0
 9
 62

 1

 5
 0
 8
 62

 1

 1

 1

 0
 0
 1

 0
 3

 0
 3

 0
 18

 0
 1

 0
 17

 0
 6

 0
 1

 0
 2

 0
 1

 0
 1

 0
 1

 0
 0
 2
 35

 0
 0
 2
 5

 0
 0
 0
 17

 0
 0
 0
 13

 0
 0
 0
 7

 0
 0
 0
 5

 0
 0
 0
 2

 29
 48
 29
 383

 29
 48
 29
 383

 29
 48
 29
 383

 29
 48
 29
 383

 1

 1

 1

 1

 1

 1

 1

 1

 1

 1

 1

 1

 2

 1

 1
 0
 1

 3

 1

 3
 0
 2

 1
 0
 1

 1
 0
 1

 1

 1

 1

 1

 0
 1

 0
 46
 1

 0
 1

 0
 0
 1

 0
 0
 5

 0
 0
 2

 0
 0
 1

 0
 0
 1

 0
 0
 1

 0
 0
 1

 0
 0
 2

 0
 0
 1

 0
 0
 1

 0
 0
 1

 0
 0
 1

 0
 0
 1

 0
 0
 1

 0
 0
 1

 0
 0
 1

 0
 0
 1

 0
 0
 0
 5

 0
 0
 0
 15

 0
 0
 0
 1

 0
 0
 0
 4

 0
 0
 0
 1

 0
 0
 0
 3

 0
 0
 0
 1

 0
 0
 0
 5

 0
 0
 0
 3

 0
 0
 0
 1

 0
 0
 0
 1

 0
 0
 0
 1

 0
 0
 0
 1

 0
 0
 0
 2

 0
 0
 0
 1

 0
 0
 0
 1

 0
 0
 0
 1

 0
 0
 0
 1

 0
 0
 0
 1

 0
 0
 0
 1

 0
 0
 0
 1

 0
 0
 0
 9

 0
 0
 0
 12

 0
 0
 0
 6

 0
 0
 0
 17

 0
 0
 0
 58

 0
 0
 0
 8

 0
 0
 0
 111

 0
 0
 0
 3

 0
 0
 0
 5

 0
 0
 0
 2

 0
 0
 0
 1

 0
 0
 0
 9

 0
 0
 0
 16

 0
 0
 0
 1

 0
 0
 0
 8

 0
 0
 0
 4

 0
 0
 0
 11

 0
 0
 0
 9

 0
 0
 0
 2

 0
 0
 0
 6

 0
 0
 0
 2

 0
 0
 0
 2

 0
 0
 0
 1

 0
 0
 0
 3

 0
 0
 0
 2

 0
 0
 0
 1

 0
 0
 0
 4

 0
 0
 0
 7

 0
 0
 0
 1

 0
 0
 0
 1

 0
 0
 0
 1

 0
 0
 0
 8

 0
 0
 0
 1

 16
 721
 164
 1063

 16
 721
 164
 1063

 11
 0
 4
 320

 1
 0
 4

 1
 0
 3

 0
 0
 1

 10
 0
 0
 6

 1

 8

 1

 0
 0
 0
 1

 0
 0
 0
 1

 0
 0
 0
 1

 0
 0
 0
 3

 0
 0
 0
 314

 0
 0
 0
 314

 5
 721
 160
 743

 1

 1

 1
 4
 12
 54

 1
 0
 0
 33

 0
 2

 0
 2

 0
 0
 12
 1

 0
 0
 0
 1

 0
 0
 0
 2

 0
 0
 0
 16

 0
 0
 0
 1

 1
 0
 1

 1

 0
 0
 1

 1
 132
 10
 16

 1

 0
 2

 0
 2

 0
 1

 0
 1

 0
 2

 0
 2

 0
 1

 0
 6

 0
 1

 0
 1

 0
 2

 0
 1

 0
 1

 0
 1

 0
 2

 0
 1

 0
 1

 0
 1

 0
 1

 0
 1

 0
 1

 0
 1

 0
 1

 0
 1

 0
 1

 0
 3

 0
 1

 0
 1

 0
 1

 0
 1

 0
 1

 0
 1

 0
 8

 0
 3

 0
 1

 0
 11

 0
 1

 0
 8

 0
 9
 1

 0
 1

 0
 2

 0
 1

 0
 1

 0
 1

 0
 2

 0
 1

 0
 3

 0
 1

 0
 6

 0
 1

 0
 5

 0
 1

 0
 1

 0
 1

 0
 1

 0
 1

 0
 1

 0
 1

 0
 1

 0
 1

 0
 1

 0
 3

 0
 2

 0
 1

 0
 1

 0
 1

 0
 1

 0
 1

 0
 1

 0
 0
 7

 0
 0
 2

 0
 0
 0
 16

 1
 156
 22

 1

 0
 1

 0
 1

 0
 1

 0
 2

 0
 2

 0
 1

 0
 1

 0
 2
 1

 0
 9

 0
 40

 0
 13
 4

 0
 10
 6

 0
 2

 0
 23

 0
 8
 1

 0
 35

 0
 1

 0
 1

 0
 2

 0
 1

 0
 0
 1

 0
 0
 1

 0
 0
 2

 0
 0
 5

 0
 0
 1

 0
 15
 2

 0
 5

 0
 10
 2

 0
 63
 32
 626

 0
 1

 0
 51
 2

 0
 1

 0
 8

 0
 2

 0
 0
 1

 0
 0
 21

 0
 0
 1
 225

 0
 0
 7

 0
 0
 0
 1

 0
 0
 0
 23

 0
 0
 0
 1

 0
 0
 0
 273

 0
 0
 0
 1

 0
 0
 0
 102

 0
 2

 0
 2

 0
 349
 69
 5

 0
 3

 0
 1

 0
 1

 0
 1

 0
 1

 0
 1

 0
 13

 0
 46
 19
 3

 0
 183
 42
 2

 0
 5

 0
 36
 3

 0
 46
 4

 0
 1

 0
 6

 0
 1

 0
 1

 0
 1

 0
 1

 0
 1

 0
 0
 1

 0
 0
 9

 0
 0
 7

 0
 0
 2

 0
 0
 3

 0
 0
 2

 0
 0
 1

 0
 0
 0
 41

 0
 0
 0
 3

 0
 0
 0
 36

 0
 0
 0
 2

 0
 0
 0
 1

 0
 0
 0
 1

 88
 5555
 538
 1

 88
 5555
 538
 1

 88
 5555
 538
 1

 78
 838
 63

 52
 0
 2

 1

 3

 3

 45

 0
 0
 2

 26
 838
 61

 1

 2
 386
 11

 1
 19
 31

 16

 1

 1

 3

 1

 0
 1

 0
 1

 0
 1

 0
 2

 0
 1

 0
 1

 0
 1

 0
 2

 0
 1

 0
 1

 0
 3

 0
 1

 0
 3

 0
 1

 0
 1

 0
 9

 0
 3

 0
 4

 0
 1

 0
 1

 0
 23

 0
 3

 0
 3

 0
 3

 0
 4

 0
 1

 0
 2

 0
 107

 0
 11

 0
 2
 7

 0
 5

 0
 92

 0
 69
 8

 0
 2

 0
 4

 0
 3

 0
 1

 0
 13

 0
 2

 0
 1

 0
 2

 0
 5

 0
 9

 0
 6

 0
 1

 0
 2

 0
 1

 0
 1

 0
 1

 0
 2

 0
 2

 0
 1

 0
 2

 0
 1

 0
 1

 0
 1

 0
 1

 0
 1

 0
 1

 0
 1

 0
 1

 0
 0
 2

 0
 0
 1

 0
 0
 1

 10
 4717
 475
 1

 10
 3844
 396

 1

 3
 3
 3

 1
 10

 2
 380
 95

 1
 783
 46

 1
 935
 137

 1
 2
 3

 0
 1

 0
 1

 0
 2

 0
 1

 0
 1

 0
 1

 0
 3

 0
 1

 0
 1

 0
 1

 0
 1

 0
 2

 0
 1

 0
 1

 0
 1

 0
 1

 0
 1

 0
 1

 0
 1

 0
 2

 0
 2
 1

 0
 1

 0
 1

 0
 1

 0
 1

 0
 1

 0
 7

 0
 1

 0
 2

 0
 2

 0
 1

 0
 1

 0
 21
 1

 0
 2

 0
 7

 0
 19
 28

 0
 5
 1

 0
 14
 3

 0
 1

 0
 5

 0
 9

 0
 95
 2

 0
 43
 6

 0
 10

 0
 1
 3

 0
 8
 1

 0
 10
 1

 0
 26

 0
 208
 2

 0
 126
 2

 0
 110
 2

 0
 297
 3

 0
 51
 2

 0
 36
 3

 0
 10
 2

 0
 116
 6

 0
 46

 0
 123
 26

 0
 106

 0
 8

 0
 11

 0
 8
 2

 0
 87

 0
 1

 0
 3

 0
 3

 0
 30
 1

 0
 4

 0
 1

 0
 1

 0
 2

 0
 1

 0
 1

 0
 1

 0
 1

 0
 1

 0
 1

 0
 1

 0
 1

 0
 1

 0
 1

 0
 1

 0
 1

 0
 4

 0
 1

 0
 1

 0
 1

 0
 1

 0
 1

 0
 0
 1

 0
 0
 1

 0
 0
 3

 0
 0
 2

 0
 0
 1

 0
 0
 1

 0
 0
 1

 0
 0
 1

 0
 0
 1

 0
 0
 1

 0
 0
 1

 0
 804
 70
 1

 0
 2

 0
 1

 0
 1

 0
 238
 8

 0
 561
 53

 0
 1

 0
 0
 9
 1

 0
 69
 9

 0
 69
 2

 0
 0
 7

 78
 0
 15

 78
 0
 15

 78
 0
 15

 78
 0
 15

 78
 0
 15

 1

 1

 1

 1

 1

 1

 1

 1
 0
 1

 2

 1

 1

 1

 2

 1

 1
 0
 1

 1

 1

 1

 3

 1
 0
 1

 1

 2

 1

 1

 1

 1

 2

 1

 1

 1

 1

 1

 1

 2

 1

 1

 1

 1

 1

 1

 1

 2

 2

 4

 1

 1

 1

 1

 1

 2

 2

 2

 1

 1

 1

 1

 1

 1

 1

 1

 1

 1

 1

 0
 0
 1

 0
 0
 1

 0
 0
 1

 0
 0
 1

 0
 0
 1

 0
 0
 1

 0
 0
 1

 0
 0
 1

 0
 0
 1

 0
 0
 1

 0
 0
 1

 0
 0
 1

 2
 0
 1

 2
 0
 1

 2
 0
 1

 2
 0
 1

 2
 0
 1

 1

 1

 0
 0
 1

 8
 0
 8

 8
 0
 8

 8
 0
 8

 8
 0
 8

 8
 0
 8

 1

 1

 4

 2

 0
 0
 2

 0
 0
 2

 0
 0
 1

 0
 0
 1

 0
 0
 1

 0
 0
 1

 6
 0
 1

 6
 0
 1

 6
 0
 1

 6
 0
 1

 6
 0
 1

 1

 1

 1

 1

 1

 1

 0
 0
 1

 207
 1
 115

 10

 10

 10

 10

 1

 1

 1

 1

 1

 1

 2

 1

 1

 197
 1
 115

 197
 1
 115

 197
 1
 115

 8
 0
 3

 1

 1

 2

 1

 1

 1

 1

 0
 0
 1

 0
 0
 1

 0
 0
 1

 14
 0
 3

 1

 1

 1

 1

 1

 1

 1

 1

 3

 1

 1

 1

 0
 0
 2

 0
 0
 1

 5

 1

 1

 1

 1

 1

 136
 1
 99

 1

 1

 1

 1

 1

 1

 1

 1

 1

 1

 1

 1

 1

 1

 1
 0
 1

 1

 1

 1

 1

 1

 1

 1
 0
 1

 1
 0
 1

 1

 1

 1

 1

 1

 1

 3

 1

 1

 1

 2

 1

 1

 1

 1

 1

 1

 3

 1

 1

 1

 1

 1

 1

 1

 1

 1

 1

 1

 1

 2

 2

 1

 1

 2

 4

 1

 1

 2

 2

 1

 1

 1

 1

 1

 1

 2

 1

 1

 2

 1

 1

 1

 1

 1

 1
 0
 1

 1

 2

 2

 1

 1

 1

 1

 1

 1

 1

 1

 1

 1

 1

 1

 1

 1

 1

 1

 1

 1

 1

 1

 2

 1

 1

 1

 1

 1

 1

 1

 1

 1

 1

 1

 1

 1

 1

 1

 0
 1

 0
 0
 1

 0
 0
 1

 0
 0
 1

 0
 0
 1

 0
 0
 1

 0
 0
 1

 0
 0
 1

 0
 0
 1

 0
 0
 1

 0
 0
 4

 0
 0
 1

 0
 0
 1

 0
 0
 1

 0
 0
 1

 0
 0
 1

 0
 0
 2

 0
 0
 1

 0
 0
 2

 0
 0
 1

 0
 0
 48

 0
 0
 1

 0
 0
 1

 0
 0
 1

 0
 0
 1

 0
 0
 1

 0
 0
 2

 0
 0
 2

 0
 0
 1

 0
 0
 1

 0
 0
 1

 0
 0
 1

 0
 0
 1

 0
 0
 1

 0
 0
 1

 0
 0
 1

 0
 0
 1

 0
 0
 1

 0
 0
 1

 0
 0
 1

 0
 0
 1

 0
 0
 1

 3
 0
 1

 1

 1

 1

 0
 0
 1

 1
 0
 1

 1

 0
 0
 1

 17
 0
 5

 1

 1

 1

 1

 4

 1

 1

 2

 1

 1

 1
 0
 1

 1

 1

 0
 0
 1

 0
 0
 1

 0
 0
 1

 0
 0
 1

 1
 0
 1

 1

 0
 0
 1

 12

 1

 1

 1

 3

 3

 1

 1

 1

 0
 0
 1

 0
 0
 1

 0
 0
 1

 0
 0
 1

 5260
 2444
 4458
 474

 2679
 4
 1262
 92

 8
 0
 14

 4
 0
 7

 4
 0
 7

 1

 2
 0
 1

 1

 0
 0
 1

 0
 0
 4

 0
 0
 1

 4
 0
 7

 4
 0
 7

 2

 1

 1

 0
 0
 1

 0
 0
 1

 0
 0
 1

 0
 0
 4

 131
 0
 47
 92

 131
 0
 47
 92

 131
 0
 47
 92

 1

 1

 1

 1

 1

 1

 1

 1

 1

 2

 1

 1

 1

 1

 1

 1

 1

 1

 1

 1

 1

 1

 1

 2

 1
 0
 1

 1

 3

 1

 1

 1

 1

 1

 1

 1

 1

 1

 7
 0
 1

 3

 1

 37
 0
 14

 1

 2
 0
 2

 3
 0
 1

 1

 2
 0
 1

 5

 1

 3

 1

 1

 1

 3

 1

 1

 1

 2

 2

 1

 1

 1

 1

 1

 1

 1

 1

 1

 1

 2

 0
 0
 1

 0
 0
 1

 0
 0
 2

 0
 0
 1

 0
 0
 1

 0
 0
 3

 0
 0
 1

 0
 0
 1

 0
 0
 1

 0
 0
 1

 0
 0
 1

 0
 0
 1

 0
 0
 1

 0
 0
 1

 0
 0
 3

 0
 0
 1

 0
 0
 1

 0
 0
 1

 0
 0
 1

 0
 0
 1

 0
 0
 2

 0
 0
 0
 1

 0
 0
 0
 69

 0
 0
 0
 13

 0
 0
 0
 2

 0
 0
 0
 3

 0
 0
 0
 1

 0
 0
 0
 3

 195
 1
 51

 195
 1
 51

 11
 0
 3

 1

 8
 0
 3

 1

 1

 35
 0
 15

 1

 2
 0
 1

 12
 0
 2

 15
 0
 2

 1

 3

 1

 0
 0
 1

 0
 0
 6

 0
 0
 2

 0
 0
 1

 64
 0
 16

 1

 1

 7

 2
 0
 1

 1

 2
 0
 1

 41
 0
 1

 1
 0
 11

 2

 2

 1

 1

 1

 1

 0
 0
 1

 0
 0
 1

 34
 0
 9

 1

 1

 2

 2
 0
 1

 1

 14
 0
 1

 2

 5
 0
 4

 1

 1

 1

 1

 1

 1

 0
 0
 1

 0
 0
 1

 0
 0
 1

 51
 1
 8

 1

 1

 1

 1

 1

 3

 1

 6
 0
 2

 2

 3
 0
 1

 5
 0
 1

 11

 1

 1

 1

 1

 2

 1

 1

 1

 1

 1

 1

 2

 1

 0
 1
 1

 0
 0
 1

 0
 0
 1

 0
 0
 1

 804
 2
 302

 15
 0
 24

 1

 1

 14
 0
 24

 9

 4
 0
 4

 1

 0
 0
 1

 0
 0
 4

 0
 0
 2

 0
 0
 13

 15
 0
 1

 8

 1

 1

 2

 3

 1

 7

 2

 1

 1

 3

 0
 0
 1

 0
 0
 1

 92
 0
 21

 1

 1

 11
 0
 10

 1

 1

 8
 0
 1

 1

 0
 0
 1

 0
 0
 4

 0
 0
 4

 66
 0
 7

 1

 2

 1

 1

 1

 1

 1

 1

 5

 19
 0
 1

 1

 6

 3

 1

 2

 4
 0
 1

 4

 1

 1

 1

 1

 3

 1

 1

 1

 1

 1

 0
 0
 1

 0
 0
 1

 0
 0
 1

 0
 0
 2

 14
 0
 1

 1

 8

 4

 1

 0
 0
 1

 0
 0
 3

 0
 0
 1

 0
 0
 1

 0
 0
 1

 133
 0
 40

 108
 0
 16

 1

 1

 1

 1

 1

 1

 4

 1

 1

 1

 3

 2
 0
 1

 1
 0
 3

 2
 0
 1

 10

 17

 23
 0
 1

 10

 6

 6
 0
 1

 1

 1

 4
 0
 2

 1

 2

 3

 1

 1

 1

 0
 0
 1

 0
 0
 1

 0
 0
 3

 0
 0
 1

 0
 0
 1

 10
 0
 7

 1

 1

 2

 1

 1
 0
 2

 1

 1

 1

 1

 0
 0
 1

 0
 0
 2

 0
 0
 1

 0
 0
 1

 8
 0
 9

 1

 2

 3
 0
 4

 2
 0
 1

 0
 0
 3

 0
 0
 1

 4

 1

 2

 1

 3
 0
 2

 1

 2
 0
 1

 0
 0
 1

 0
 0
 6

 0
 0
 6

 111
 0
 41

 8
 0
 3

 1

 1

 4
 0
 1

 1

 1

 0
 0
 2

 91
 0
 37

 1

 1

 2

 4
 0
 3

 21
 0
 8

 49
 0
 4

 1

 3
 0
 3

 1
 0
 1

 3
 0
 1

 2

 1

 1

 1

 0
 0
 2

 0
 0
 2

 0
 0
 7

 0
 0
 3

 0
 0
 1

 0
 0
 2

 3
 0
 1

 1

 1

 1

 0
 0
 1

 9

 1

 8

 1
 0
 1

 1

 1

 0
 0
 1

 0
 0
 1

 28
 0
 7

 21
 0
 2

 21
 0
 2

 5
 0
 2

 5

 0
 0
 1

 0
 0
 1

 2
 0
 3

 1

 1

 0
 0
 1

 0
 0
 1

 0
 0
 1

 105
 0
 32

 3
 0
 1

 1

 1
 0
 1

 1

 27
 0
 8

 27
 0
 7

 0
 0
 1

 10
 0
 9

 1

 1

 4
 0
 5

 1

 3

 0
 0
 1

 0
 0
 1

 0
 0
 2

 65
 0
 14

 1
 0
 5

 1

 4

 12

 4

 33
 0
 2

 5

 1

 4
 0
 3

 0
 0
 4

 16
 0
 2

 13
 0
 1

 1

 10
 0
 1

 2

 3

 2

 1

 0
 0
 1

 0
 0
 1

 257
 2
 117

 257
 2
 117

 1

 1

 1

 1

 1

 1

 1

 1

 1

 1

 1

 1

 1

 2

 1

 1

 1

 2

 2

 1

 1

 1

 1

 1

 1

 1

 1

 1

 6

 3
 0
 2

 2

 1
 0
 2

 2

 1

 1

 2
 0
 1

 1

 2

 1

 1

 2

 4

 1
 0
 3

 2

 1

 1

 1

 1

 1

 22

 3
 0
 25

 4

 4
 0
 3

 9
 0
 8

 11
 0
 11

 8

 14

 4

 1

 10

 1

 11

 1
 0
 1

 2

 1

 3

 2

 2

 2

 8

 2
 0
 2

 2

 2

 2

 2
 0
 2

 2

 1

 1

 1

 1

 1

 2

 2

 3

 1

 1

 8
 0
 1

 3

 2
 0
 5

 1

 2
 0
 1

 2

 1
 0
 1

 1

 1

 1

 1

 1

 1

 1

 1

 1

 1

 1

 2

 1

 1

 1

 1

 1

 1

 1

 1

 0
 2

 0
 0
 1

 0
 0
 1

 0
 0
 1

 0
 0
 2

 0
 0
 4

 0
 0
 2

 0
 0
 1

 0
 0
 1

 0
 0
 1

 0
 0
 1

 0
 0
 1

 0
 0
 1

 0
 0
 1

 0
 0
 5

 0
 0
 1

 0
 0
 1

 0
 0
 1

 0
 0
 2

 0
 0
 1

 0
 0
 1

 0
 0
 1

 0
 0
 1

 0
 0
 3

 0
 0
 2

 0
 0
 1

 0
 0
 1

 0
 0
 1

 0
 0
 1

 0
 0
 1

 0
 0
 2

 0
 0
 2

 0
 0
 1

 0
 0
 2

 17
 0
 12

 17
 0
 12

 2

 6

 2
 0
 1

 5
 0
 5

 1

 1

 0
 0
 2

 0
 0
 2

 0
 0
 2

 14
 0
 2

 13

 2

 11

 1
 0
 1

 1
 0
 1

 0
 0
 1

 0
 0
 1

 0
 0
 1

 0
 0
 1

 0
 0
 1

 0
 0
 1

 0
 0
 1

 0
 0
 1

 184
 0
 243

 184
 0
 243

 9
 0
 5

 2

 7
 0
 5

 6
 0
 1

 4

 1

 1

 0
 0
 1

 22
 0
 191

 1

 1

 1

 1
 0
 3

 1
 0
 34

 2
 0
 18

 2
 0
 68

 7
 0
 22

 1

 4

 1

 0
 0
 1

 0
 0
 1

 0
 0
 2

 0
 0
 13

 0
 0
 1

 0
 0
 3

 0
 0
 18

 0
 0
 1

 0
 0
 1

 0
 0
 2

 0
 0
 1

 0
 0
 2

 2
 0
 3

 1
 0
 2

 1

 0
 0
 1

 96
 0
 34

 1

 2

 1

 2

 1

 1

 1

 2

 1
 0
 6

 3

 2

 1

 2

 2

 7

 9
 0
 2

 5

 6

 4

 2

 15
 0
 6

 1

 1

 3

 1

 1

 1

 1

 1

 1

 1

 2
 0
 1

 3

 2

 1

 1

 1

 1

 1

 1

 1

 0
 0
 1

 0
 0
 5

 0
 0
 1

 0
 0
 4

 0
 0
 1

 0
 0
 1

 0
 0
 2

 0
 0
 1

 0
 0
 1

 0
 0
 1

 0
 0
 1

 49
 0
 9

 1

 1

 1

 1

 3

 1

 1

 12
 0
 1

 13
 0
 3

 3

 8

 2

 1

 1

 0
 0
 1

 0
 0
 3

 0
 0
 1

 718
 1
 226

 668
 0
 190

 1
 0
 3

 1

 0
 0
 1

 0
 0
 1

 0
 0
 1

 539
 0
 117

 1

 1

 1

 1

 1

 1

 1

 1

 1

 1

 1

 1

 1

 1

 1

 1

 1

 1

 1

 1

 2

 1

 2

 1

 1

 1

 2

 1

 1

 1

 3

 1
 0
 1

 1

 1

 1

 1

 2

 1

 1

 4

 1

 1

 2

 2

 1

 1

 3

 1

 2

 1

 3

 3

 1

 2

 1

 1

 3

 1

 2

 1

 1

 2

 2

 1

 2

 3

 5

 16

 5

 12

 14

 3

 2

 2

 17

 3
 0
 1

 1

 25
 0
 6

 7

 4

 8
 0
 18

 1

 12

 2

 1

 1

 3

 2

 1

 5

 1

 2

 1

 1

 14
 0
 3

 1

 1

 5

 1

 19
 0
 12

 2

 28

 12

 3
 0
 5

 3

 17

 1

 2
 0
 1

 12

 1

 1

 4
 0
 1

 5
 0
 1

 8

 3

 5

 5

 3

 1

 4

 1

 7

 3

 2

 1

 8

 1

 2

 3

 3

 7

 13

 3

 18
 0
 4

 2

 1

 2
 0
 1

 1

 2

 1

 1

 1

 1

 1

 1

 1

 1

 1

 1

 1

 1

 2

 1

 3

 1

 2

 1

 4

 1

 1

 1

 1

 1

 1

 1

 1

 1

 1

 1

 1

 1

 1

 1

 1

 1

 1

 1

 1

 1

 1

 0
 0
 1

 0
 0
 1

 0
 0
 1

 0
 0
 1

 0
 0
 1

 0
 0
 1

 0
 0
 1

 0
 0
 1

 0
 0
 1

 0
 0
 10

 0
 0
 1

 0
 0
 5

 0
 0
 1

 0
 0
 2

 0
 0
 1

 0
 0
 1

 0
 0
 2

 0
 0
 2

 0
 0
 4

 0
 0
 1

 0
 0
 1

 0
 0
 3

 0
 0
 10

 0
 0
 1

 0
 0
 2

 0
 0
 1

 0
 0
 1

 0
 0
 1

 0
 0
 3

 0
 0
 1

 22

 1

 2

 13

 4

 1

 1

 39
 0
 10

 1

 1

 1

 1

 1

 1

 1

 17

 5
 0
 4

 2

 3
 0
 2

 1

 2

 1

 1

 0
 0
 4

 6
 0
 3

 1

 1

 2

 1

 1

 0
 0
 3

 1

 1

 60
 0
 52

 1

 1

 1
 0
 2

 5

 20
 0
 9

 3

 1
 0
 1

 5

 12

 6

 2
 0
 32

 1

 1

 1

 0
 0
 1

 0
 0
 1

 0
 0
 1

 0
 0
 4

 0
 0
 1

 0
 0
 1

 0
 0
 1

 0
 0
 3

 0
 0
 3

 0
 0
 1

 0
 0
 1

 25
 0
 7

 25
 0
 7

 1

 1

 1

 1
 0
 1

 3
 0
 1

 1

 1

 1

 5

 1

 2

 1

 1

 1
 0
 1

 1

 1

 2

 0
 0
 1

 0
 0
 1

 0
 0
 1

 0
 0
 1

 16
 1
 18

 5
 1

 1

 2

 2

 0
 1

 1

 1

 6
 0
 5

 1

 1

 1
 0
 2

 3

 0
 0
 1

 0
 0
 1

 0
 0
 1

 4
 0
 4

 3

 1

 0
 0
 4

 0
 0
 2

 0
 0
 1

 0
 0
 1

 0
 0
 2

 0
 0
 2

 0
 0
 5

 0
 0
 5

 9
 0
 11

 9
 0
 11

 1

 2

 1

 1

 1
 0
 1

 1

 1

 1

 0
 0
 1

 0
 0
 1

 0
 0
 1

 0
 0
 1

 0
 0
 2

 0
 0
 1

 0
 0
 1

 0
 0
 1

 0
 0
 1

 7

 7

 7

 1

 6

 632
 0
 379

 9
 0
 1

 1

 1

 7
 0
 1

 1

 1

 1

 1

 1

 1

 1

 0
 0
 1

 1

 1

 601
 0
 374

 39
 0
 23

 1

 1

 3

 1

 1

 1

 1

 3
 0
 4

 1

 1
 0
 3

 2

 1

 1
 0
 1

 7
 0
 2

 4

 2

 2

 1

 1

 2

 2

 0
 0
 1

 0
 0
 3

 0
 0
 1

 0
 0
 2

 0
 0
 2

 0
 0
 2

 0
 0
 2

 2

 2

 49
 0
 2

 3

 13
 0
 1

 6

 4
 0
 1

 1

 5

 15

 1

 1

 3

 1

 1

 1

 9
 0
 3

 1

 1

 1

 1

 2

 1

 1

 1

 0
 0
 1

 0
 0
 1

 0
 0
 1

 242
 0
 37

 1

 1

 1

 1

 1

 1

 1

 1

 1

 1

 1

 1

 1

 1

 1

 1

 1

 1

 1
 0
 1

 1

 1

 2
 0
 1

 1

 3
 0
 2

 3

 11

 3

 6

 2

 1

 1

 15
 0
 1

 30

 1

 3

 22
 0
 8

 2
 0
 1

 63
 0
 1

 1

 19
 0
 5

 3

 2

 2

 1

 1

 2

 3

 2

 2

 1

 1

 1

 3

 1

 1

 1

 1

 1

 1

 1

 1

 0
 0
 1

 0
 0
 1

 0
 0
 1

 0
 0
 6

 0
 0
 1

 0
 0
 1

 0
 0
 1

 0
 0
 1

 0
 0
 1

 0
 0
 1

 0
 0
 2

 237
 0
 309

 1

 1

 1

 1

 1

 1

 1

 1

 1

 1

 1

 1

 1

 1

 1

 1

 1

 1

 1

 1

 1

 1
 0
 2

 2

 1

 3

 2

 5
 0
 1

 1
 0
 2

 2

 2

 2

 4

 3

 1

 5
 0
 1

 9
 0
 1

 2

 4

 1

 22
 0
 1

 1

 4

 3

 2

 19
 0
 2

 5

 33
 0
 3

 8

 1
 0
 1

 2

 3

 13

 1

 1

 1

 2

 2

 2

 2

 2

 3
 0
 1

 1

 1

 2

 1

 1

 1

 1

 1

 1

 1

 1

 1

 2

 1

 1

 1

 1

 1

 1

 1

 1

 1

 1

 1

 1

 2

 1

 1

 1

 1

 1

 0
 0
 1

 0
 0
 1

 0
 0
 2

 0
 0
 2

 0
 0
 4

 0
 0
 1

 0
 0
 2

 0
 0
 1

 0
 0
 259

 0
 0
 1

 0
 0
 2

 0
 0
 1

 0
 0
 3

 0
 0
 3

 0
 0
 1

 0
 0
 2

 0
 0
 1

 0
 0
 1

 0
 0
 1

 0
 0
 2

 0
 0
 2

 0
 0
 1

 4

 1

 1

 1

 1

 16

 1

 1

 13

 1

 22
 0
 4

 22
 0
 4

 1

 1

 2

 1

 14
 0
 3

 1

 1

 1

 0
 0
 1

 760
 1083
 1344
 93

 132
 1
 74
 68

 132
 1
 74
 68

 132
 1
 74
 68

 1

 1

 1

 1

 1

 1

 1

 1

 1

 1

 1

 2

 1

 1

 1

 1

 1

 1

 1

 1

 1

 1

 1

 1

 1

 1

 1

 1

 1

 1

 1

 1

 1

 1

 2

 1

 1

 1

 2
 0
 3

 1

 1

 3
 0
 2

 4

 1

 33

 19
 0
 7

 1

 1

 1

 2

 2

 1

 1

 1

 2

 1

 1

 1

 1

 1

 1

 1

 2

 1

 1

 1

 1

 1

 1

 1

 0
 1

 0
 0
 1

 0
 0
 1

 0
 0
 1

 0
 0
 1

 0
 0
 2

 0
 0
 1

 0
 0
 1

 0
 0
 1

 0
 0
 1

 0
 0
 2

 0
 0
 4

 0
 0
 1

 0
 0
 1

 0
 0
 5

 0
 0
 1

 0
 0
 1

 0
 0
 1

 0
 0
 1

 0
 0
 1

 0
 0
 2

 0
 0
 1

 0
 0
 15

 0
 0
 1

 0
 0
 2

 0
 0
 1

 0
 0
 1

 0
 0
 2

 0
 0
 2

 0
 0
 1

 0
 0
 2

 0
 0
 1

 0
 0
 2

 0
 0
 1

 0
 0
 0
 13

 0
 0
 0
 6

 0
 0
 0
 37

 0
 0
 0
 12

 591
 212
 235
 25

 16
 0
 2

 3

 1

 1

 1

 2

 1

 1

 9
 0
 2

 9
 0
 2

 2

 1

 1

 35
 193
 34

 34
 0
 9

 1

 1

 20

 4

 2

 1

 2

 1

 1

 1

 0
 0
 2

 0
 0
 3

 0
 0
 3

 0
 0
 1

 1

 1

 0
 193
 22

 0
 1

 0
 155
 6

 0
 37
 15

 0
 0
 1

 0
 0
 1

 0
 0
 1

 0
 0
 1

 0
 0
 1

 0
 0
 1

 0
 0
 1

 8
 0
 20

 4
 0
 17

 1
 0
 4

 1
 0
 1

 1

 1

 0
 0
 8

 0
 0
 3

 0
 0
 1

 4
 0
 1

 4
 0
 1

 0
 0
 2

 0
 0
 2

 100
 3
 23
 7

 100
 3
 23
 7

 1

 1

 1

 1

 1

 1

 1

 15

 1

 1

 1

 1

 1

 2

 1

 1

 2

 2

 5

 1

 1

 2

 3

 1

 1

 14

 2

 3

 2
 0
 2

 7

 6

 2

 1

 1

 1

 4

 1

 1

 1

 1

 1

 1

 1

 1

 0
 3

 0
 0
 1

 0
 0
 1

 0
 0
 4

 0
 0
 1

 0
 0
 1

 0
 0
 4

 0
 0
 1
 5

 0
 0
 2

 0
 0
 6

 0
 0
 0
 2

 281
 16
 71

 5
 0
 1

 2

 3
 0
 1

 222
 16
 33

 1

 1

 1

 1

 1

 1

 1

 1

 1

 1

 4

 1

 1

 1

 1

 1

 6
 0
 2

 2

 1

 6
 0
 3

 4

 1

 1
 0
 2

 2

 23

 1

 9
 0
 9

 1
 0
 1

 8

 10

 33

 9

 12
 0
 5

 1

 1

 10

 6

 1
 0
 1

 20
 0
 2

 2

 2

 1

 2

 2

 1

 1

 1

 1

 1

 5

 1

 1

 1

 1

 1

 1

 1

 1

 1

 1

 1

 1

 1

 1

 1

 1

 0
 16

 0
 0
 1

 0
 0
 1

 0
 0
 1

 0
 0
 2

 0
 0
 2

 0
 0
 1

 2
 0
 2

 1

 1

 0
 0
 2

 1

 1

 9
 0
 12

 1

 1

 1

 6
 0
 12

 7
 0
 4

 1

 5
 0
 4

 1

 34

 2

 15

 1

 16

 1

 1

 0
 0
 15

 0
 0
 1

 0
 0
 2

 0
 0
 5

 0
 0
 4

 0
 0
 3

 0
 0
 4

 0
 0
 1

 0
 0
 3

 150
 0
 85
 9

 5

 1

 1

 2

 1

 1

 1

 3

 1

 2

 85
 0
 79

 1

 1

 1

 1

 1

 1

 2

 1

 1

 1

 2

 1

 2

 1

 5

 1

 3
 0
 9

 1

 16

 1

 15
 0
 59

 1

 1

 13
 0
 1

 1

 1

 3

 1

 2

 1

 1

 1

 0
 0
 8

 0
 0
 2

 4
 0
 2

 2

 1

 1

 0
 0
 2

 52
 0
 2
 9

 1

 1

 1

 1

 1

 1

 1

 1

 1

 1

 1

 20

 1

 1

 1

 1

 3
 0
 1

 1

 2

 1

 2

 1

 1

 1

 1

 1

 1

 1

 1

 0
 0
 1

 0
 0
 0
 5

 0
 0
 0
 4

 0
 0
 2

 0
 0
 2

 1
 0
 0
 9

 1

 1

 0
 0
 0
 1

 0
 0
 0
 1

 0
 0
 0
 8

 0
 0
 0
 2

 0
 0
 0
 4

 0
 0
 0
 2

 20
 0
 6

 20
 0
 6

 13

 13

 7
 0
 6

 7
 0
 3

 0
 0
 2

 0
 0
 1

 4
 870
 987

 4
 870
 987

 1
 653
 125

 1

 0
 1

 0
 1

 0
 1

 0
 1

 0
 2

 0
 3

 0
 5

 0
 11
 10

 0
 10

 0
 4
 7

 0
 18

 0
 2

 0
 1

 0
 50
 13

 0
 372
 31

 0
 1

 0
 60
 9

 0
 62
 30

 0
 1

 0
 1

 0
 16

 0
 18

 0
 3

 0
 1

 0
 3

 0
 1

 0
 1

 0
 1

 0
 1

 0
 1

 0
 0
 1

 0
 0
 1

 0
 0
 1

 0
 0
 1

 0
 0
 11

 0
 0
 2

 0
 0
 1

 0
 0
 7

 3
 74
 850

 1

 1
 3

 1
 0
 764

 0
 1

 0
 35

 0
 1

 0
 5

 0
 3
 4

 0
 1

 0
 7
 6

 0
 15

 0
 2

 0
 1

 0
 0
 1

 0
 0
 1

 0
 0
 29

 0
 0
 42

 0
 0
 1

 0
 0
 2

 0
 12
 3

 0
 11
 3

 0
 1

 0
 131
 7

 0
 1

 0
 1

 0
 2
 3

 0
 115
 4

 0
 1

 0
 3

 0
 8

 0
 0
 2

 0
 0
 2

 2

 2

 1

 1

 1

 1

 1

 1

 1

 1

 10
 0
 39

 10
 0
 39

 1

 1

 8
 0
 6

 1

 1

 3

 1

 1

 1

 0
 0
 1

 0
 0
 1

 0
 0
 1

 0
 0
 3

 1
 0
 26

 1

 0
 0
 26

 0
 0
 5

 0
 0
 5

 0
 0
 1

 0
 0
 1

 0
 0
 1

 0
 0
 1

 0
 0
 3

 0
 0
 3

 0
 0
 1

 0
 0
 1

 0
 0
 2

 0
 0
 2

 409
 5
 82
 83

 35
 0
 16

 22
 0
 14

 1

 1

 21
 0
 14

 1

 1

 1

 2

 1

 3
 0
 3

 1

 1

 1
 0
 1

 2

 1

 1

 1

 2

 1

 1

 0
 0
 6

 0
 0
 1

 0
 0
 1

 0
 0
 2

 13
 0
 2

 12
 0
 2

 1

 2

 1

 1

 1

 1

 1

 1

 1

 1

 1

 0
 0
 1

 0
 0
 1

 1

 1

 1

 1

 1

 1

 55
 0
 15

 55
 0
 15

 55
 0
 15

 1

 1

 1

 1

 1

 1

 1

 1

 1

 1

 1

 1

 1

 1

 1

 1

 1

 3

 1

 3

 2
 0
 1

 6

 4

 1

 1

 1

 1

 1

 1

 1

 2

 1

 1

 1

 1

 2
 0
 1

 1

 1

 1

 1

 0
 0
 1

 0
 0
 1

 0
 0
 9

 0
 0
 1

 0
 0
 1

 1
 3

 1

 1

 1

 0
 3

 0
 3

 0
 3

 1
 0
 6

 1
 0
 2

 1
 0
 2

 1

 0
 0
 1

 0
 0
 1

 0
 0
 4

 0
 0
 4

 0
 0
 4

 316
 1
 45

 11
 0
 1

 4
 0
 1

 1

 1

 1

 1

 0
 0
 1

 5

 1

 2

 1

 1

 2

 1

 1

 34
 0
 2

 34
 0
 2

 1

 1

 1

 1

 1

 4

 11

 1

 1

 5

 2

 1

 1

 1

 1

 1

 0
 0
 1

 0
 0
 1

 6

 4

 1

 3

 1

 1

 1

 1

 193
 1
 31

 193
 1
 31

 1

 1

 1

 1

 1

 1

 1

 1

 1

 1

 1

 1

 1

 1

 1

 1

 1

 1

 1
 0
 1

 1

 1

 1

 1

 1

 1

 1

 1

 1

 1

 1

 1

 1

 1

 1

 1

 1

 3

 1

 2
 0
 1

 8

 1

 9

 2

 1

 6

 2

 2

 1

 1

 1

 3

 24

 1

 1

 1

 7

 6

 11

 4

 1

 1

 2

 1

 1

 1

 1

 1

 1

 1

 1

 2

 1

 2

 1

 1

 1

 1

 5

 3

 1

 2

 1

 1

 1

 1
 1

 2

 2

 1

 1

 1

 1

 1

 1

 1

 2

 1

 1

 1

 1

 1

 1

 1

 1

 1

 1

 0
 0
 1

 0
 0
 1

 0
 0
 1

 0
 0
 1

 0
 0
 1

 0
 0
 2

 0
 0
 1

 0
 0
 1

 0
 0
 3

 0
 0
 1

 0
 0
 2

 0
 0
 5

 0
 0
 1

 0
 0
 1

 0
 0
 3

 0
 0
 2

 0
 0
 1

 0
 0
 1

 6

 3

 1

 1

 1

 3

 3

 1
 0
 3

 1
 0
 3

 1

 0
 0
 3

 65
 0
 8

 9

 7

 2

 1

 1

 54
 0
 8

 1

 1

 1

 1

 1

 2

 1

 2

 1

 1

 3

 5

 1

 6

 2

 17

 1

 1

 1

 2

 1

 1

 1

 0
 0
 1

 0
 0
 1

 0
 0
 2

 0
 0
 1

 0
 0
 3

 1

 1

 0
 1
 0
 83

 0
 1
 0
 49

 0
 1
 0
 37

 0
 1

 0
 0
 0
 1

 0
 0
 0
 36

 0
 0
 0
 12

 0
 0
 0
 12

 0
 0
 0
 34

 0
 0
 0
 34

 0
 0
 0
 34

 28
 299
 57
 1

 28
 299
 57
 1

 27
 299
 56
 1

 22
 0
 4

 1

 19
 0
 1

 2
 0
 2

 0
 0
 1

 2
 299
 51
 1

 2

 0
 1

 0
 1

 0
 2

 0
 17
 19

 0
 9

 0
 1

 0
 149
 0
 1

 0
 92
 24

 0
 25
 5

 0
 2

 0
 0
 3

 3
 0
 1

 3
 0
 1

 1
 0
 1

 1

 1

 0
 0
 1

 0
 0
 1

 1226
 1053
 1685
 33

 8
 0
 12
 22

 6
 0
 12

 1
 0
 9

 1

 0
 0
 1

 0
 0
 2

 0
 0
 6

 5
 0
 3

 1
 0
 1

 3
 0
 1

 1

 0
 0
 1

 2
 0
 0
 22

 2

 1

 1

 0
 0
 0
 7

 0
 0
 0
 7

 0
 0
 0
 15

 0
 0
 0
 15

 8
 0
 15

 3
 0
 4

 1

 1

 2

 2

 0
 0
 4

 0
 0
 4

 1

 1

 1

 2
 0
 6

 1
 0
 6

 1

 0
 0
 1

 0
 0
 1

 0
 0
 2

 0
 0
 1

 0
 0
 1

 1

 1

 2

 2

 1

 1

 0
 0
 2

 0
 0
 2

 0
 0
 2

 0
 0
 3

 0
 0
 3

 0
 0
 2

 0
 0
 1

 5
 0
 5

 5
 0
 2

 5
 0
 2

 1

 2

 2

 0
 0
 2

 0
 0
 1

 0
 0
 1

 0
 0
 1

 0
 0
 2

 0
 0
 2

 0
 0
 2

 227
 5
 126
 11

 227
 5
 126
 11

 11
 0
 18

 1

 1

 1

 3

 4
 0
 2

 1

 0
 0
 8

 0
 0
 4

 0
 0
 1

 0
 0
 3

 182
 5
 42
 11

 1

 1

 1

 1

 1

 1

 1

 1

 2

 1

 1

 1

 1

 1

 1

 3

 1

 1

 1

 1

 1

 1

 69

 14

 1

 1

 6
 0
 1

 1

 25

 2

 1

 1

 1
 5
 18
 10

 2

 1

 1

 1

 1
 0
 2

 1

 1

 2

 2

 2

 1

 3

 9

 1

 3

 1

 1

 1

 1

 0
 0
 2

 0
 0
 4

 0
 0
 1

 0
 0
 1

 0
 0
 1

 0
 0
 1

 0
 0
 1

 0
 0
 1

 0
 0
 1

 0
 0
 1

 0
 0
 5

 0
 0
 1

 0
 0
 1

 0
 0
 0
 1

 8
 0
 8

 8

 0
 0
 6

 0
 0
 2

 1
 0
 3

 1
 0
 3

 17

 15

 2

 1

 1

 7
 0
 43

 7
 0
 43

 0
 0
 1

 0
 0
 1

 0
 0
 1

 0
 0
 1

 0
 0
 8

 0
 0
 8

 0
 0
 1

 0
 0
 1

 0
 0
 1

 0
 0
 1

 136
 3
 268

 136
 3
 268

 136
 3
 268

 1

 1

 1

 3

 1

 1

 1

 1

 2

 1

 1
 0
 1

 1

 1

 1

 2
 0
 1

 2

 2

 2

 1

 1

 1
 0
 3

 1

 29

 1

 2

 4

 1

 1

 2

 2

 5

 4

 2

 1

 1

 1

 4

 7

 1
 3
 2

 1

 1

 1

 8

 1

 1

 1

 1

 1

 1

 1

 1

 2

 2

 1

 1

 1

 1

 1

 1

 1

 1

 1

 1

 1

 1

 1

 1

 1

 1

 0
 0
 2

 0
 0
 1

 0
 0
 1

 0
 0
 1

 0
 0
 1

 0
 0
 1

 0
 0
 1

 0
 0
 1

 0
 0
 1

 0
 0
 1

 0
 0
 2

 0
 0
 1

 0
 0
 1

 0
 0
 2

 0
 0
 1

 0
 0
 1

 0
 0
 2

 0
 0
 1

 0
 0
 216

 0
 0
 2

 0
 0
 1

 0
 0
 1

 0
 0
 1

 0
 0
 1

 0
 0
 1

 0
 0
 1

 0
 0
 1

 0
 0
 3

 0
 0
 3

 0
 0
 1

 0
 0
 1

 0
 0
 1

 0
 0
 1

 0
 0
 1

 0
 0
 1

 0
 0
 1

 0
 0
 1

 8
 0
 21

 4
 0
 9

 3
 0
 7

 1

 1

 1

 0
 0
 1

 0
 0
 1

 0
 0
 2

 0
 0
 1

 0
 0
 2

 1
 0
 2

 1

 0
 0
 1

 0
 0
 1

 4
 0
 12

 4
 0
 12

 1

 1

 1

 1

 0
 0
 2

 0
 0
 1

 0
 0
 6

 0
 0
 1

 0
 0
 2

 4
 0
 5

 4
 0
 5

 3
 0
 1

 1

 2

 0
 0
 1

 1
 0
 1

 1
 0
 1

 0
 0
 3

 0
 0
 2

 0
 0
 1

 15
 0
 66

 11
 0
 64

 4
 0
 2

 1

 1

 1

 1

 0
 0
 2

 7
 0
 62

 1

 1

 1

 1

 1

 1

 1

 0
 0
 1

 0
 0
 1

 0
 0
 1

 0
 0
 1

 0
 0
 1

 0
 0
 1

 0
 0
 1

 0
 0
 21

 0
 0
 25

 0
 0
 1

 0
 0
 1

 0
 0
 1

 0
 0
 6

 2
 0
 2

 1

 1

 1
 0
 2

 1

 0
 0
 2

 2

 2

 2

 3
 977
 94

 3
 977
 94

 1
 88

 1

 0
 2

 0
 2

 0
 1

 0
 1

 0
 2

 0
 1

 0
 26

 0
 17

 0
 24

 0
 5

 0
 1

 0
 1

 0
 1

 0
 4

 1
 1

 1

 0
 1

 1
 871
 92

 1

 0
 2

 0
 1

 0
 1

 0
 1

 0
 1

 0
 2

 0
 2

 0
 1

 0
 1

 0
 5
 7

 0
 763
 77

 0
 7

 0
 10
 1

 0
 3

 0
 7

 0
 1

 0
 26

 0
 4

 0
 1

 0
 1

 0
 2

 0
 1

 0
 1

 0
 1

 0
 1

 0
 3

 0
 14

 0
 1

 0
 1

 0
 1

 0
 1

 0
 3

 0
 1

 0
 0
 1

 0
 0
 4

 0
 0
 2

 0
 17
 2

 0
 1

 0
 2

 0
 9

 0
 4
 1

 0
 1

 0
 0
 1

 487
 0
 876

 174
 0
 729

 69
 0
 172

 1

 1

 1

 1

 3

 1

 9

 1

 3

 1

 7
 0
 6

 9

 1

 1
 0
 31

 6

 3

 3
 0
 63

 6
 0
 1

 1
 0
 2

 1

 1

 1

 1

 1

 1

 1

 1

 1

 1

 0
 0
 1

 0
 0
 1

 0
 0
 1

 0
 0
 2

 0
 0
 3

 0
 0
 29

 0
 0
 1

 0
 0
 1

 0
 0
 1

 0
 0
 1

 0
 0
 3

 0
 0
 4

 0
 0
 1

 0
 0
 1

 0
 0
 3

 0
 0
 2

 0
 0
 1

 0
 0
 1

 0
 0
 4

 0
 0
 1

 0
 0
 3

 0
 0
 1

 0
 0
 1

 0
 0
 1

 0
 0
 1

 4
 0
 1

 4

 0
 0
 1

 1
 0
 3

 1

 0
 0
 1

 0
 0
 1

 0
 0
 1

 2
 0
 16

 1
 0
 7

 1

 0
 0
 6

 0
 0
 2

 0
 0
 1

 98
 0
 532

 1

 1

 1

 1

 1

 10
 0
 1

 7
 0
 3

 45
 0
 10

 26
 0
 9

 1

 1

 1

 1

 1

 0
 0
 1

 0
 0
 1

 0
 0
 505

 0
 0
 2

 0
 0
 5

 0
 0
 2

 0
 0
 2

 0
 0
 1

 313
 0
 147

 1

 1

 44
 0
 3

 1

 1

 3

 1

 3

 1

 1

 6

 18

 2
 0
 2

 1
 0
 1

 2

 2

 1

 1

 5
 0
 6

 1

 1

 1
 0
 1

 1

 1

 0
 0
 1

 0
 0
 3

 0
 0
 1

 261
 0
 138

 1

 1

 1

 1

 1

 1

 2

 1

 1

 2
 0
 1

 2

 1

 2

 1

 1

 1

 2

 2

 1

 1

 2

 1

 1

 1

 2

 2

 1

 1

 2
 0
 2

 2

 54
 0
 1

 1

 58

 31
 0
 3

 13
 0
 4

 1

 2
 0
 3

 1

 30
 0
 15

 6
 0
 1

 1

 8

 1

 1

 1

 1

 1

 1

 1

 1

 1

 1

 1

 1

 1

 1

 0
 0
 2

 0
 0
 1

 0
 0
 1

 0
 0
 2

 0
 0
 44

 0
 0
 11

 0
 0
 4

 0
 0
 9

 0
 0
 8

 0
 0
 7

 0
 0
 1

 0
 0
 1

 0
 0
 1

 0
 0
 3

 0
 0
 1

 0
 0
 1

 0
 0
 5

 0
 0
 1

 0
 0
 1

 0
 0
 2

 0
 0
 2

 2

 1

 1

 1

 1

 1

 1

 1
 0
 4

 1
 0
 4

 1
 0
 4

 1
 0
 2

 0
 0
 2

 15
 0
 15

 15
 0
 15

 13
 0
 11

 13
 0
 11

 2
 0
 4

 1

 1

 0
 0
 4

 308
 2
 153

 10
 0
 17

 1
 0
 3

 1

 0
 0
 3

 6
 0
 3

 1

 1

 1

 1

 1

 1

 0
 0
 2

 0
 0
 1

 3

 1

 1

 1

 0
 0
 2

 0
 0
 2

 0
 0
 2

 0
 0
 2

 0
 0
 2

 0
 0
 1

 0
 0
 1

 0
 0
 3

 0
 0
 3

 0
 0
 2

 0
 0
 2

 296
 2
 134

 24
 0
 6

 11
 0
 3

 2

 1

 2

 1

 4

 3
 0
 1

 0
 0
 1

 0
 0
 1

 2

 1

 1

 18
 0
 13

 1

 1

 1

 11
 0
 5

 3
 0
 1

 1

 0
 0
 6

 0
 0
 1

 30
 0
 5

 30
 0
 2

 0
 0
 3

 3

 3

 26
 0
 6

 1

 1

 24
 0
 6

 35
 0
 25

 1

 1

 1

 1
 0
 1

 1

 2

 1

 4

 11
 0
 2

 1
 0
 13

 3

 1
 0
 1

 3
 0
 8

 2

 1

 1

 30
 1
 5

 1

 3

 6
 0
 3

 14

 3
 0
 2

 1

 1

 1

 0
 1

 6
 0
 22

 1

 1

 1

 1
 0
 1

 1

 1

 0
 0
 5

 0
 0
 2

 0
 0
 6

 0
 0
 8

 5
 0
 3

 1

 1

 1

 1

 1

 0
 0
 1

 0
 0
 2

 1

 1

 22
 0
 25

 1

 7
 0
 2

 2

 1

 10

 1
 0
 2

 0
 0
 2

 0
 0
 1

 0
 0
 6

 0
 0
 1

 0
 0
 4

 0
 0
 6

 0
 0
 1

 4

 1

 1

 2

 1

 1

 78
 1
 18

 1

 1

 1

 1

 1

 1

 2

 2

 5
 0
 4

 2

 1

 1

 3

 2

 2

 1
 0
 1

 29

 2
 0
 1

 3

 4

 2
 0
 1

 2

 2

 1
 0
 1

 1

 1

 1

 1

 1

 1

 0
 1

 0
 0
 1

 0
 0
 1

 0
 0
 1

 0
 0
 1

 0
 0
 1

 0
 0
 1

 0
 0
 1

 0
 0
 1

 0
 0
 2

 11
 0
 6

 1

 10
 0
 6

 2
 0
 2

 2
 0
 2

 1
 0
 1

 1

 0
 0
 1

 0
 66
 23

 0
 66
 23

 0
 1

 0
 1

 0
 65
 23

 0
 1

 0
 2

 0
 23

 0
 33
 18

 0
 6
 4

 0
 0
 1

 0
 0
 2

 0
 0
 2

 0
 0
 2

 0
 0
 2

 4

 4

 4

 4

 1

 2

 1

 154
 0
 28
 172

 154
 0
 28
 172

 154
 0
 28
 172

 154
 0
 28
 172

 1

 1

 1

 1

 1

 1

 1

 1

 1

 1

 1

 1

 1

 1
 0
 1

 1

 2

 1

 1

 1

 1

 1

 1

 1

 1

 5

 1

 4
 0
 2

 1

 1

 1

 4

 1

 2

 2

 1

 12
 0
 1

 2

 9

 3

 3

 2

 5

 5
 0
 1

 2

 1

 1

 1

 1

 1

 1

 1

 6

 3

 1

 3

 10

 1

 1

 1

 1

 1

 1

 1

 3

 1

 1

 2

 1

 1

 1

 1

 1

 1

 1

 1

 1

 1

 1

 1

 1

 1

 1

 1

 2

 1

 0
 0
 1

 0
 0
 3

 0
 0
 1

 0
 0
 1

 0
 0
 1

 0
 0
 1
 171

 0
 0
 1

 0
 0
 3

 0
 0
 1

 0
 0
 3

 0
 0
 4

 0
 0
 2

 0
 0
 1

 0
 0
 0
 1

 16
 441
 13

 16
 441
 13

 16
 441
 13

 16
 441
 13

 3
 2

 1

 2

 0
 2

 13
 439
 13

 1

 3

 3

 4

 1

 1

 0
 1

 0
 1

 0
 1

 0
 1

 0
 10

 0
 1

 0
 15

 0
 2

 0
 1

 0
 1

 0
 1

 0
 1

 0
 1

 0
 33

 0
 15

 0
 15

 0
 1

 0
 1

 0
 3

 0
 1

 0
 22

 0
 8

 0
 2
 1

 0
 1

 0
 5
 2

 0
 120
 6

 0
 43

 0
 15

 0
 2

 0
 1

 0
 43

 0
 5

 0
 4

 0
 16
 2

 0
 2

 0
 6

 0
 14

 0
 17

 0
 1

 0
 1

 0
 1
 2

 0
 1

 0
 1

 0
 1

 0
 1

 12
 53

 12
 53

 1

 1

 1

 1

 1

 1

 1

 1

 10
 53

 10
 53

 5
 53

 1

 3

 1

 0
 5

 0
 39

 0
 9

 3

 2

 1

 2

 1

 1

 376
 1
 60
 7

 58
 0
 6

 58
 0
 6

 58
 0
 6

 23
 0
 1

 1

 1

 2

 5

 1

 12

 1

 0
 0
 1

 35
 0
 5

 1

 1

 1

 1

 2
 0
 1

 2

 1

 2

 2

 2

 1

 8

 1

 2

 1

 1

 1

 1

 2

 1

 1

 0
 0
 1

 0
 0
 1

 0
 0
 1

 0
 0
 1

 159
 0
 25

 154
 0
 23

 154
 0
 23

 154
 0
 23

 1

 1

 1

 1

 1

 1

 1

 1

 1

 1

 1

 1

 1

 1

 1

 1

 1

 1

 1

 2

 1

 1

 1

 1

 1

 1

 1

 1

 1

 2

 8

 2
 0
 2

 1

 11

 1

 1

 1

 1

 1

 1

 2

 1

 1

 4

 1

 2

 1

 2

 2

 2

 1
 0
 2

 1

 1

 3

 2

 2

 4

 4

 1

 6
 0
 1

 1

 8

 1

 1

 1

 1

 1

 2

 1

 1

 1

 4

 1

 2

 2

 1
 0
 1

 3

 3

 2

 1

 2

 1

 1

 1

 2

 1

 1

 1

 1

 1

 1

 0
 0
 1

 0
 0
 1

 0
 0
 1

 0
 0
 1

 0
 0
 1

 0
 0
 1

 0
 0
 1

 0
 0
 4

 0
 0
 2

 0
 0
 1

 0
 0
 1

 0
 0
 1

 0
 0
 1

 5
 0
 2

 5
 0
 2

 5
 0
 2

 1

 1

 1

 2

 0
 0
 1

 0
 0
 1

 25
 0
 6

 25
 0
 6

 25
 0
 6

 25
 0
 6

 1

 1

 1

 1

 1

 1

 1

 1

 1

 1

 1

 1

 1

 1

 1

 1

 1
 0
 1

 2
 0
 1

 1

 1

 1

 1

 1

 1

 0
 0
 1

 0
 0
 1

 0
 0
 1

 0
 0
 1

 1
 0
 3

 1
 0
 3

 1
 0
 3

 1
 0
 3

 1
 0
 1

 0
 0
 1

 0
 0
 1

 70
 0
 9

 70
 0
 9

 70
 0
 9

 70
 0
 9

 1

 1

 1

 1

 1

 2

 1

 1

 1

 1

 1

 1

 1

 1

 1

 1

 2

 1

 3

 5

 1

 1

 2

 2

 3

 1

 3

 1

 1

 1

 1

 1

 2

 1

 1

 1

 2

 1

 1

 1

 1

 1

 1

 1

 1

 1

 1

 1

 1

 1

 1

 1

 1

 1

 0
 0
 1

 0
 0
 1

 0
 0
 1

 0
 0
 1

 0
 0
 1

 0
 0
 1

 0
 0
 1

 0
 0
 1

 0
 0
 1

 63
 1
 11
 7

 63
 1
 11
 7

 63
 1
 11
 7

 13

 1

 7

 1

 4

 24
 0
 4

 1

 1

 1

 1

 12

 5

 1

 2

 0
 0
 1

 0
 0
 1

 0
 0
 1

 0
 0
 1

 10
 1
 5

 1

 1

 1

 3

 1

 1

 1

 1

 0
 1

 0
 0
 1

 0
 0
 3

 0
 0
 1

 1

 1

 15
 0
 1

 1

 1

 2

 1

 2

 2

 1

 1

 1

 1

 1

 1

 0
 0
 1

 0
 0
 1

 0
 0
 1

 0
 0
 0
 7

 0
 0
 0
 1

 0
 0
 0
 1

 0
 0
 0
 5

 0
 1821
 139

 0
 1821
 139

 0
 1821
 139

 0
 1821
 139

 0
 1821
 139

 0
 1

 0
 1

 0
 1

 0
 1

 0
 1

 0
 1

 0
 1

 0
 1

 0
 1

 0
 1

 0
 1

 0
 1

 0
 1

 0
 1

 0
 8

 0
 1

 0
 1

 0
 1

 0
 1

 0
 1

 0
 1

 0
 1

 0
 1

 0
 5

 0
 3

 0
 1

 0
 1

 0
 4
 2

 0
 3

 0
 4

 0
 34
 1

 0
 7

 0
 8
 1

 0
 34

 0
 12

 0
 8

 0
 15

 0
 52
 2

 0
 231
 50

 0
 2

 0
 11
 1

 0
 85
 5

 0
 134
 1

 0
 459
 23

 0
 184
 2

 0
 91
 4

 0
 6

 0
 164
 3

 0
 8

 0
 1

 0
 64

 0
 74

 0
 25
 3

 0
 5

 0
 1

 0
 1

 0
 1

 0
 2

 0
 10

 0
 1

 0
 2

 0
 1

 0
 1

 0
 1

 0
 4

 0
 1

 0
 4

 0
 2

 0
 1

 0
 1

 0
 1

 0
 3
 1

 0
 1

 0
 1

 0
 1

 0
 1

 0
 1

 0
 1

 0
 2

 0
 2

 0
 1

 0
 1

 0
 1

 0
 1

 0
 1

 0
 1

 0
 1

 0
 1

 0
 0
 1

 0
 0
 1

 0
 0
 1

 0
 0
 1

 0
 0
 1

 0
 0
 2

 0
 0
 1

 0
 0
 1

 0
 0
 2

 0
 0
 1

 0
 0
 1

 0
 0
 2

 0
 0
 4

 0
 0
 4

 0
 0
 2

 0
 0
 1

 0
 0
 2

 0
 0
 1

 0
 0
 2

 0
 0
 1

 0
 0
 1

 0
 0
 1

 0
 0
 1

 0
 0
 2

 0
 0
 1

 0
 0
 1

 0
 0
 1

 0
 56
 2

 0
 56
 2

 0
 56
 2

 0
 56
 2

 0
 56
 2

 0
 43

 0
 13
 2

 0
 38
 3
 1

 0
 38
 3
 1

 0
 38
 3
 1

 0
 38
 3
 1

 0
 38
 2

 0
 10
 1

 0
 27

 0
 1

 0
 0
 1

 0
 0
 1

 0
 0
 1

 0
 0
 0
 1

 0
 0
 0
 1
