# Supplementary material for: Using patterns of shared taxa to infer bacterial dispersal in human living environment in urban and rural areas
Source: Appl Environ Microbiol. 2024 Sep 4;90(10):e00903-24. doi: 10.1128/aem.00903-24 (PMC11498140; doi:10.1128/aem.00903-24)
Supplement: Supplemental material — Figures S1 to S8; Tables S1a to S1c. [file aem.00903-24-s0002.pdf]

Figure S1. Flowchart of GLMM analyses and variable selection

Three data types: saliva, skin and feces
Three timepoins: spring, autumn and winter

Explanatory variables
#1 Built environment
#2 Inside pets
#3 Outdoor mean
#4 Gardening
#5 Real mat days
#6 Number of persons
#7 Handwashing

1. Initial models

A $$\frac{\text{Shared ASVs in mat \& human sample}}{\text{Bacterial richness in human sample}} \sim \text{timepoint}$$

- ➢ Model details given in supplement

B $$\frac{\text{Shared ASVs in mat \& human sample}}{\text{Bacterial richness in human sample}} \sim \text{timepoint} * \text{explanatory variable \#n}$$

- ➢ If interaction significant, model details given in supplement
- ➢ If interaction not significant, model C without interaction was built

C $$\frac{\text{Shared ASVs in mat \& human sample}}{\text{Bacterial richness in human sample}} \sim \text{timepoint} + \text{explanatory variable \#n}$$

- ➢ Model details given in supplement

D Redo with timepoints separately

- ➢ None significant
- ➢ Model details not shown

2. Forward selection

- ➢ Final models presented in manuscript

All available timepoints in the model

- ➢ Timepoint always included as an explanatory variable
- ➢ Explanatory variables offered without interaction if it was not significant in B above
- ➢ Explanatory variables offered with interaction with timepoint if it was significant in B above

Altogether three models: Saliva (incl. spring, autumn & winter), skin (incl. spring & autumn) and feces (incl. spring, autumn & winter)

Separate models for each timepoint

- ➢ All explanatory variables offered to the selection
- ➢ Fecal data in autumn not run because of low number of observations

Altogether seven models: Saliva spring, saliva autumn, saliva winter, skin spring, skin autumn, feces spring and feces winter

Figure S2. Principal coordinate analysis axes 1 and 3.

Principal coordinate analysis for all sample matrices (mat=green, saliva=blue, skin=brown, feces=black) in the spring data. Community data was processed using three different metrics: presence-absence data with Bray-Curtis index (i.e. Sørensen index), abundance data with Bray-Curtis index and Hellinger transformation with Euclidean distance. Only those study subjects that had all four sample matrices available are included and thus number of study subjects is 19.

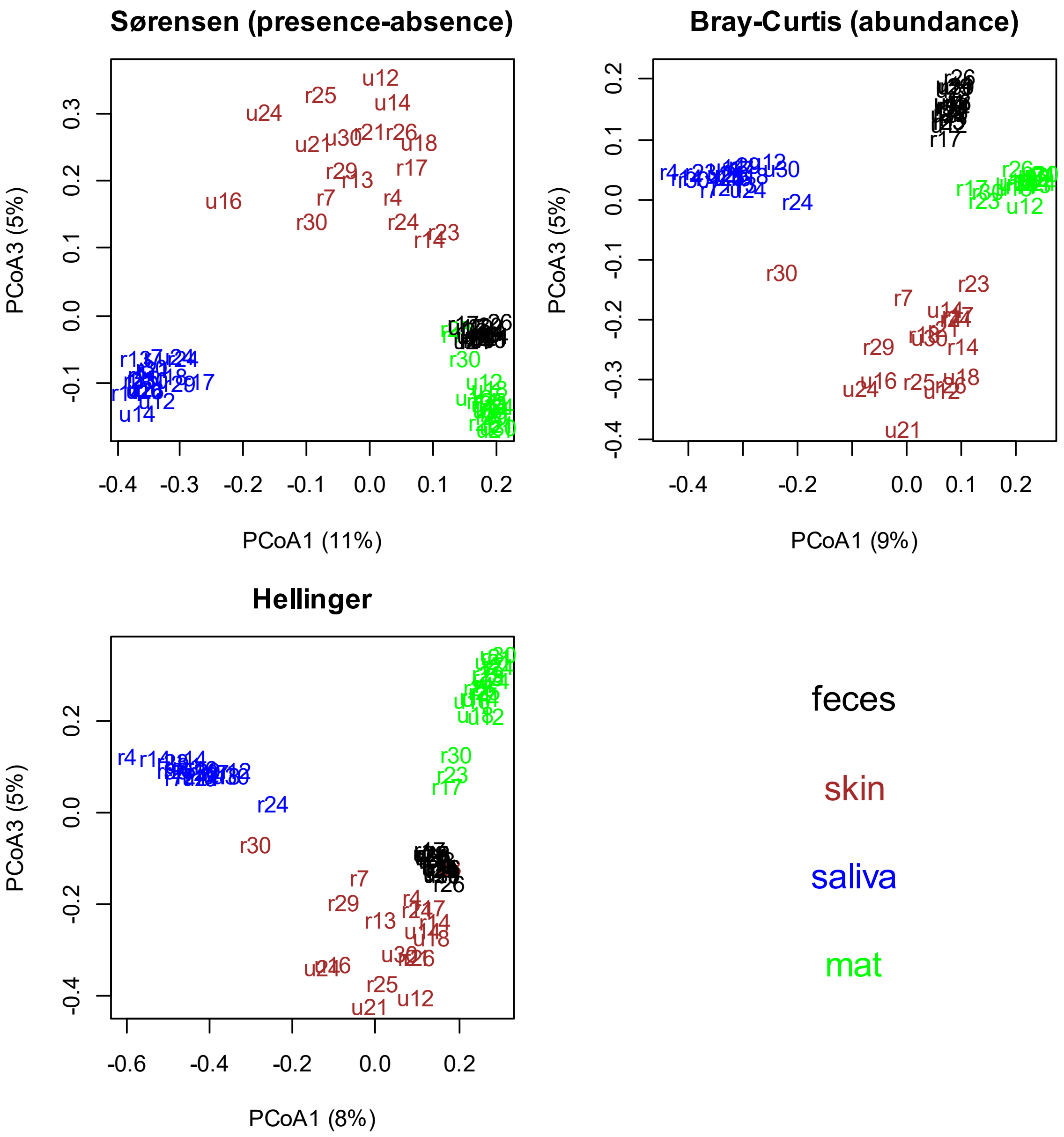

Figure S3. Dot-plot showing variation in the proportion of shared ASVs

Dot-plot shows variation in the proportion of shared ASVs in each of the datasets. Red lines show means and standard deviations.

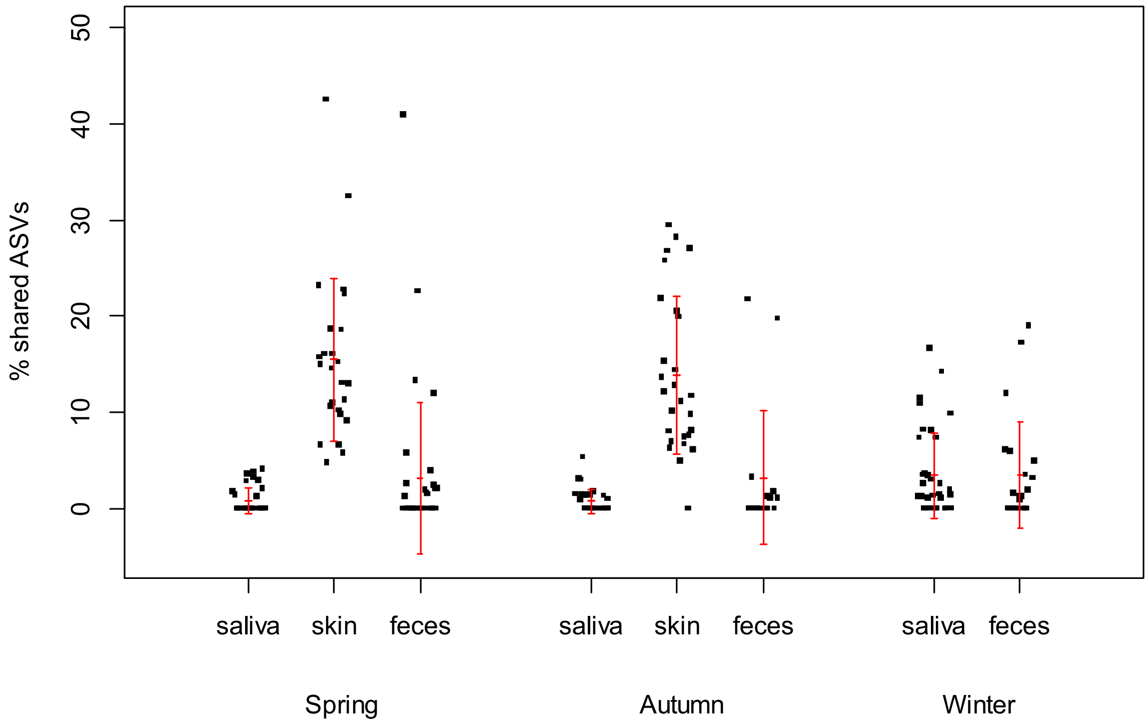

Figure S4. Model diagnostic plots for final saliva data model including all timepoints

Default plots produced by R package DHARMa (A) show some indication of quantile deviations in residuals. Same is true when residuals are plotted against the built environment (B) and outdoor mean (C) but when residuals are plotted against timepoint (D) no significant patterns are detected.

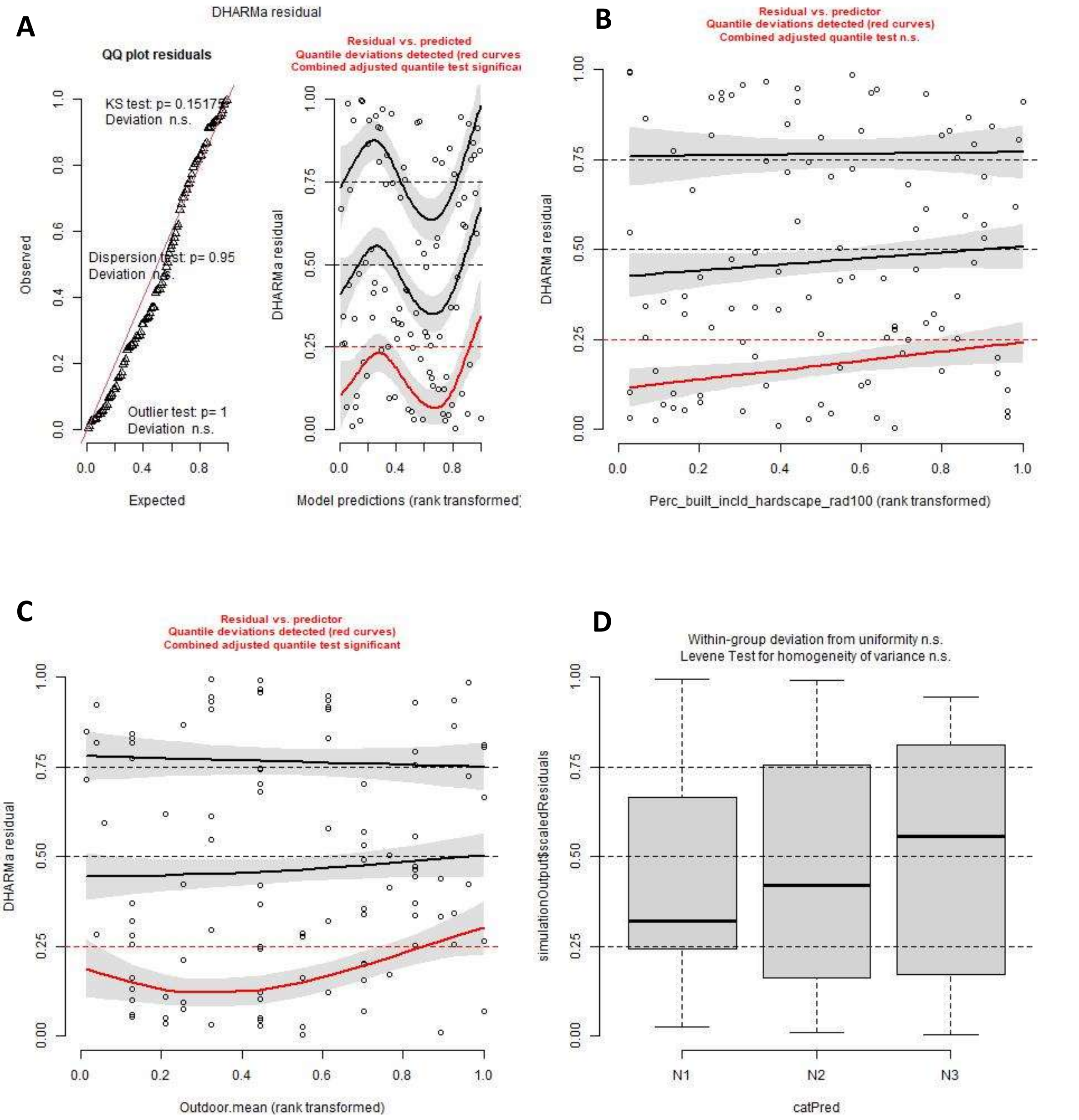

Figure S5. Model diagnostic plots for final saliva data model in winter

Default plots produced by R package DHARMa (A) show quantile deviations but when residuals are plotted against predictors (B and C) no significant pattern is detected.

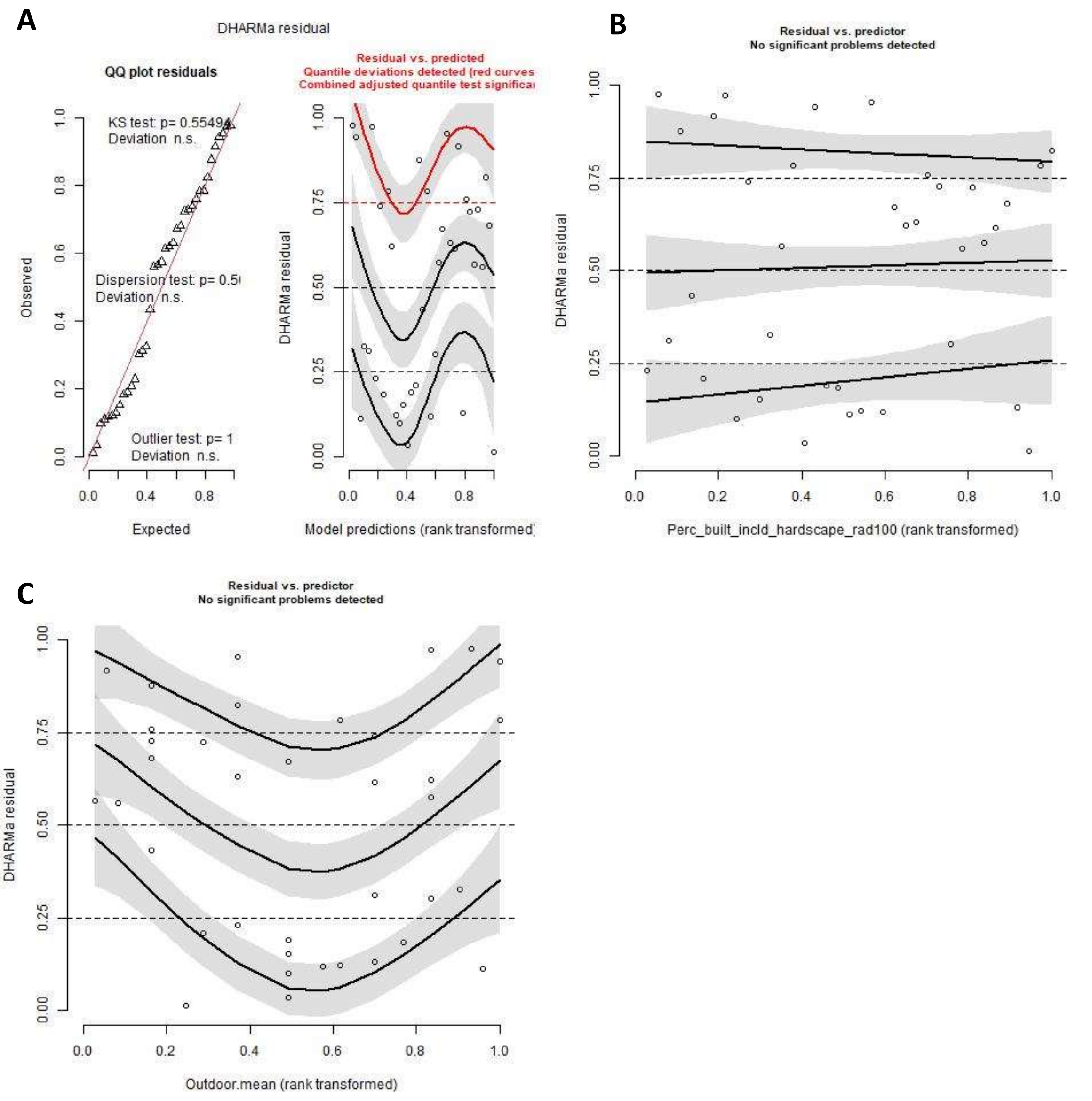

Figure S6. Model diagnostic plots for final skin data model including all timepoints

Default plots of R package DHARMa (A) show no significant patterns in residuals. When residuals are plotted against built environment (B), quantile deviations are detected. For real.mat.days (C), gardening (D) and timepoint (E), no significant patterns are detected.

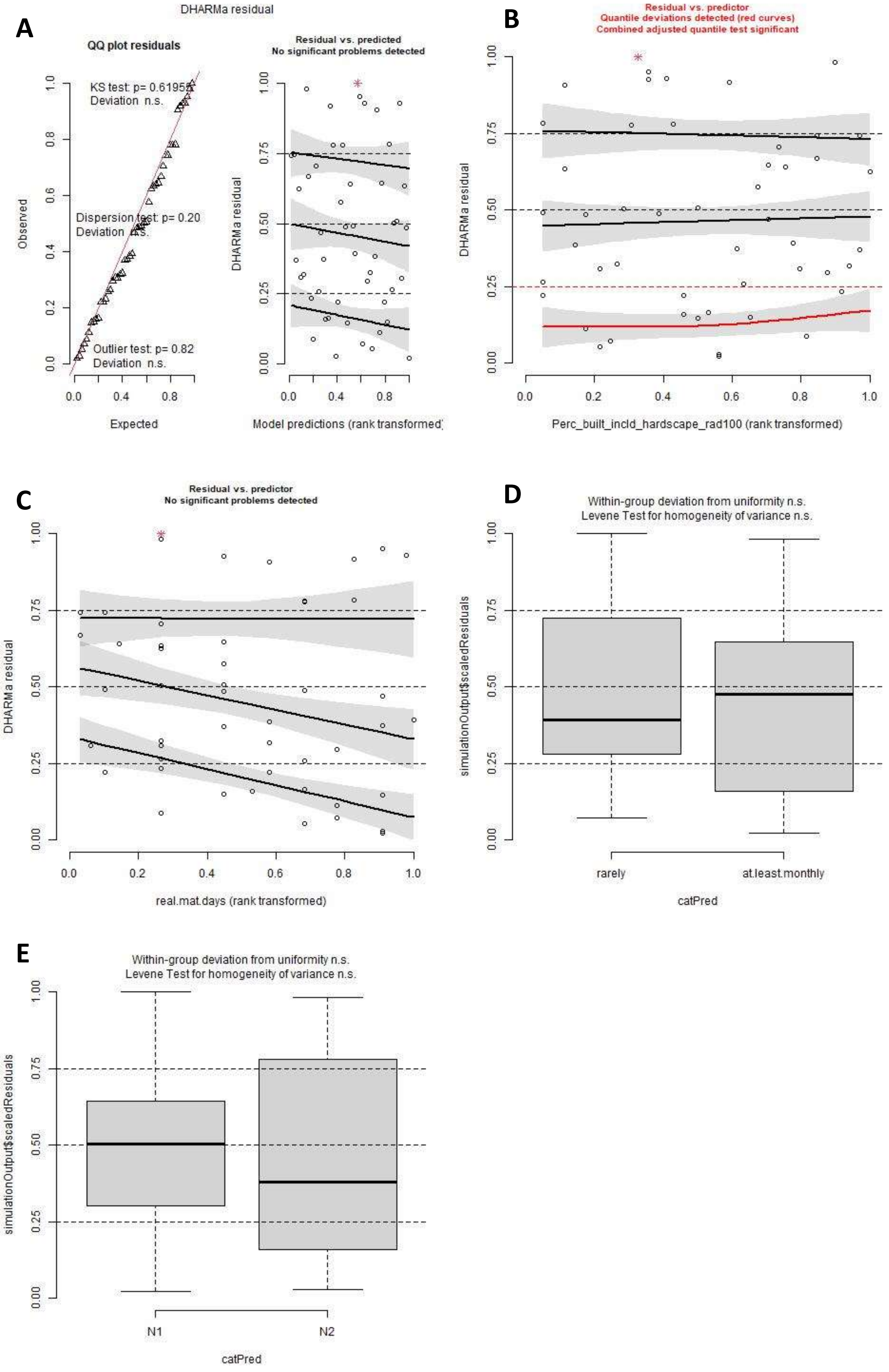

Figure S7. Model diagnostic plots for final skin data model in spring

Default plots produced by R package DHARMa show no significant patterns in residuals.

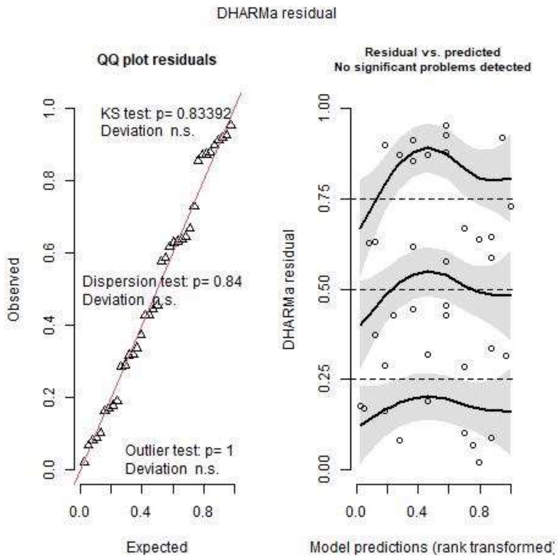

Figure S8. Model diagnostic plots for final fecal data model for all timepoints

Default plots produced by R package DHARMa (A) show that quantile deviations and dispersion test are significant. When residuals are plotted against categorical variable of person number (B) withing-group deviations from uniformity is significant but when residuals are plotted against timepoint (C), no significant pattern is detected.

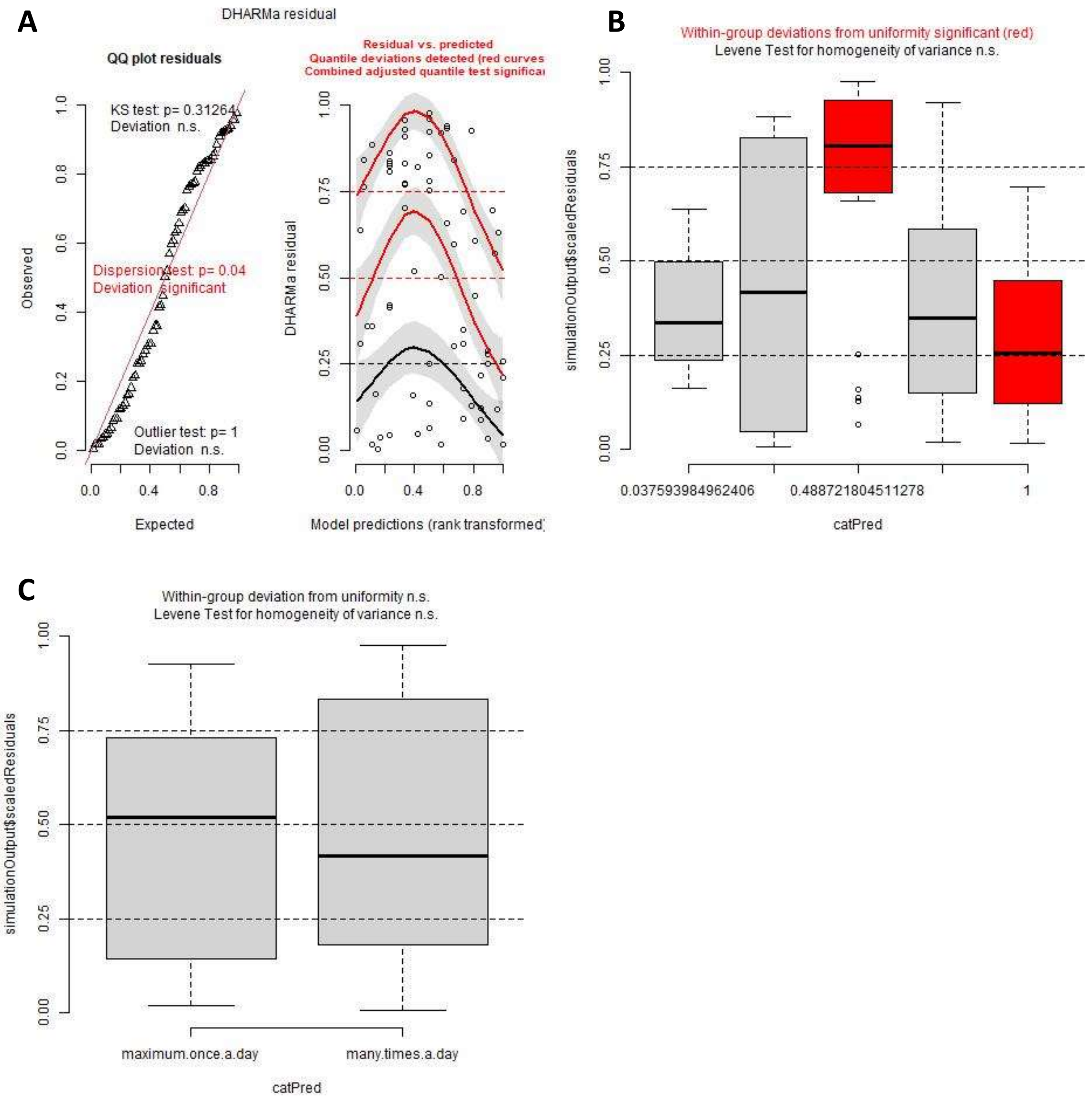

Table S1a. Model details for initial GLMM for saliva data.

Dependent variable is the proportion of ASVs in saliva sample that were also detected in the mat sample of the given study subject. Each explanatory variable was tested for interaction with timepoint. If the interaction was not significant (data not shown), a new model without interaction was built. Timepoints are S=spring, A=autumn and W=winter. P stars: * = p<0.05, ** =p<0.01, ***=p<0.001. N=number of observations in the given model. DHARMa was conducted to inspect model quality, see methods for details. Numbers denote following warnings: 1) quantile deviations detected, 2) KS test: deviation significant, 3) dispersion test: deviation significant, 4) within-group deviations from uniformity significant. Variable "number.of.persons" refer to the number of residents/visitors during the time doormat was installed and "real.mat.days" is the number of days the mat was effectively collecting the material.

Model tested	Fixed effects	Estimate	Std. Error	z value	Pr(>|z|)	p stars	N	DHARMa
timepoint	(Intercept)	-5.142	0.265	-19.406	< 0.001	***	104	2
	timepointA	0.074	0.263	0.281	0.779			
	timepointW	1.077	0.208	5.165	< 0.001	***		
built + timepoint	(Intercept)	-5.934	0.402	-14.759	< 0.001	***	104	1, 2
	Built	0.019	0.007	2.737	0.006	**		
	timepointA	0.106	0.263	0.403	0.687			
	timepointW	1.054	0.208	5.064	< 0.001	***		
pets + timepoint	(Intercept)	-4.935	0.260	-18.961	< 0.001	***	104	2, 4
	petsTRUE	-1.615	0.654	-2.468	0.014	*		
	timepointA	0.090	0.262	0.343	0.732			
	timepointW	1.082	0.208	5.204	< 0.001	***		
outdoor + timepoint	(Intercept)	-3.855	0.459	-8.396	< 0.001	***	104	1
	outdoor	-1.252	0.408	-3.073	0.002	**		
	timepointA	0.090	0.261	0.345	0.730			
	timepointW	1.093	0.207	5.289	< 0.001	***		
gardening + timepoint	(Intercept)	-4.690	0.310	-15.136	< 0.001	***	104	ok
	gardeningMONTHLY	-0.892	0.399	-2.238	0.025	*		
	timepointA	0.016	0.267	0.061	0.951			
	timepointW	1.070	0.211	5.084	< 0.001	***		
real.mat.days + timepoint	(Intercept)	-5.802	0.499	-11.627	< 0.001	***	103	1, 2
	real.mat.days	0.043	0.031	1.388	0.165			
	timepointA	-0.033	0.313	-0.106	0.916			
	timepointW	1.096	0.226	4.858	< 0.001	***		
number.of.persons + timepoint	(Intercept)	-4.364	0.459	-9.505	< 0.001	***	103	1, 2
	number.of.persons	-0.260	0.119	-2.187	0.029	*		
	timepointA	0.252	0.274	0.921	0.357			
	timepointW	1.171	0.219	5.362	< 0.001	***		
handwashing + timepoint	(Intercept)	-5.667	0.452	-12.541	< 0.001	***	101	2
	handwashingOFTEN	0.765	0.474	1.613	0.107			
	timepointA	-0.004	0.274	-0.013	0.990			
	timepointW	0.968	0.213	4.549	< 0.001	***		

Table S1b. Model details for initial GLMM for skin data.

See table details from Table S1a.

Model tested	Fixed effects	Estimate	Std. Error	z value	Pr(>|z|)	p stars	N	DHARMa
timepoint	(Intercept)	-1.809	0.112	-16.123	< 0.001	***	52	ok
	timepointA	-0.183	0.065	-2.834	0.005	**		
built + timepoint	(Intercept)	-1.363	0.195	-6.989	< 0.001	***	52	ok
	Built	-0.011	0.004	-2.642	0.008	**		
	timepointA	-0.187	0.064	-2.904	0.004	**		
pets + timepoint	(Intercept)	-1.871	0.120	-15.610	< 0.001	***	52	ok
	petsTRUE	0.356	0.270	1.319	0.187			
	timepointA	-0.187	0.065	-2.898	0.004	**		
outdoor + timepoint	(Intercept)	-2.016	0.272	-7.410	< 0.001	***	52	ok
	outdoor	0.202	0.242	0.835	0.404			
	timepointA	-0.185	0.065	-2.858	0.004	**		
gardening * timepoint	(Intercept)	-2.054	0.148	-13.839	< 0.001	***	50	ok
	gardeningMONTHLY	0.567	0.207	2.737	0.006	**		
	timepointA	0.232	0.117	1.975	0.048	*		
	gardeningMONTHLY:timepointA	-0.589	0.141	-4.186	< 0.001	***		
real.mat.days*timepoint	(Intercept)	-3.360	0.412	-8.162	< 0.001	***	51	ok
	real.mat.days	0.121	0.030	4.021	< 0.001	***		
	timepointA	1.110	0.397	2.793	0.005	**		
	real.mat.days:timepointA	-0.107	0.027	-3.904	< 0.001	***		
number.of.persons + timepoint	(Intercept)	-2.242	0.252	-8.888	< 0.001	***	51	ok
	number.of.persons	0.142	0.069	2.045	0.041	*		
	timepointA	-0.237	0.066	-3.570	< 0.001	***		
handwashing * timepoint	(Intercept)	-1.823	0.222	-8.209	< 0.001	***	51	ok
	handwashingOFTEN	0.069	0.262	0.264	0.792			
	timepointA	-0.497	0.157	-3.168	0.002	**		
	handwashingOFTEN:timepointA	0.385	0.173	2.231	0.026	*		

Table S1c. Model details for initial GLMM for fecal data.

See table details from Table S1a.

Model tested	Fixed effects	Estimate	Std. Error	z value	Pr(>|z|)	p stars	N	DHARMa
timepoint	(Intercept)	-5.594	0.473	-11.829	<0.001	***	76	ok
	timepointN2	0.275	0.225	1.223	0.221			
	timepointN3	0.186	0.199	0.937	0.349			
built*timepoint	(Intercept)	-6.808	0.876	-7.770	<0.001	***	76	ok
	Built	0.033	0.017	1.999	0.046	*		
	timepointA	1.226	0.557	2.199	0.028	*		
	timepointW	1.224	0.520	2.352	0.019	*		
	Built*timepointA	-0.031	0.018	-1.748	0.081			
	Built*timepointW	-0.033	0.015	-2.165	0.030	*		
pets*timepoint	(Intercept)	-5.636	0.493	-11.425	<0.001	***	76	ok
	petsTRUE	0.213	1.090	0.195	0.845			
	timepointA	0.083	0.274	0.304	0.762			
	timepointW	0.435	0.221	1.970	0.049	*		
	petsTRUE:timepointA	0.341	0.484	0.703	0.482			
	petsTRUE:timepointW	-1.331	0.532	-2.502	0.012	*		
outdoor + timepoint	(Intercept)	-4.263	0.935	-4.560	<0.001	***	76	ok
	outdoor	-1.357	0.901	-1.507	0.132			
	timepointA	0.271	0.224	1.210	0.226			
	timepointW	0.183	0.199	0.921	0.357			
gardening*timepoint	(Intercept)	-5.152	0.559	-9.220	<0.001	***	71	ok
	gardeningMONTHLY	-0.614	0.772	-0.795	0.427			
	timepointA	-0.239	0.301	-0.794	0.427			
	timepointW	0.193	0.257	0.749	0.454			
	gardeningMONTHLY:timepointA	1.350	0.477	2.830	0.005	**		
	gardeningMONTHLY:timepointW	0.003	0.409	0.007	0.994			
real.mat.days + timepoint	(Intercept)	-6.986	0.951	-7.342	<0.001	***	75	ok
	real.mat.days	0.106	0.059	1.792	0.073			
	timepointA	-0.267	0.382	-0.700	0.484			
	timepointW	0.129	0.201	0.645	0.519			
number.of.persons*timepoint	(Intercept)	-6.494	1.049	-6.191	<0.001	***	75	1
	number.of.persons	0.198	0.256	0.774	0.439			
	timepointA	-1.954	1.242	-1.573	0.116			
	timepointW	-2.388	1.196	-1.996	0.046	*		
	number.of.persons:timepointA	0.680	0.382	1.780	0.075			
	number.of.persons:timepointW	0.790	0.373	2.120	0.034	*		
handwashing*timepoint	(Intercept)	-5.451	0.881	-6.187	<0.001	***	74	ok
	handwashingOFTEN	-0.062	0.959	-0.065	0.948			
	timepointA	-0.892	0.583	-1.531	0.126			
	timepointW	0.738	0.362	2.036	0.042	*		
	handwashingOFTEN:timepointA	1.46464	0.6384	2.294	0.022	*		
	handwashingOFTEN:timepointW	-0.75607	0.43297	-1.746	0.081			
